# The Expression and Prognostic Impact of Immune Cytolytic Activity-Related Markers in Human Malignancies: A Comprehensive Meta-analysis

**DOI:** 10.3389/fonc.2018.00027

**Published:** 2018-02-21

**Authors:** Constantinos Roufas, Dimitrios Chasiotis, Anestis Makris, Christodoulos Efstathiades, Christos Dimopoulos, Apostolos Zaravinos

**Affiliations:** ^1^Department of Life Sciences, Biomedical Sciences Program, School of Sciences, European University Cyprus, Nicosia, Cyprus; ^2^The Center for Risk and Decision Sciences (CERIDES), Department of Computer Sciences, School of Sciences, European University Cyprus, Nicosia, Cyprus

**Keywords:** granzyme A, perforin 1, immune cytolytic activity, metastasis, cancer immunotherapy, survival rate, tumor-infiltrating lymphocytes, tumor-associated neutrophils

## Abstract

**Background:**

Recently, immune-checkpoint blockade has shown striking clinical results in different cancer patients. However, a significant inter-individual and inter-tumor variability exists among different cancers. The expression of the toxins granzyme A (GZMA) and perforin 1 (PRF1), secreted by effector cytotoxic T cells and natural killer (NK) cells, were recently used as a denominator of the intratumoral immune cytolytic activity (CYT). These levels are significantly elevated upon CD8+ T-cell activation as well as during a productive clinical response against immune-checkpoint blockade therapies. Still, it is not completely understood how different tumors induce and adapt to immune responses.

**Methods:**

Here, we calculated the CYT across different cancer types and focused on differences between primary and metastatic tumors. Using data from 10,355, primary tumor resection samples and 2,787 normal samples that we extracted from The Cancer Genome Atlas and Genotype-Tissue Expression project databases, we screened the variation of CYT across 32 different cancer types and 28 different normal tissue types. We correlated the cytolytic levels in each cancer type with the corresponding patient group’s overall survival, the expression of several immune-checkpoint molecules, as well as with the load of tumor-infiltrating lymphocytes (TILs), and tumor-associated neutrophils (TANs) in these tumors.

**Results:**

We found diverse levels of CYT across different cancer types, with highest levels in kidney, lung, and cervical cancers, and lowest levels in glioma, adrenocortical carcinoma (ACC), and uveal melanoma. GZMA protein was either lowly expressed or absent in at least half of these tumors; whereas PRF1 protein was not detected in almost any of the different tumor types, analyzing tissue microarrays from 20 different tumor types. CYT was significantly higher in metastatic skin melanoma and correlated significantly to the TIL load. In TCGA-ACC, skin melanoma, and bladder cancer, CYT was associated with an improved patient outcome and high levels of both GZMA and PRF1 synergistically affected patient survival in these cancers. In bladder, breast, colon, esophageal, kidney, ovarian, pancreatic, testicular, and thyroid cancers, high CYT was accompanied by upregulation of at least one immune-checkpoint molecule, indicating that similar to melanoma and prostate cancer, immune responses in cytolytic-high tumors elicit immune suppression in the tumor microenvironment.

**Conclusion:**

Overall, our data highlight the existence of diverse levels of CYT across different cancer types and suggest that along with the existence of complicated associations among various tumor-infiltrated immune cells, it is capable to promote or inhibit the establishment of a permissive tumor microenvironment, depending on the cancer type. High levels of immunosuppression seem to exist in several tumor types.

## Introduction

In normal cells, the role of cytotoxic T lymphocyte antigen-4 (CTLA-4 or CD152), programmed death-1 (PD-1 or CD279), or other similar immune-checkpoint molecules is to inhibit an autoimmune response and restrict an immune cell-mediated tissue damage. Cancer cells on the other hand, regularly use these immune-checkpoint molecules to escape from being detected and eliminated by the cells of the immune system (1–3).

Cytotoxic T cells (CTLs) and natural killer (NK) cells release perforin 1 (PRF1), granzymes, and granulysin, upon their expose to infected or dysfunctional somatic cells. The first cytotoxin polymerizes and creates a channel in the membrane of the target cell. Through these pores, granzymes will then enter the cytoplasm and trigger a caspase cascade, composed of cysteine proteases that will ultimately lead to apoptosis (4, 5). However, apoptosis can also be induced *via* cell–surface interaction between the CTL and the infected cell. Upon the activation of a CTL, the FAS ligand (FasL or CD95L) is expressed on its surface, and it binds to Fas (CD95) being expressed on the target cell (6). Furthermore, the TNF-related apoptosis-inducing ligand (TRAIL) and its receptors (TRAILR1/2) constitute another important axis of immune cytolytic activity (CYT) that leads to apoptosis (7).

Apart from tumor cells, the tumor microenvironment contains many different immune cell types, including neutrophils, macrophages, dendritic cells (DCs), NK cells, T and B cells (8–10). Spontaneous tumor immunity due to the infiltration of such immune cells to the tumor site (11) and immunotherapy can be used to predict the patient outcome in cancer (12–14). However, it is now known that these nonmalignant tumor-infiltrating immune cells can also contribute to cancer by taking part in the modulation of the tumor microenvironment together with other nonimmune stromal cells, including fibroblasts and endothelial cells (15–17).

Immunotherapies that depend on the blockade of such immune-checkpoint molecules can stimulate an anticancer response (18–21). Among them, PD-1 targeting drugs (Pembrolizumab and Nivolumab) or PD-L1 (Atezolizumab, Avelumab, and Durvalumab), and CTLA-4 inhibitors (Ipilimumab) can benefit treatment of several cancer types, comprising skin melanoma, non-small cell lung cancer, kidney cancer, bladder cancer, head and neck cancer, and Hodgkin lymphoma (22–24). Nevertheless, success rate varies from one tumor type to other and some cancers do not respond to therapy or they gradually develop resistance to it.

The interactions between cancer cells and cells of the immune system can be further understood using high-dimensional genomic and transcriptomic datasets stored in online repositories. One such publically available repository is The Cancer Genome Atlas (TCGA),^1^ which contains comprehensive, multi-dimensional maps of the key genomic changes in 33 different cancer types. Latest analysis of the TCGA datasets has linked the genomic landscape of tumors with tumor immunity, implicating neoantigen load in driving T-cell responses (25), and identifying somatic mutations associated with immune infiltrates (26). The Human Protein Atlas (HPA)^2^ (27–30) is another open access platform that provides a map to all the human proteins in cells, tissues, and organs, and integrates different “omics” technologies, such as antibody-based imaging, mass spectrometry-based proteomics, transcriptomics, and systems biology.

Here, we have used a large number of TCGA and HPA datasets containing thousands of solid tumor samples to understand how different cancers induce and adapt to immune responses. RNA-seq data for the genes of interest were extracted from different datasets in Fragments Per Kilobase Million (FPKM) and subsequently transformed to Transcripts Per Kilobase Million (TPM) values using the formula TPMi = FPKMi/sum(FPKMj) × 10^6^. We have further supported the RNA-level information using protein-level data across all cancer datasets. The CYT from each dataset has been further associated with the corresponding patient group’s overall survival. To associate the CYT with patient survival both in primary and metastatic cancers, we have focused our attention on skin melanoma, breast, and thyroid cancers. We have also evaluated the density of tumor-infiltrating lymphocytes (TILs) and tumor-associated neutrophils (TANs) using hematoxylin and eosin (H&E)-stained sections of primary and metastatic tumors and made associations of their load with patient survival in each type of cancer.

## Materials and Methods

### Cancer Datasets

Using the Genomic Data Commons (GDC) Data Portal (The Cancer Genome Atlas, TCGA program^3^) and the GTEx web portal (Genotype-Tissue Expression project^4^), we extracted data from a total of 10,355 tumor resection samples and 2,935 normal samples and screened the variation of CYT across these 32 different cancer types and 28 different normal solid tissue types. TCGA-derived data represent mainly untreated primary tumors (*n* = 9,913). In addition, we extracted 47 recurrent and 395 metastatic cancer cases. The Skin Cutaneous Melanoma (SKCM) dataset included the majority of these metastatic cases (*n* = 368). Patients who received neoadjuvant therapy were excluded from the analysis. Where available, TCGA tumor samples were paired with their corresponding normal tissues, providing a germline reference.

In specific, the following tumor types were selected: diffuse large B-cell lymphoma (DLBCL, *n* = 48), kidney clear cell cancer (KIRC, *n* = 539), kidney papillary cancer (KIRP, *n* = 289), kidney chromophobe cancer (KIRCH, *n* = 65), testicular germ cell cancer (TGCT, *n* = 156), lung adenocarcinoma (LUAD, *n* = 535), lung squamous cell carcinoma (LUSC, *n* = 502), cervical squamous cell carcinoma and endocervical adenocarcinoma (CESC, *n* = 306), thymoma (THYM, *n* = 119), (SKCM, *n* = 471), acute myeloid leukemia (LAML, *n* = 151), head and neck squamous cell carcinoma (HNSC, *n* = 502), pleural mesothelioma (MESO, *n* = 86), sarcoma (SARC, *n* = 263), stomach adenocarcinoma (STAD, *n* = 375), colorectal cancer (COAD, *n* = 480), and rectum adenocarcinoma (READ, *n* = 167), uterine corpus endometrial carcinoma (UCEC, *n* = 552), uterine carcinosarcoma (UCS, *n* = 56), bladder cancer (BLCA, *n* = 414), pancreatic cancer (*n* = 178), breast cancer (BRCA, *n* = 1109), bile duct cancer (*n* = 36), ovarian serous cystadenocarcinoma (OV, *n* = 379), liver hepatocellular carcinoma (LIHC, *n* = 374), thyroid carcinoma (THCA, *n* = 510), esophageal cancer (*n* = 162), prostate adenocarcinoma (PRAD, *n* = 499), glioblastoma (GBM, *n* = 169), brain lower grade glioma (LGG, *n* = 529), pheochromocytoma and paraganglioma (PCPG, *n* = 183), adrenocortical carcinoma (ACC, *n* = 79), and uveal melanoma (UVM, *n* = 80) (where each acronym denotes the corresponding project’s code and “*n*” is the number of cancer tissue samples).

“Level 3” mRNA-Seq expression data of the genes of interest, along with the corresponding patient clinical information for each disease type (tumors and normals) were extracted from TCGA public access web portal [launch data portal^3^] and GTEx^4^ (for normal samples only). Gene expression data were additionally accessed from the Fantom5 Consortium^5^ and were used to evaluate gene expression markers.

We also retrieved protein expression data derived from antibody-based protein profiling using immunohistochemistry (IHC) from the Tissue Atlas of The Human Protein Atlas (HPA) (27–29). Information regarding the cellular distribution of each cytolytic protein (GZMA and PRF1) was also retrieved across all major cancers from the same repository. In total, we extracted IHC data from 19 different tumor types, among them BRCA (*n* = 12), cervical cancer (*n* = 11), colorectal cancer (*n* = 11), endometrial cancer (*n* = 12), glioma (*n* = 12), head and neck cancer (*n* = 3), liver cancer (*n* = 11), lung cancer (*n* = 12), lymphoma (*n* = 12), melanoma (*n* = 12), ovarian cancer (*n* = 12), pancreatic cancer (*n* = 10), prostate cancer (*n* = 10), renal cancer (*n* = 11), skin cancer (*n* = 11), stomach cancer (*n* = 11), testis cancer (*n* = 9), thyroid cancer (*n* = 4), and urothelial cancer (*n* = 11).

### Calculation of CYT Followed by Downstream RNA-seq and Protein Profiling Analyses

We calculated the CYT (or “cytolytic index”) as the geometric mean of GZMA and PRF1, as formerly defined (31). Briefly, we divided the total raw read counts per gene by the gene’s maximum transcript length to signify a coverage depth estimate. Coverage estimates were then scaled to sum to a total depth of 1e^6^ per sample and inferred as Transcripts Per Kilobase Million (TPM). We compared the cytolytic index between metastatic and non-metastatic (primary) cancers, wherever a sufficient number of metastatic tumor cases were available (TCGA-BRCA, TCGA-SKCM, and TCGA-THCA datasets). We also calculated the expression of several other CTL/NK or non-CTL/NK expressing genes, including immunosuppressive factors, the C1Q complex, and interferon-stimulated chemokines, all of which were previously shown to associate with an increased CYT in cancer. We further correlated the cytolytic index with the expression of immune-checkpoint molecules, including CTLA-4, PD-1, CD274 (PD-L1), PDCD1LG2 (PD-L2), LAG3, IDO1, CD73 (NT5E), and ENTPD1 (CD39), across all TCGA datasets. The *p*-values from the comparisons of the CYT between tumor and normal samples or between metastatic and primary cancer samples were FDR-adjusted. Loess regression was applied to diminish the noise of the variables during correlation analysis.

We also extracted GZMA and PRF1 protein expression data from the Tissue Atlas of HPA, and further analyzed them. GZMA was stained with an anti-GZMA antibody produced in rabbit (HPA054134, 1:200 dilution, Sigma-Aldrich) and PRF1 using two different antibodies produced in rabbit (either HPA037940, 1:29 dilution, or CAB002436, 1:10 dilution, Sigma-Aldrich) (27–29).

### Overall Survival and Synergistic Target Analysis on the TCGA Datasets

We performed Kaplan–Meier curves analysis to calculate the overall survival of each TCGA-dataset’s patient group, based on their cytolytic index, TIL, and TAN load, or specific tumor subtype (e.g., triple negative vs triple positive BRCA). In total, we assessed overall survival data of patients suffering from 25 different cancer types (37 TCGA-datasets). Analysis was performed using the log-rank (Mantel Cox) test with a statistical significance at the 95% level (*p* < 0.05). We further tested the synergistic effect of the genes PRF1 and GZMA on each dataset’s patient survival outcome, using SynTarget (32).

### Detection and Quantification of Lymphocyte and Neutrophil Infiltration among Primary and Metastatic Cancers

We extracted digital slide images with H&E-stained histological slides of skin melanoma, breast, and thyroid cancer from The Cancer Digital Slide Archive (CDSA)^6^ and compared the load of TILs and TANs between metastatic and primary cancers. TILs were distinguished by the typical features of lymphocytes (33), including size, shape, and staining of the nucleus. The percentage (%) of lymphocyte and neutrophil infiltration was compared to the information extracted from the corresponding datasets at the GDC Data Portal. We further compared the percentage of necrosis between primary and metastatic cancers, as well as the percentage of tumor, normal, and stromal cells. We correlated the levels of immune CYT (TPM counts) with the load of TILs and TANs, as well as with the percentage of necrosis found among metastatic and non-metastatic breast, skin melanoma, and thyroid cancers, using Pearson’s correlation test.

## Results

### Immune CYT across Different Tumor Types

To assess the intratumoral immune cytolytic T-cell activity across various tumor types, we quantified the transcript levels of GZMA and PRF1, as previously done by Rooney et al. (31). GZMA is a tryptase leading to apoptosis through the caspase pathway, whereas PRF1 is a pore-forming enzyme facilitating the entrance of granzymes into the target cells. Both effector molecules are considerably overexpressed upon CD8+ T-cell activation (34) and during productive clinical responses to anti-CTLA-4 or anti-PD-L1 immunotherapy (12, 13). CTL/NK cells can kill cancer cells by overexpressing GZMA and PRF1. We now know that effector T cells at the tumor site are good predictors of a favorable outcome across various cancer types (35–40).

Although Rooney et al. previously measured the immune CYT of the local immune infiltrate across various tumor types (31), some datasets did not contain enough data at the time (e.g., there were only three normal cervix samples in the TCGA-CESC dataset). Given the increased number of tumor samples in the TCGA platform since 2014, we have now significantly enlarged the total number of different cancer types, from 18 to 32. We have also considerably increased the sample number in many datasets, thus providing an opportunity to better estimate the different cytolytic levels across diverse tumors.

**Figure 1:**
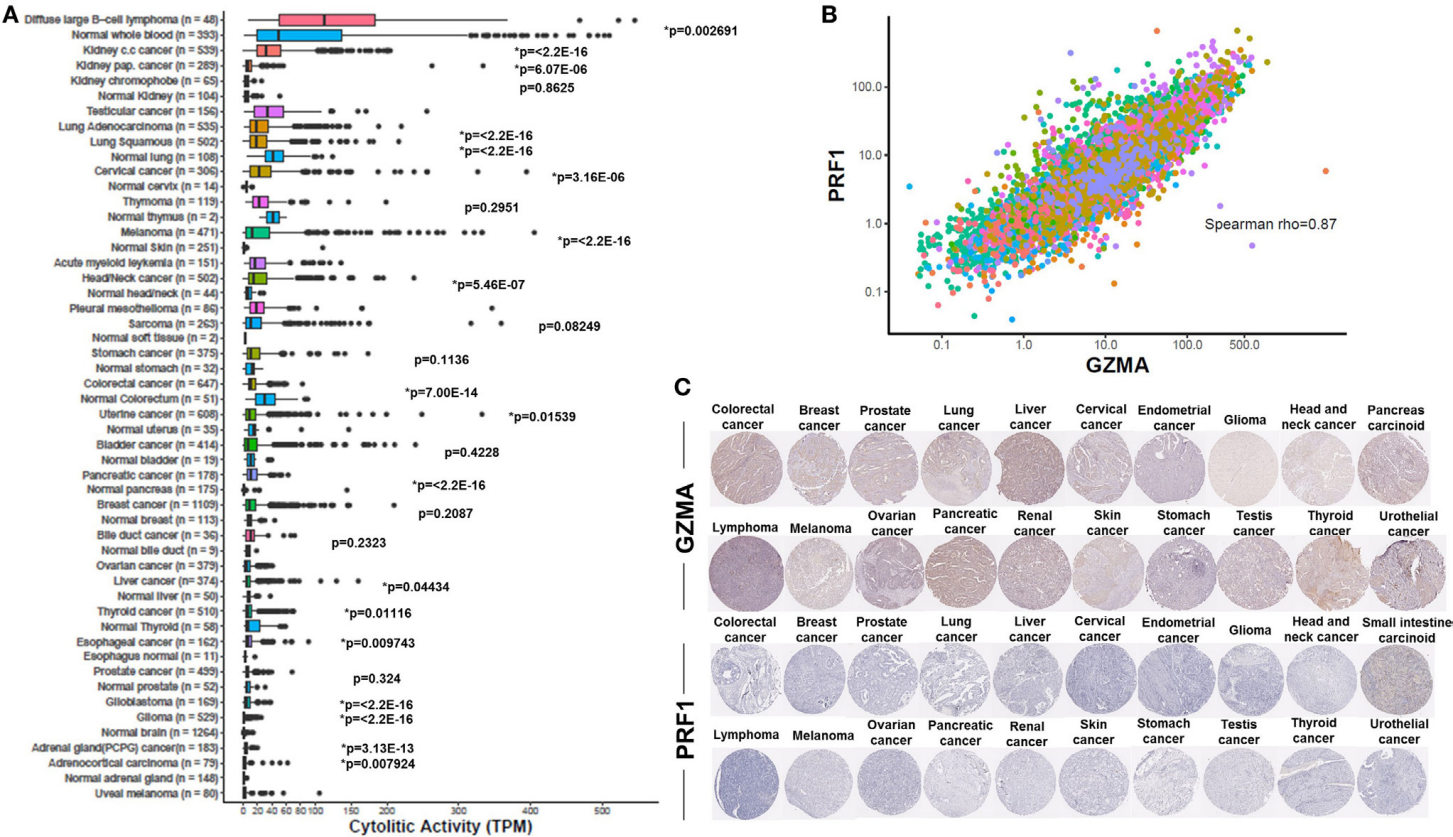
**(A)** Varied immune cytolytic activity for each of 31 different TCGA tumor types and normal tissues. Normal tissue samples are derived both from TCGA and GTEx projects. Boxes in box plot represent interquartile ranges and vertical lines represent 5th–95th percentile ranges, with a notch for the median. *p*-values are adjusted and calculated by Wilcoxon rank-sum test (comparison to relevant normal). Asterisks (*) denote events significant at 10% FDR. **(B)** Granzyme A (GZMA) vs perforin 1 (PRF1) expression across TCGA tumor biopsies. Points are colored according to cancer type using the same color coding employed in Figure 1A. Across all cancers, a Spearman rank correlation (*r*) of 0.87 was observed. **(C)** Low levels GZMA and PRF1 protein expression detected in tissue microarrays of 20 different tumor types. All representative immunohistochemistry images of each tumor type derived from the Human Protein Atlas.

Consistent with previous findings (31, 41), we found that the cytolytic index was highest in the kidney (in clear cell and papillary renal cell carcinoma, but not in chromophobe carcinoma), lung, and cervical cancers. Importantly, we show for the first time that DLBCL and testicular cancer also rank among the top cytolytic active tumors, with DLBCL exhibiting even higher cytolytic levels compared to KIRC (>100 TPM). In addition, melanoma and head and neck cancer exhibited significantly higher CYT compared to the corresponding normal tissues. Acute myeloid leukemia, pleural mesothelioma, sarcoma, and stomach cancer, also exhibited high tendency in CYT. On the contrary, ovarian, liver, thyroid, esophageal, and prostate cancers, as well as glioblastoma, glioma, pheochromocytoma and paraganglioma, adrenocortical carcinoma and uveal melanoma, exhibited the lowest cytolytic indexes (Figure 1A).

Although most normal tissues (11 tissues from TCGA or GTEx) showed significantly lower CYT compared to their corresponding tumors, some of them exhibited significantly higher activity. Specifically, lung cancer, thymoma, stomach, colorectal, uterine, bladder, breast, liver, and thyroid cancers, all exhibited lower CYT compared to their corresponding normal tissues. In the cases of lung adenocarcinoma, colorectal, uterine, liver, and thyroid cancers, the differences between cancer, and the normal tissues were statistically significant (Figure 1A). The vast range in CYT across different cancers and compared to their corresponding normal tissues reveals the existence of a combination of tissue- and tumor-specific mechanisms that control local immunity. In line with their synchronized roles, the expression of GZMA and PRF1 was strongly coordinated across the different cancer samples (Spearman rank correlation, rho = 0.87) (Figure 1B).

At the protein level, we analyzed tissue microarray (TMA) data from 20 different tumor types, and found that GZMA was either lowly expressed or absent in at least half of these tumors, whereas, PRF1 was not detected in almost any of the different tumor types (Figure 1C). GZMA exhibited medium protein expression in the majority of the pancreatic cancers (70%), in <35% of breast, cervical, liver, ovarian, prostate, renal, stomach, testis, and urothelial cancers, as well as in <10% of lymphomas and melanomas. These data are consistent with the low TPM values derived from our RNA-seq analysis (Table 1). Further information regarding anti-GZMA and anti-PRF1 antibody staining, intensity, quantity, and location are provided in Table S1 in Supplementary Material.

**Table 1:** Protein expression profiles of granzyme A (GZMA) and perforin 1 (PRF1) across 19 different cancer types, using antibody-based protein profiling data from immunohistochemistry (the Human Protein Atlas).

	Tumor patients expressing cytolytic genes							
	GZMA				PRF1			
Tumor	High	Medium	Low	Not detected	High	Medium	Low	Not detected
breast cancer	0/12 (0)	4/12 (33)	7/12 (58)	1/12 (8)	1/10 (0)	1/10 (0)	1/10 (0)	10/10 (100)
cervical cancer	0/11 (0)	3/11 (27)	5/11 (45)	3/11 (27)	0/11 (0)	0/11 (0)	1/11 (9)	10/11 (91)
colorectal cancer	0/11 (0)	0/11 (0)	3/11 (27)	8/11 (73)	0/12 (0)	0/12 (0)	0/12 (0)	12/12 (100)
endometrial cancer	0/12 (0)	0/12 (0)	1/12 (8)	11/12 (92)	0/11 (0)	0/11 (0)	0/11 (0)	11/11 (100)
glioma	0/12 (0)	0/12 (0)	0/12 (0)	12/12 (100)	0/12 (0)	0/12 (0)	0/12 (0)	12/12 (100)
head and neck cancer	0/3 (0)	0/3 (0)	1/3 (33)	2/3 (67)	0/4 (0)	0/4 (0)	0/4 (0)	4/4 (100)
liver cancer	0/11 (0)	3/11 (27)	1/11 (9)	7/11 (64)	0/12 (0)	0/12 (0)	0/12 (0)	12/12 (100)
lung cancer	0/12 (0)	0/12 (0)	4/11 (33)	8/12 (67)	0/12 (0)	0/12 (0)	0/12 (0)	12/12 (100)
lymphoma	0/12 (0)	1/12 (8)	1/12 (8)	10/12 (83)	0/11 (0)	0/11 (0)	0/11 (0)	11/11 (100)
melanoma	0/12 (0)	1/12 (8)	6/12 (50)	5/12 (42)	0/11 (0)	0/11 (0)	0/11 (0)	11/11 (100)
ovarian cancer	0/12 (0)	3/12 (25)	2/12 (17)	7/12 (58)	0/10 (0)	0/10 (0)	0/10 (0)	10/10 (100)
pancreatic cancer	0/10 (0)	7/10 (70)	2/10 (20)	1/10 (10)	0/12 (0)	0/12 (0)	0/12 (0)	12/12 (100)
prostate cancer	0/10 (0)	3/10 (30)	6/10 (60)	1/10 (10)	0/11 (0)	0/11 (0)	0/11 (0)	11/11 (100)
renal cancer	0/11 (0)	2/11 (18)	3/11 (27)	6/11 (55)	0/12 (0)	0/12 (0)	0/12 (0)	12/12 (100)
skin cancer	0/11 (0)	0/11 (0)	4/11 (36)	7/11 (64)	0/12 (0)	0/12 (0)	0/12 (0)	12/12 (100)
stomach cancer	0/11 (0)	2/11 (18)	6/11 (55)	3/11 (27)	0/12 (0)	0/12 (0)	0/12 (0)	12/12 (100)
testis cancer	0/9 (0)	2/9 (22)	2/9 (22)	5/9 (56)	0/12 (0)	0/12 (0)	0/12 (0)	12/12 (100)
thyroid cancer	0/4 (0)	0/4 (0)	2/4 (50)	2/4 (50)	0/4 (0)	0/4 (0)	0/4 (0)	4/4 (100)
urothelial cancer	0/11 (0)	3/11 (27)	5/11 (45)	3/11 (27)	0/11 (0)	0/11 (0)	0/11 (0)	11/11 (100)

*Numbers indicate the tumor patients expressing each gene out of the total number (percentage, %)*.

### Immune CYT in Primary and Metastatic Cancers

Next, we focused our attention on whether the cytolytic index differs between primary and metastatic cancers. Among all TCGA datasets, the metastatic tumors were composed of 368 SKCM, seven BRCA, eight THCAs, one PRAD, two cervical cancers (CESC), one colorectal adenocarcinoma (COAD), one esophageal carcinoma (ESCA), two HNSCs, one pancreatic adenocarcinoma (PAAD), two PCPGs, one PRAD, and one sarcoma (SARC) sample. Therefore, since the majority of metastatic tumors were composed mainly of skin melanomas, breast and thyroid carcinomas we focused our downstream analysis on the corresponding datasets of these tumors.

**Figure 2:**
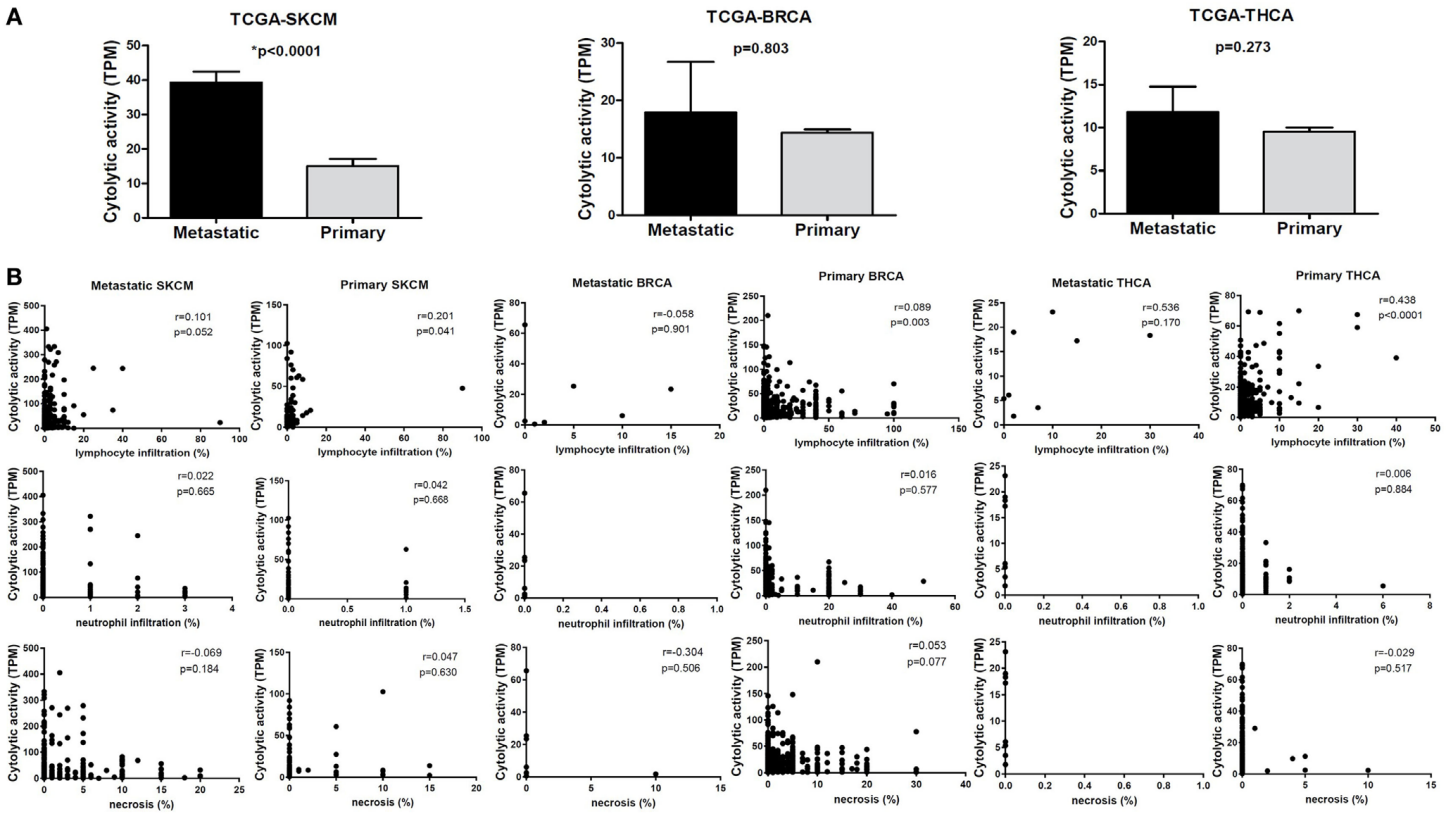
**(A)** Metastatic cancers exhibit higher cytotoxic T cell levels. Significantly, increased levels of immune cytolytic activity (CYT) were scored in metastatic skin melanomas, in the TCGA-SKCM dataset. The cytolytic index was also higher in metastatic breast (BRCA) and thyroid cancers (THCA), but did not differ significantly between metastatic and primary tumors. Bars denote mean ± SEM. **(B)** Pearson’s correlations between CYT and the percentage of tumor-infiltrating lymphocytes and neutrophils, as well as with the percentage of necrosis in primary and metastatic SKCM, BRCA and THCA.

To assess the cytolytic index in them, we obtained RNA-seq data from TCGA for 103 primary and 368 metastatic skin resection melanomas, 1,102 primary and seven metastatic BRCAs, as well as for 502 primary and eight metastatic THCAs. Although all metastatic tumors had higher CYT compared to their corresponding primary tumors, the difference was statistically significant only for the skin melanoma dataset. This is obviously due to the significantly higher number of metastatic melanoma cases (*n* = 368) (Figure 2).

Similarly, we investigated the expression of various suppressive factors previously shown to be associated with CYT, and compared their expression levels between metastatic and primary tumors. These genes included the immune-checkpoint molecules, CTLA-4, PD-1 (PDCD1), PD-L1 (CD274), PD-L2 (PDCD1LG2), LAG3, CD73 (NT5E)/CD39 (ENTPD1), IDO1/2, DOK3, the GMCSF receptors (CSF2RA, CSF2RB) (42), CD70, UBD, DOC3, NKG7, PLA2G2D, and the C1Q complex. We also included interferon-stimulated chemokines that attract T cells (CXCL9, CLCL10, and CXCL11) (11). We further investigated the expression of alternative genes through which T cells can induce cytolysis of cancer cells, including CD95-CD95L (FAS-FASLG) and TRAIL-TRAILR (TNFSF10, TNFRSF10A/B). Among the investigated genes, CD247, GZMK, GZMH, NKG7, PRF1, GZMA, GZMB, GZMH, GZMK, CD3E, and CD2 are expressed in CTL/NK cells; whereas CSF2RB, LTA, DOK3, PDCD1LG2, IDO1, PLA2G2D, CXCL9, CXCL10, CXCL11, CXCL13, UBD, C1QA, C1QB, C1QC, BATF2, and CSF2RA are expressed in non-CTL/NK cells (31).

**Figure 3:**
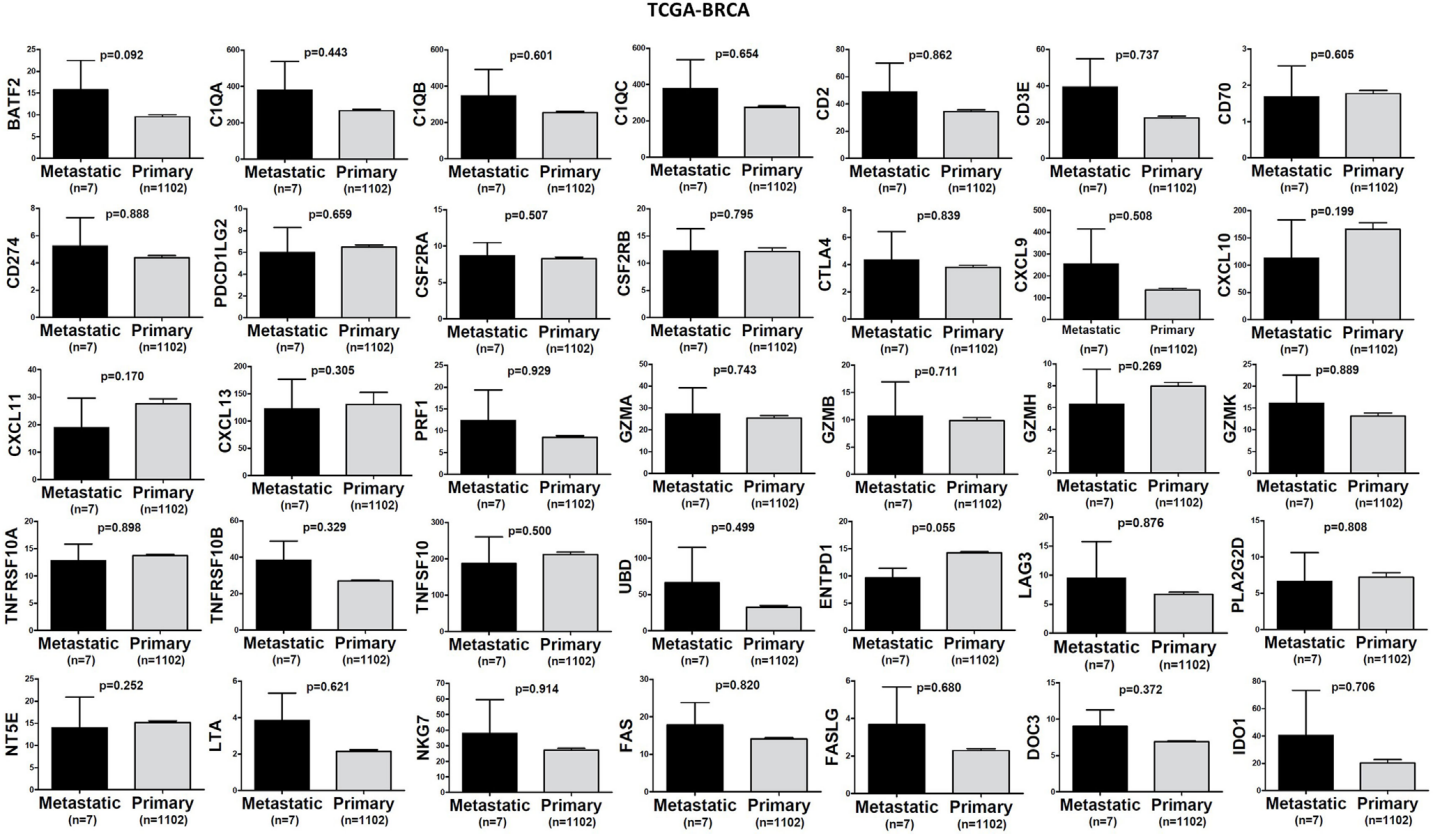
A trend for higher expression (TPM) of a group of genes being expressed in cytotoxic T cell/natural killer (CTL/NK) and non-CTL/NK cells and correlating with cytolytic activity (CYT) in metastatic breast cancers compared to primary tumors. Bars denote mean ± SEM.

**Figure 4:**
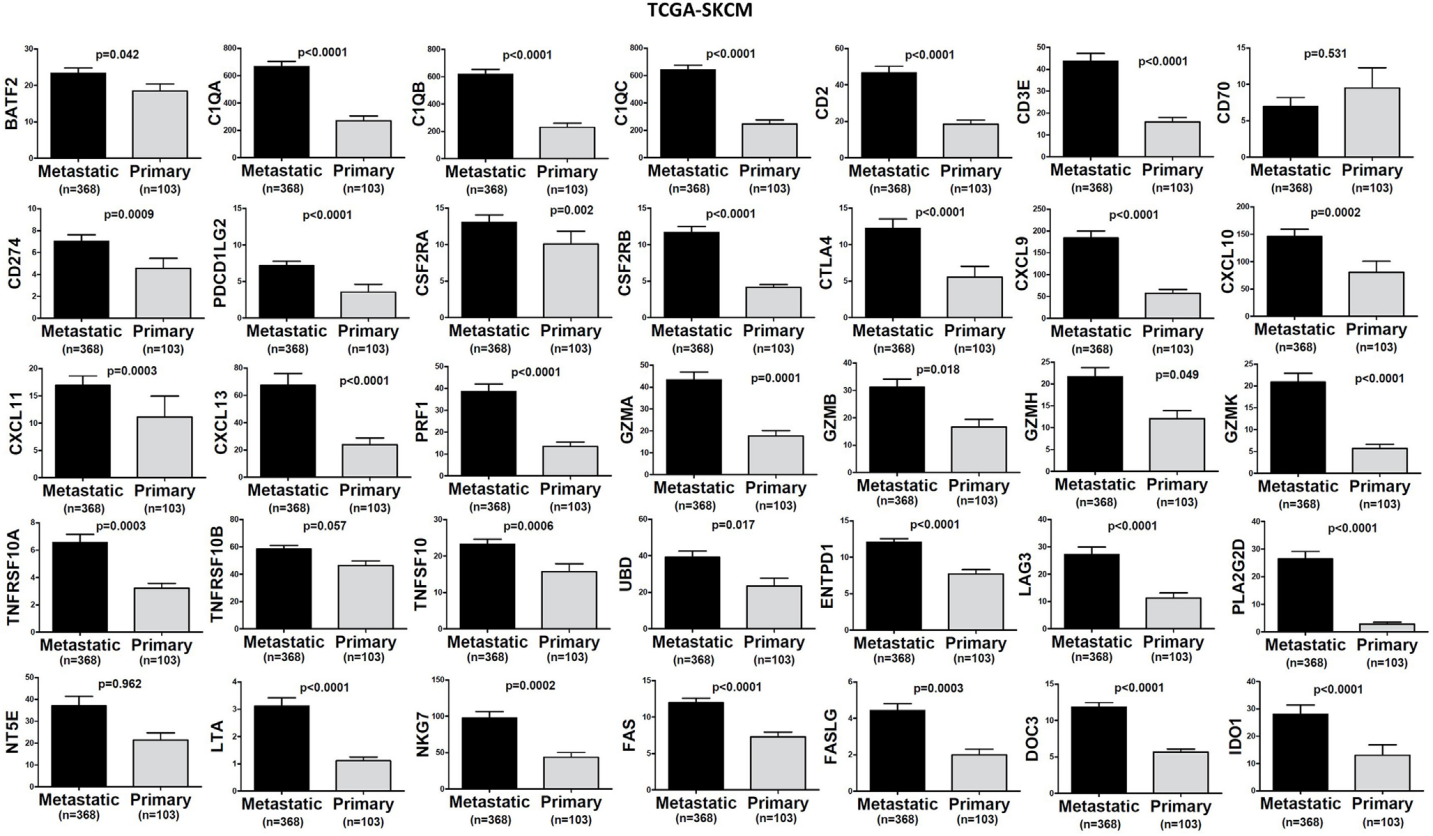
Significantly higher expression (TPM) of a group of genes being expressed in cytotoxic T cell/natural killer (CTL/NK) and non-CTL/NK cells and correlating with cytolytic activity (CYT) in metastatic skin melanomas compared to primary tumors. Bars denote mean ± SEM.

**Figure 5:**
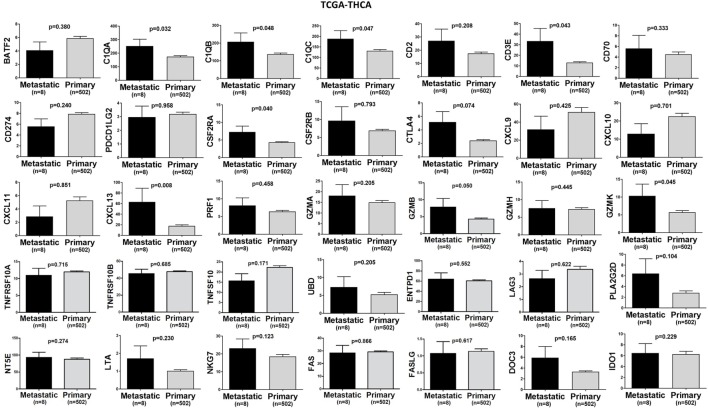
A trend for higher expression (TPM) of a group of genes being expressed in cytotoxic T cell/natural killer (CTL/NK) and non-CTL/NK cells and correlating with cytolytic activity (CYT) in metastatic thyroid cancers compared to primary tumors. Bars denote mean ± SEM.

In TCGA-SKCM, all genes (apart from CD70) exhibited significantly higher levels in metastatic skin melanomas compared to primary tumors. We also noticed a similar, but non-significant trend in datasets TCGA-BRCA and TCGA-THCA, presumably due to the small sample number of metastatic cases (Figures 3–5).

### Kaplan–Meier and Synergistic Survival Analysis of GZMA and PRF1 across TCGA-Datasets

We next performed Kaplan–Meier survival analysis on 37 TCGA-datasets deriving from 25 different cancer types in order to estimate the risk of individual and/or simultaneous high (or low) PRF1 and GZMA expression on patient overall survival.

**Figure 6:**
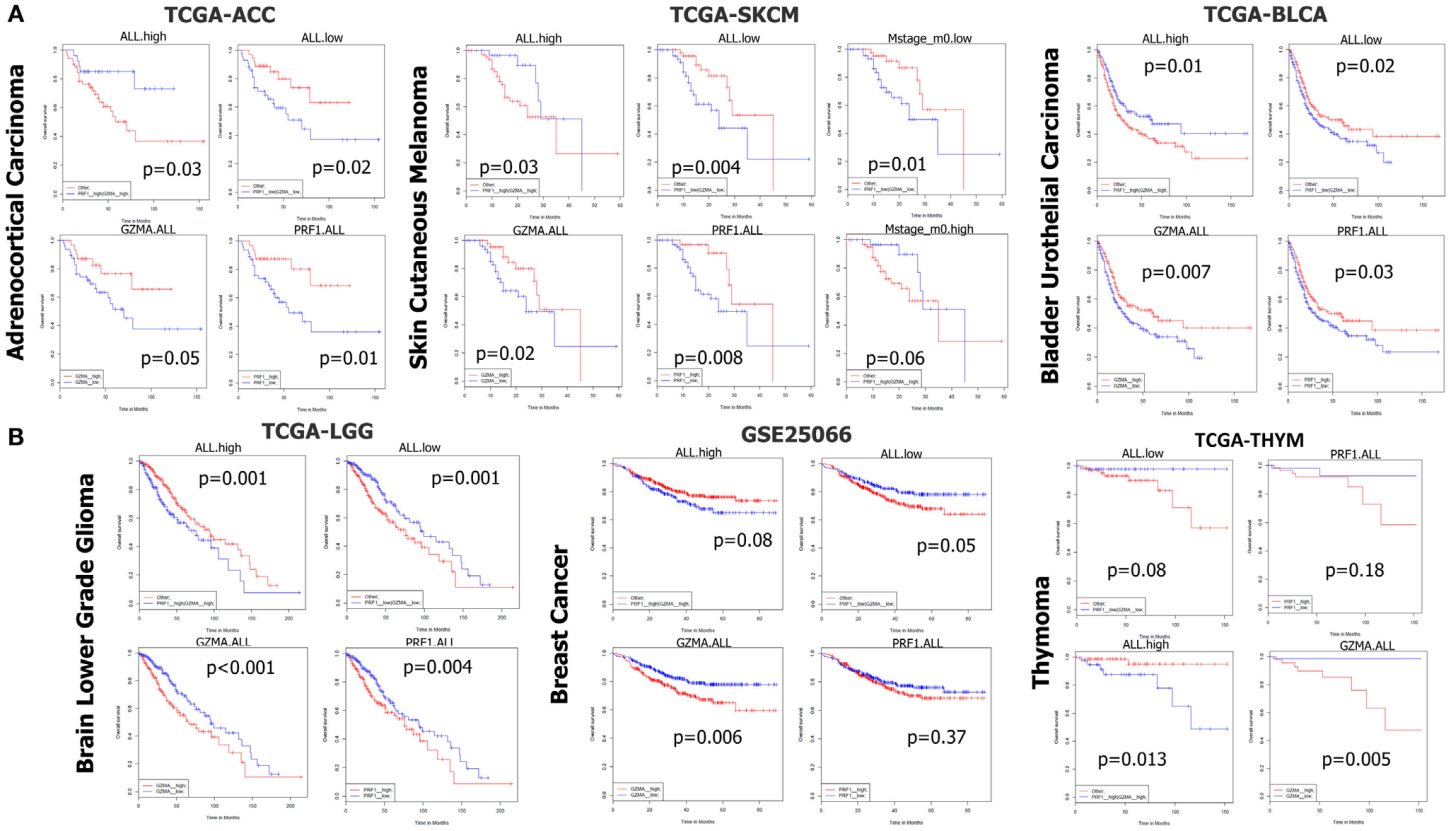
**(A)** In datasets TCGA-ACC, TCGA-SKCM, and TCGA-BLCA, both individual and simultaneous high levels of perforin 1 (PRF1) and granzyme A (GZMA) were significantly associated with an improved prognosis. On the reverse, simultaneous low expression of both cytolytic genes led to a significant shift toward negative effect vs all other patients. **(B)** On the contrary, in datasets TCGA-LGG, GSE25066, and TCGA-THYM, accordingly, both individual and simultaneous high levels of GZMA and PRF1 were significantly associated with a worse prognosis, whereas the simultaneous low levels of both genes led to a significant shift toward positive effect vs all other patients. Abbreviations: “ALL.high,” PRF1 high expression and GZMA high expression vs all others; “ALL.low,” PRF1 low expression and GZMA low expression vs all others; “GZMA.ALL,” GZMA high expression vs GZMA low expression; “PRF1.ALL,” PRF1 high expression vs PRF1 low expression.

In TCGA-ACC, non-metastatic cutaneous melanoma (“m0” TCGA-SKCM), and bladder urothelial carcinoma (TCGA-BLCA but not the GSE32894 dataset), both individual and simultaneous high levels of PRF1 and GZMA were significantly associated with better prognosis. On the reverse, simultaneous low expression of both genes led to a significant shift toward negative effect vs all other ACC (or SKCM) patients. As expected, metastatic melanoma sufferers succumbed much earlier than non-metastatic skin melanoma patients did. These data provide significant evidence that high expression of both cytolytic genes in these cancer types, synergistically affects patient survival (Figure 6A).

In TCGA-LIHC, only the individual high levels of PRF1 and GZMA were significantly associated with a positive effect on patient survival. A similar non-significant association of (individual or simultaneous) high GZMA and PRF1 expression with better effect on patient survival could also be observed in TCGA-MESO, ovarian cancer (GSE13876 and GSE49997), TCGA-STAD, TCGA-THCA, and TCGA-UCEC (Figure S1 in Supplementary Material). These data suggest that high CYT is widely associated with an improved prognosis among the above-mentioned cancer types.

On the contrary, across TCGA-LGG, BRCAs (GSE25066), and TCGA-THYM, both individual and simultaneous high levels of GZMA and PRF1 were significantly associated with a worse prognosis, whereas the simultaneous low levels of both genes led to a significant shift toward positive effect (Figure 6B). Regarding BRCA, though, we could not confirm these results using the independent datasets METABRIC and TCGA-BRCA, which revealed a tendency for the opposite effects of both cytolytic genes on patient survival. Regarding the METABRIC dataset, we separated BRCA patients who were subjected to hormonal therapy plus radiotherapy (HT/RT) (*n* = 605) from the untreated patients; however, an association of high levels of GZMA and PRF1 with a worse prognosis could not be confirmed (Figure S6 in Supplementary Material).

Analogous non-significant associations of (individual or simultaneous) high cytolytic levels with worse effect on patient survival were also observed in lung cancer (GSE30219, TCGA-LUAD, and TCGA-LUSC), TCGA-PAAD, TCGA-PRAD and GSE16560, and TCGA-READ (Figure S2 in Supplementary Material).

In colon cancer, neither the individual nor the simultaneous high levels of the two genes were associated with a better prognosis, although simultaneous low levels of GZMA and PRF1 tended to shift toward a negative effect. Depending on the probe used, it seemed that a combination of high PRF1 and low GZMA levels yields a better patient outcome (GSE39582, TCGA-COAD, TCGA-COADREAD). Among metastatic colon cancer patients (“M1” patients in the TCGA-COAD dataset), simultaneous high levels of both genes were marginally significantly associated with worse prognosis, but simultaneous low levels of both genes could not provide the reverse trend (Figure S3 in Supplementary Material). We did not notice the same trend in the TCGA-COADREAD colorectal cancer patient cohort, though, implying that the aforementioned results are specific for colon (but not rectum) cancers.

Among clear-cell (TCGA-KIRC) and papillary renal cell carcinomas (TCGA-KIRP), we could not deduce any similar association among metastatic or non-metastatic tumors. In chromophobe renal carcinoma (TCGA-KICH) though, individual and simultaneous high levels of both genes tended to associate with better patient survival. On the other hand, concurrent low levels of both cytolytic genes, tended to associate with a worse prognosis. Interestingly, in the TCGA-KIPAN dataset, both the individual and synchronized high levels of GZMA and PRF1 significantly connected with worse patient survival. The simultaneous low expression of both genes exhibited reverse outcome (Figure S4 in Supplementary Material).

In DLBCL (GSE10846, and GSE32918), using various combinations of distinct molecular probes for the two cytolytic genes (PRF1, 214617_AT, 1553681_A_AT, or ILMN_1740633; GZMA, 205488_AT, or ILMN_1779324), we could not provide any significant association with patient survival. A similar absence of significant associations was also detected in glioblastoma (GSE4271, GSE13041, and TCGA-GBM) and non-metastatic HNSCs. We could not deduce any further association or trend between the expression of both cytolytic genes and the survival of TCGA-TGCT and uterine carcinosarcoma patients (Figure S5 in Supplementary Material).

### Infiltration of Lymphocytes and Neutrophils in Primary and Metastatic TCGA-Datasets

We further evaluated the infiltration of lymphocytes (TILs) and neutrophils (TANs) to the tumor site of primary and metastatic cancer samples across the TCGA-SKCM, TCGA-BRCA, and TCGA-THCA datasets, using the Cancer Digital Slide Archive (see text footnote 6). TILs contained both stromal- and intratumoral-compartment lymphocytes, as previously defined (43). Both of them were mainly composed of T cells and a smaller number of B cells, NK cells, and macrophages (44, 45).

In the TCGA-BRCA dataset, the number of TILs appeared enriched in the stroma of the primary tumors compared to the corresponding areas on the slide of the metastatic BRCAs. However, this might probably be due to the higher number of stroma cells detected in the primary breast tumors (percentage of stromal cells in primary vs metastatic BRCA, 21.15 ± 0.520 vs 7.143 ± 3.595; *p* = 0.032).

**Figure 7:**
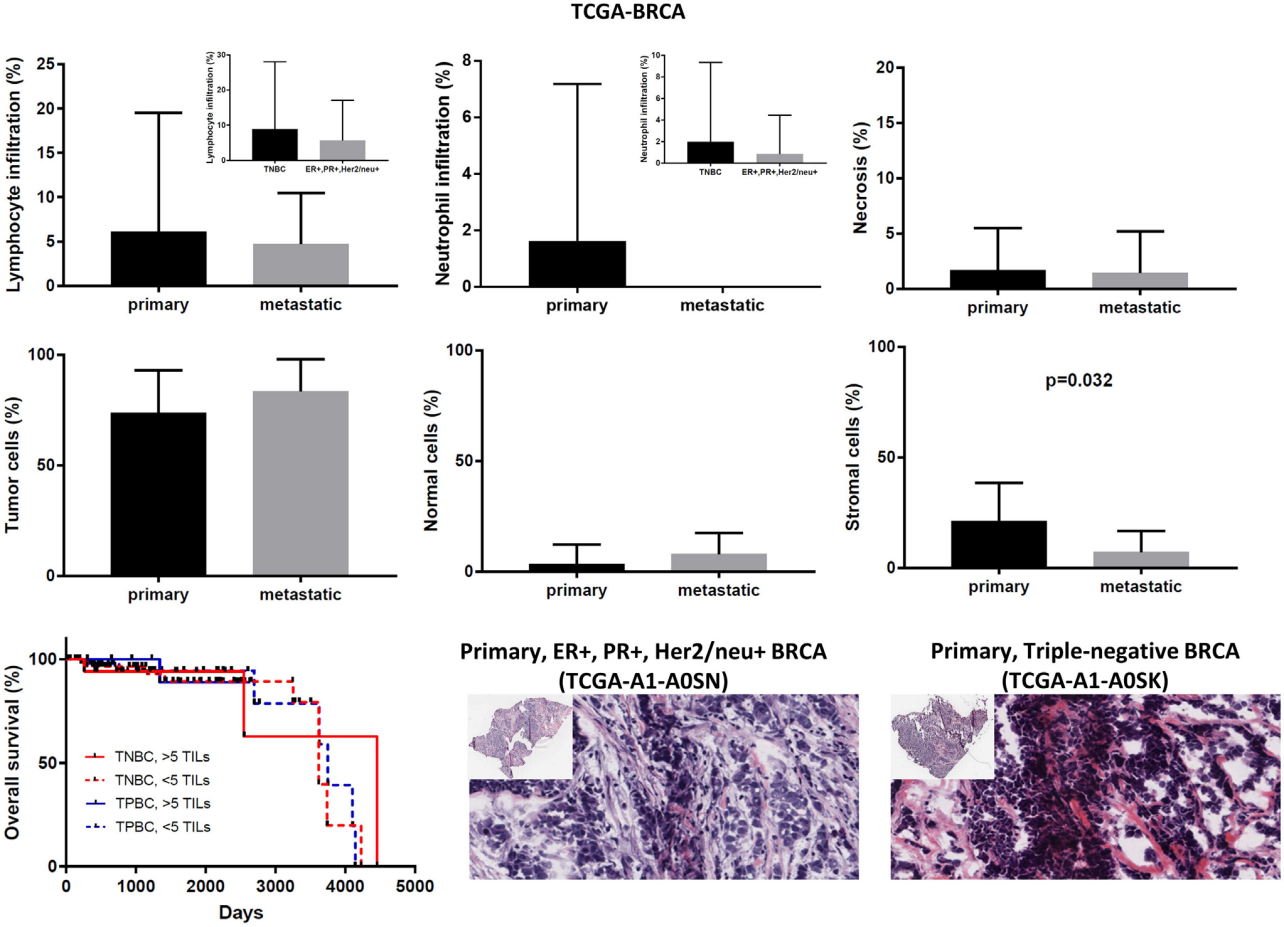
Overall percentages of lymphocyte and neutrophil infiltration and necrosis (upper part); overall percentage of tumor, normal, and stromal cells (middle part); overall patient survival with respect to the percentage of tumor-infiltrating lymphocytes (TILs) (>5%, high TILs, <5%, low TILs); and representative hematoxylin and eosin slides of ER+, PR+, Her2/neu+ (TPBC) and triple negative breast cancer (lower part).

Although the number of TILs and neutrophils was higher in several cases of primary BRCA, the overall difference was not statistically significant (mean% of TILs ± SD in primary vs metastatic BRCAs, 6.102 ± 0.403 vs 4.714 ± 2.179; *p* = 0.78 and mean% of neutrophil infiltration ± SD in primary vs metastatic BRCAs, 1.625 ± 0.167 vs 0 ± 0; *p* = 0.44). Among primary tumors, comparing between triple negative (ER−, PR−, Her2/neu−, or TNBC), and triple positive (ER+, PR+, Her2/neu+, or TPBC) BRCAs, the load of TILs (and TANs) was not significantly different and was not significantly associated with a worse outcome, in argument with previous observations (46–48). In addition, the percentage of necrosis did not differ between metastatic and primary skin melanoma (<2%) (Figure 7).

**Figure 8:**
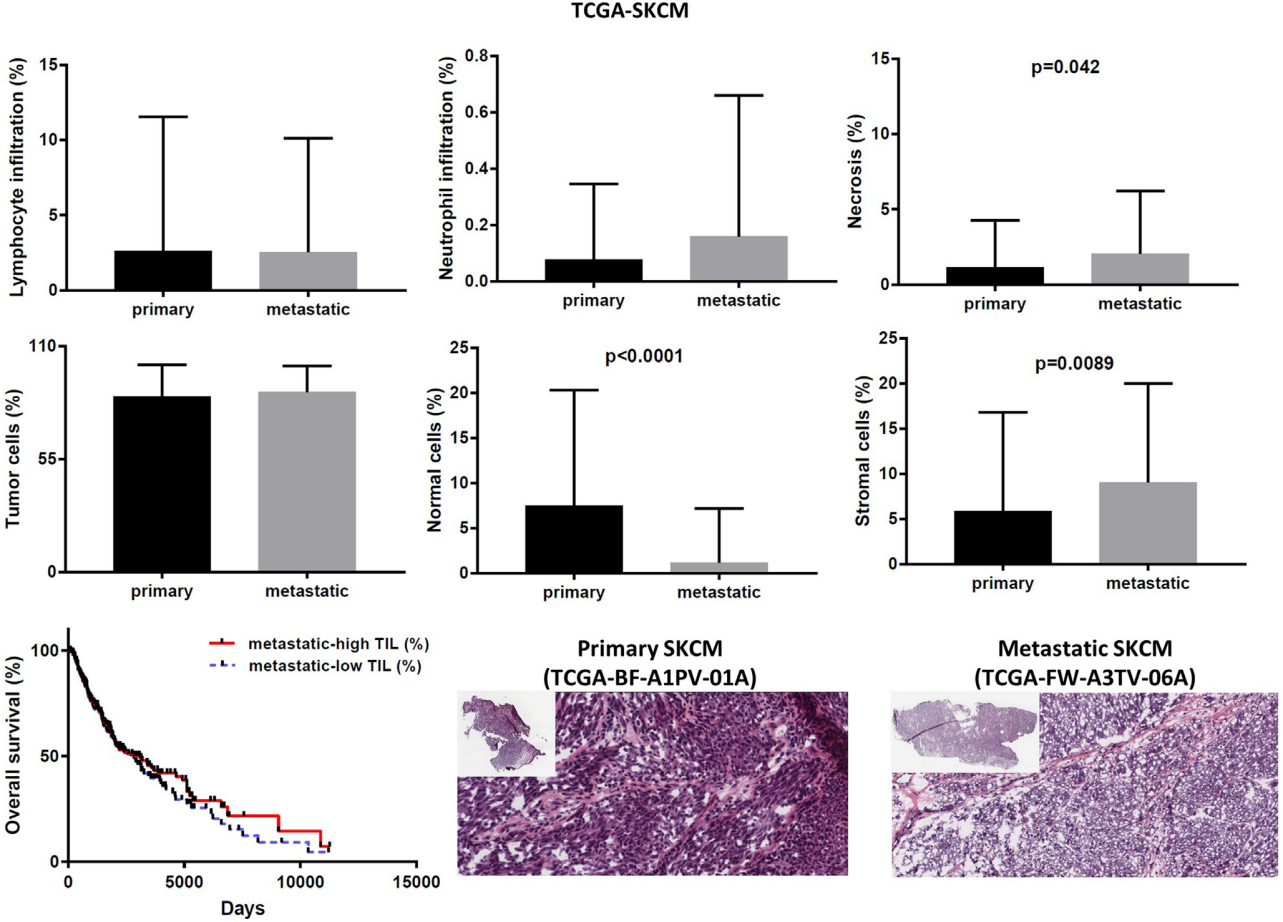
Overall percentages of lymphocyte and neutrophil infiltration and necrosis (upper part); overall percentage of tumor, normal, and stromal cells (middle part); overall patient survival with respect to tumor-infiltrating lymphocytes and representative hematoxylin and eosin slides of primary and metastatic skin melanomas (lower part).

In the TCGA-SKCM dataset, although in several cases the number of TILs was more enriched in the stroma of primary melanomas (as opposed to metastatic cancers), the overall load of TIL and TAN did not differ significantly between them. It is also worth noticing that the number of stroma cells counted in metastatic melanomas was higher compared to primary skin tumors (percentage of stromal cells in primary vs metastatic melanoma, 5.835 ± 1.083 vs 9.043 ± 0.571; *p* = 0.009). In addition, the rate of necrosis was marginally higher in metastatic skin melanoma compared to primary tumors (*p* = 0.042). The overall survival did not differ between high TIL load (>1% TILs) or low TIL load (<1% TILs) in primary skin melanoma patients. However, among metastatic patients, a high percentage of lymphocytic infiltration shifted toward a better prognosis (Figure 8). According to recent data, the number of TILs in stage III metastatic melanoma associates with the response to Ipilimumab once these patients progress to stage IV disease (49).

**Figure 9:**
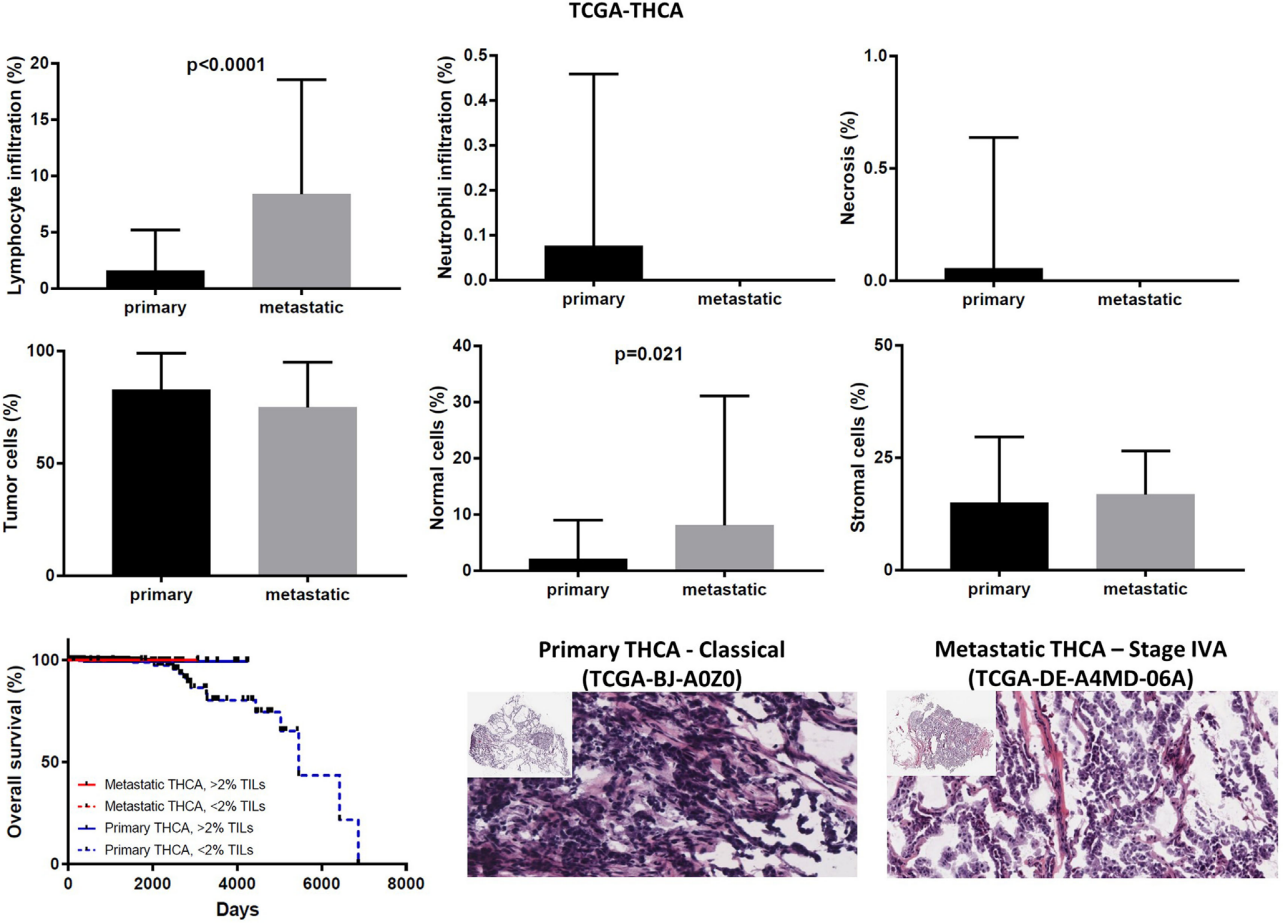
Overall percentages of lymphocyte and neutrophil infiltration and necrosis (upper part); overall percentage of tumor, normal, and stromal cells (middle part); overall patient survival in respect to tumor-infiltrating lymphocytes and representative hematoxylin and eosin slides of primary and metastatic thyroid carcinomas (lower part).

In the TCGA-THCA dataset, the infiltration of lymphocytes was significantly higher in metastatic thyroid tumors and the high TIL load (>2% TILs) was associated with a better prognosis within the primary tumor group (mean% of TILs ± SD in primary vs metastatic cancers, 1.597 ± 0.160 vs 8.375 ± 3.59, *p* < 0.0001). The infiltration of neutrophils was minor (<0.1%) and did not differ between primary and metastatic THCAs. The necrotic rate was equally low between the two groups (Figure 9).

### Correlation of the Cytolytic Index with Immune-Checkpoint Molecules and TILs in Primary and Metastatic TCGA-Datasets

In order to understand the context of PRF1/GZMA deregulation relative to the expression of various immune-checkpoint molecules, we correlated the cytolytic index with the expression of CTLA-4, PD-1, CD274 (PD-L1), PDCD1LG2 (PD-L2), LAG3, IDO1, CD73 (NT5E), and CD39 (ENTPD1) across all TCGA datasets (Figure S7 in Supplementary Material). In the majority of the cancers, a high cytolytic index was accompanied by upregulation of at least one immune-checkpoint molecule, indicating that similar to melanoma (42) and prostate cancer (41), immune response in CYT-high tumors elicits multiple host and tumor mechanisms of immune suppression in the tumor microenvironment (Figure S7 in Supplementary Material). For example, in TCGA-BRCA, CTLA-4, and PD-1 expression was significantly associated with a high cytolytic index (CTLA-4, *p* = 8.75e−199, Pearson’s rho = 0.75; PD-1, 4.09e−309, Pearson’s rho = 0.85). As expected, this correlation was 20 times stronger compared to the normal breast, due to absence of immunosuppression in the latter (CTLA-4, *p* = 2.44e−12, Pearson’s rho = 0.60; PD-1, 1.12e−14, Pearson’s rho = 0.65). Importantly, this association was even stronger among metastatic melanomas, suggesting the existence of a more intense immunosuppression in these tumors (e.g., in primary melanoma, PD-1, *p* = 1.14e−35, Pearson’s rho = 0.887; LAG3, *p* = 1.03e−45, Pearson’s rho = 0.930; IDO1, *p* = 3.05e−08, Pearson’s rho = 0.513. In metastatic melanoma, PD-1, *p* = 1.48e−148, Pearson’s rho = 0.917; LAG3, *p* = 4.28e−163, Pearson’s rho = 0.931; IDO1, *p* = 2.38e−53, Pearson’s rho = 0.690) (Figure S8 in Supplementary Material).

The cytolytic index was significantly correlated with lymphocyte infiltration in BRCA, thyroid cancer, and skin melanoma. The association between a high TIL load and CYT was stronger among primary breast and THCAs, but not in melanomas. Consistent with the fact that apoptosis is a hallmark of CYT, we scored no further correlation between CYT and necrosis or between CYT and infiltration of neutrophils (Figure 2B).

### Number of Tumor and Normal Cells across Metastatic and Non-Metastatic TCGA-Datasets

Expression analysis can be hampered due to a different number of cells within each tumor, thus reducing the ability to confidently measure the cytolytic index and correlate it with the expression of immune-checkpoint molecules in each dataset, as well as to compare gene expression between primary and metastatic cancers. To address this, we calculated the number of tumor and normal cells within each tumor. Overall, the primary and metastatic cancer samples across the three datasets contained an equal number of tumor cells (70–90% tumor cells, *p* > 0.05) (Figures 7–9). Thus, the detected differences should not be the result of enrichment in tumor cells in one group or the other. Similarly, the percentage in normal cells did not differ between primary and metastatic BRCAs (3.48 ± 0.26, in primary cancers vs 7.86 ± 3.56, in metastatic cancers; *p* = 0.188). On the other hand, primary melanomas had higher percentage of normal cells compared to metastatic tumors (7.485 ± 1.263 vs 1.155 ± 0.313, *p* < 0.001), and metastatic THCAs had a higher percentage of normal cells compared to their primary counterparts (8.125 ± 8.125 vs 2.126 ± 0.305, *p* = 0.021).

## Discussion

In this study, we quantified the cytolytic index based on the expression of GZMA and PRF1, both of which mediate cytolysis. This index is strongly associated with CTLs, plasmacytoid dendritic cells, counter-regulatory Tregs, and known T-cell co-inhibitory receptors (31).

In agreement with Rooney et al. (31), we found a great variation in the immune CYT across different types of cancer, which possibly reflects the existence of merged tissue- and tumor-specific mechanisms orchestrating the local immunity. Cancers of the ovaries, liver, thyroid, esophagus, and prostate, as well as glioblastoma, glioma, pheochromocytoma and paraganglioma, adrenocortical carcinoma and uveal melanoma all exhibited minimal levels of CYT. On the contrary, DLBCL, clear-cell renal cell carcinoma, testicular cancer, cervical cancer, skin melanoma, and head and neck carcinoma exhibited increased levels of CYT.

The tumor-intrinsic resistance to CYT has been suggested to be due to different mechanisms. Among them, recurrent mutations in immune-related genes have been proposed, such as B2M, HLA-A, -B, and -C, and CASP8, as well as copy number aberrations in loci containing immunosuppressive factors, including the receptors PD-L1/2 and CTLA-4 (31). PD-1 is transiently induced in activated T cells (50) and its expression is preserved in TILs (51–53). PD-L1 expression is high in several human malignancies, such as skin melanoma, lung, head and neck, and ovarian cancers (54, 55). PD-L1 expression is also correlated with a bad prognosis among patients with esophageal, colon, ovarian, or kidney cancer (56–60). The PD-1/PD-L1 axis is significant in tumor-induced immune evasion and both molecules are hopeful target candidates for immunotherapy. Actually, recent clinical trials have demonstrated that blockage of this signaling can benefit patients with advanced melanoma, kidney, or non-small cell lung cancer (2, 61–63). In metastatic melanoma, PD-L1 expression on peripheral T cells was recently shown to be prognostic on overall and progression-free survival (64).

CTLA-4 expression levels are low on resting T cells, but increase upon T-cell activation. In acute infection, CTLA-4 is transiently induced and binds to B7-1/2, thus competing with CD28 and weakening the T-cell response (65). On the other hand, CTLA-4 is constitutively expressed in T cells during chronic infection and cancer due to chronic antigen exposure. CTLA-4 is also constitutively expressed on antigen-experienced memory CD4+ and CD8+ T cells, as well as Tregs (65). Similarly, B7-1 is not expressed on resting antigen-presenting cells (APCs) (as opposed to B7-2) and is induced after APC activation. Anti-CTLA-4 therapy (Ipilimumab) was shown to induce cancer regression in metastatic kidney cancer (22, 66) and melanoma (67–70). Importantly, CTLA-4 blockade was reported to associate with bowel inflammation in melanoma patients (71), signifying that its signaling is crucial for the preservation of immune homeostasis in the gut.

Another example of immune-inhibitory molecule is indoleamine-pyrrole 2,3-dioxygenase (IDO). This molecule is constitutively expressed in the tumor microenvironment either by tumor cells or by host immune cells and is stimulated by inflammatory cytokines as IFN-γ, leading to host immune inhibition through increased Treg and effector T-cell proliferation blockade. A combination of IDO inhibition and immune-checkpoint blockade are currently under clinical investigation, with promising results (72).

Arginase is also an immune-inhibitory metabolic enzyme being expressed by both tumor cells as well as infiltrating myeloid cells (73). Both IDO and arginase inhibit immune responses by locally depleting the essential amino acids for anabolic functions in T cells or synthesizing specific natural ligands for cytosolic receptors, which can change the functions of lymphocytes. Inhibition of both IDO and arginase can enhance intratumoral inflammation (74, 75).

We found that high levels of several immune-checkpoint molecules, including CTLA-4, PD-1, PD-L1/2, LAG3, IDO1, CD73, and CD39 are associated with an increased cytolytic index, across many cancers; and we expect that a combinatorial targeting of such immune-checkpoint molecules can provide a synergistic effect in cancer immunotherapy. Garg et al. found that predictive biomarkers of responsiveness to immune-checkpoint inhibitors in glioblastoma (GBM) exhibited inconsistent patterns among patients, predicting either resistance or susceptibility to therapeutic targeting of CTLA-4 or IDO1 (76).

Furthermore, different levels of tumor-intrinsic resistance to CYT can be attributed to the diverse levels of neoepitopes in these tumor types. Neoepitopes are tumor-specific antigens produced from DNA mutations occurring in cancer cells. Such mutations can be missense mutations, indels (insertions/deletions), and/or gene fusions. Increasing evidence shows that neoepitope-specific antitumor immune responses occur naturally in cancer cells and have great potentials as immunotherapeutic agents (77). Theoretically, immune responses to neoepitopes are not diminished by host central tolerance in the thymus and cannot trigger an autoimmune reaction (77, 78). These neoepitopes were lately shown to facilitate recognition of a tumor as foreign (78, 79), and an increased load of them is associated with effective immune responses to immune-checkpoint therapy (80). Currently, strategies to selectively enhance T-cell reactivity against genetically defined neoepitopes are under development (78, 81–84). Furthermore, recent findings identified target neoepitopes which can be helpful in the design of a vaccine against murine melanoma (85). Importantly, the immunogenicity and specificity of these neoepitopes was validated *in vivo*, after administering mice either mutated or wild type synthetic peptides. Further advance in the field was made by Verdegaal et al. who analyzed the stability of neoantigen-specific T-cell responses and the antigens they recognize in melanoma patients treated by adoptive T-cell transfer. This study demonstrated that T cells mediate neoantigen immunoediting, indicating that the therapeutic induction of broad neoantigen-specific T-cell responses should be used to avoid tumor resistance (86).

In comparison to melanoma, the immune CYT in breast cancer, the burden of nonsynonymous mutations, and the predicted load of neoepitopes were previously found to be relatively modest, suggesting that a combination of immune agents with nonredundant mechanisms of action should be of high-priority (87). Recently, Vonderheide et al. highlight the critical steps that need to be followed for a more successful immunotherapy in breast cancer, including immune suppression in the tumor microenvironment and failed or suboptimal T-cell priming (87).

Also Chen et al. (88) categorized the tumor microenvironment into four types, depending on the expression of PD-L1, as well as the ratio CD8A/CYT, and proposed that this classification can serve the design of more suitable immunotherapeutic strategies.

A very interesting improvement in the field was further made by Riaz et al. (89), who showed that the mutation burden in melanoma patients decreases with successful anti-PD-1 blockade therapy, suggesting that the selection against mutant neoepitopes is a critical mechanism of action of this immunotherapy.

All these advances, show that neoepitopes can be used as biomarkers to predict the clinical response to immunotherapy and the outcome, as well as to serve as immunotherapy targets (25, 90). Besides epitope selection, the reduction of gene expression heterogeneity within tumor cells, the definition of the optimum number of simultaneously targeted neoantigens, of the patient profile that can benefit from neoantigen-based immunotherapy and escape the risk of adverse effects, and a synergistic combination of immune-checkpoint blockade and/or adoptive T-cell therapy, are all issues that need to be successfully addressed in order to select potent neoantigens for cancer immunotherapy (77).

Adding to the variability in cytolytic levels that we detected among different cancer types, CYT has also been previously shown to correlate with oncogenic viruses in certain tumor types. For example, CYT is associated with HPV infection in cervical cancer, and head and neck cancer, with EBV infection in stomach cancer, and with HBV and HCV infection in liver cancer (31). Overall, it seems that CYT is part of an inflammatory environment in a premalignant state of certain tumor types, whereas, in others, oncogenic mutations, copy number aberrations, or viral infection can induce a tumor-promoting inflammatory microenvironment, within which complex interactions between different cell types regulate cancer development and metastasis (91, 92).

In the context of metastasis, we observed that CYT was significantly higher in metastatic skin melanoma compared to primary skin tumors. The increased cytolytic levels could be further observed in metastatic breast and thyroid cancers suggesting that although initially regarded as an indicator of a failed immune response, CTLs/NK cells (among other inflammatory cells) also support tumor development (93, 94). This observation is in agreement with previous reports supporting that regardless of the tumor’s origin, an inflamed tumor microenvironment has many tumor-promoting effects (91, 92). In line with this, we found significantly elevated expression of various suppressive factors, correlating with a high cytolytic index, in metastatic skin melanomas, breast, and thyroid cancers. For example, high levels of CTLA-4, PD-1, PD-L1/2, LAG3, and IDO1 that we detected in metastatic melanoma, were many fold times more significantly associated with high cytolytic levels, pointing towards the existence of immunosuppression in these metastatic tumors (Figure S8 in Supplementary Material).

The above-mentioned vast range in the CYT and the different levels of infiltration of inflammatory cells (T cells and neutrophils) is best reflected by the different survival curves produced among different types of cancer. In some tumor types (ACC, SKCM, BLCA, LIHC, MESO, OV, STAD, THCA, and UCEC), high CYT was associated with an improved outcome; whereas in others (LGG, BRCA, THYM, LUAD/LUSC, PAAD, PRAD, and READ) it is correlated with a worse outcome. Among LGG, THYM, and BRCA, we showed that both individual and simultaneous high levels of GZMA and PRF1 were significantly associated with a worse prognosis, whereas the simultaneous low levels of both cytolytic genes led to a significant shift toward a positive effect. Nevertheless, we could not observe this across different breast cancer datasets. Furthermore, contrasting results mentioning a worse effect of PRF1 on survival of BRCA patients were also recently reported in another large-scale meta-analysis (95). The difference between the two studies might be due to cohort-specific bias or power-related discrepancies. Of interest, among certain tumor types including ACC, SKCM and BRCA, the simultaneous expression of both cytolytic genes synergistically affected patient survival.

Tumor-infiltrating lymphocytes are mononuclear cells of the immune system that intrude the tumor tissue, and their presence has been reported in solid tumors, such as breast, colon, lung, and cervical cancers, as well as in melanoma (43, 96–98). Low levels of CD8+ TILs are related with the likelihood of response and may escalate during therapy in responding tumors (2, 99). Further, the location of CD8+ TILs at the invasive margin of tumors may indicate an effective immune response (42, 99, 100). The tumor microenvironment may limit extravasation of effector T cells into the tumor, diminish their expansion, or reduce their viability (101). In BRCA, an increased TIL load in the stroma of the tumor was reported to associate with a higher prospect of therapy in early stage TNBC and Her2+ patients (98). Assessing light microscopy data of tissue slides, we found a higher TIL load in primary BRCA compared to the metastatic counterparts, but the differences were not significant (*p* > 0.05). These lymphocytic infiltrates mirror favorable host antitumor immune responses within these samples. Although the presence of high TIL levels has been previously linked with a more favorable prognosis in patients with Her2+ and early stage TNBC (46–48), we found no significant difference in the outcome of TNBC or TPBC between high and low TIL load.

Tumor-associated neutrophils also compose a significant part of the inflammatory cell infiltrate in several tumor types (102–105), but the mechanisms by which they affect tumor progression are only now being investigated. Recent studies point toward the tumor-promoting effects of neutrophils. Histologic studies performed on a variety of tumor types have shown that the increased TAN load correlates with unfavorable recurrence-free, cancer-specific and overall patient survival in kidney cancer, skin melanoma, colorectal cancer, and head and neck cancer (106). It has also been suggested that TANs can drive the metastasis of breast cancer cells to the liver or the lung (107, 108), activating angiogenesis (109, 110). In contrast, older reports suggested that neutrophils have antitumoral effects, by inducing direct cytotoxicity of target cells, and decreasing the size and the number of lung metastatic foci (111–113). Interestingly, the anticancer activity of TANs was reported to mostly culminate into anticancer activity *via* oxidative burst (114, 115). Despite the heavily debated role in favor or against cancer, the latest research shows that TANs do play a key role in various aspects of tumor development, from malignant transformation to tumor progression, modification of the extracellular matrix, angiogenesis, cell migration, and immunosuppression (116–121). Due to their contradictory roles in cancer, neutrophils are now classified into two subpopulations, antitumor and pro-tumor TANs (117). We detected very low percentage of TANs in primary breast and thyroid carcinomas and almost null levels in their metastatic counterparts. We noticed higher neutrophilic infiltration in TNBC compared to TPBC, but without reaching statistical significance (*p* > 005). In skin melanoma, we noticed even less neutrophilic infiltration, being slightly higher in the metastatic tumors.

Overall, the multiple crosstalks among different tumor-infiltrating immune cells, including TILs and TANs, was suggested to promote or inhibit the establishment of a permissive tumor microenvironment (17). A better understanding of the role of these cells will provide opportunities for the immunomodulation and the improvement of the existing antitumor therapies.

To conclude, we have measured the CYT in terms of RNA and protein levels in a large number of TCGA datasets, in order to understand how different cancers induce and adapt to immune responses. We associated each cancer’s CYT with patient survival both in primary and metastatic cases and evaluated the tumor-infiltration of lymphocytes and neutrophils in H&E-stained sections of the same tumors. Our data suggest that the cytolytic index along with the existence of complicated associations among various tumor-infiltrated immune cells is capable to promote evasion from immunosurveillance in certain cancers.

## Author Contributions

CR extracted data, analyzed them, and performed statistical computations. DC, AM, and CE extracted data. CD critically read the manuscript and approved its final version. AZ supervised the study, extracted, and analyzed data, interpreted the results, and wrote the manuscript.

## Conflict of Interest Statement

The authors declare that the research was conducted in the absence of any commercial or financial relationships that could be construed as a potential conflict of interest.

## Footnotes

^1^https://cancergenome.nih.gov/.

^2^https://www.proteinatlas.org/.

^3^https://portal.gdc.cancer.gov/.

^4^https://www.gtexportal.org/home/.

^5^http://fantom.gsc.riken.jp/5/.

^6^http://cancer.digitalslidearchive.net/.
