# Supplementary material for: The Expression and Prognostic Impact of Immune Cytolytic Activity-Related Markers in Human Malignancies: A Comprehensive Meta-analysis
Source: Front Oncol. 2018 Feb 21;8:27. doi: 10.3389/fonc.2018.00027 (PMC5826382; doi:10.3389/fonc.2018.00027)
Supplement: Supplementary file 1 [file image_1.PDF]

Supplementary Material

Title: The expression and prognostic impact of immune cytolytic activity-related markers in human malignancies: A comprehensive meta-analysis

Constantinos Roufas [1,2], Dimitrios Chasiotis [1], Anestis Makris [1], Christodoulos Efstathiades [2], Christos Dimopoulos [2], Apostolos Zaravinos [1,*]

[1] Department of Life Sciences, Biomedical Sciences Program, School of Sciences, European University Cyprus, Nicosia, Cyprus.

[2] The Center for Risk and Decision Sciences (CERIDES), Department of Computer Sciences, School of Sciences, European University Cyprus, Nicosia, Cyprus.

*** Correspondence: Apostolos Zaravinos, PhD. Biomedical Sciences Program, Department of Life Sciences, School of Sciences, European University Cyprus. 6, Diogenes Str. Engomi, P.O. Box 22006, 1516, Nicosia, Cyprus. Tel: +357-22559577. Email: a.zaravinos@euc.ac.cy**

Supplementary Figures

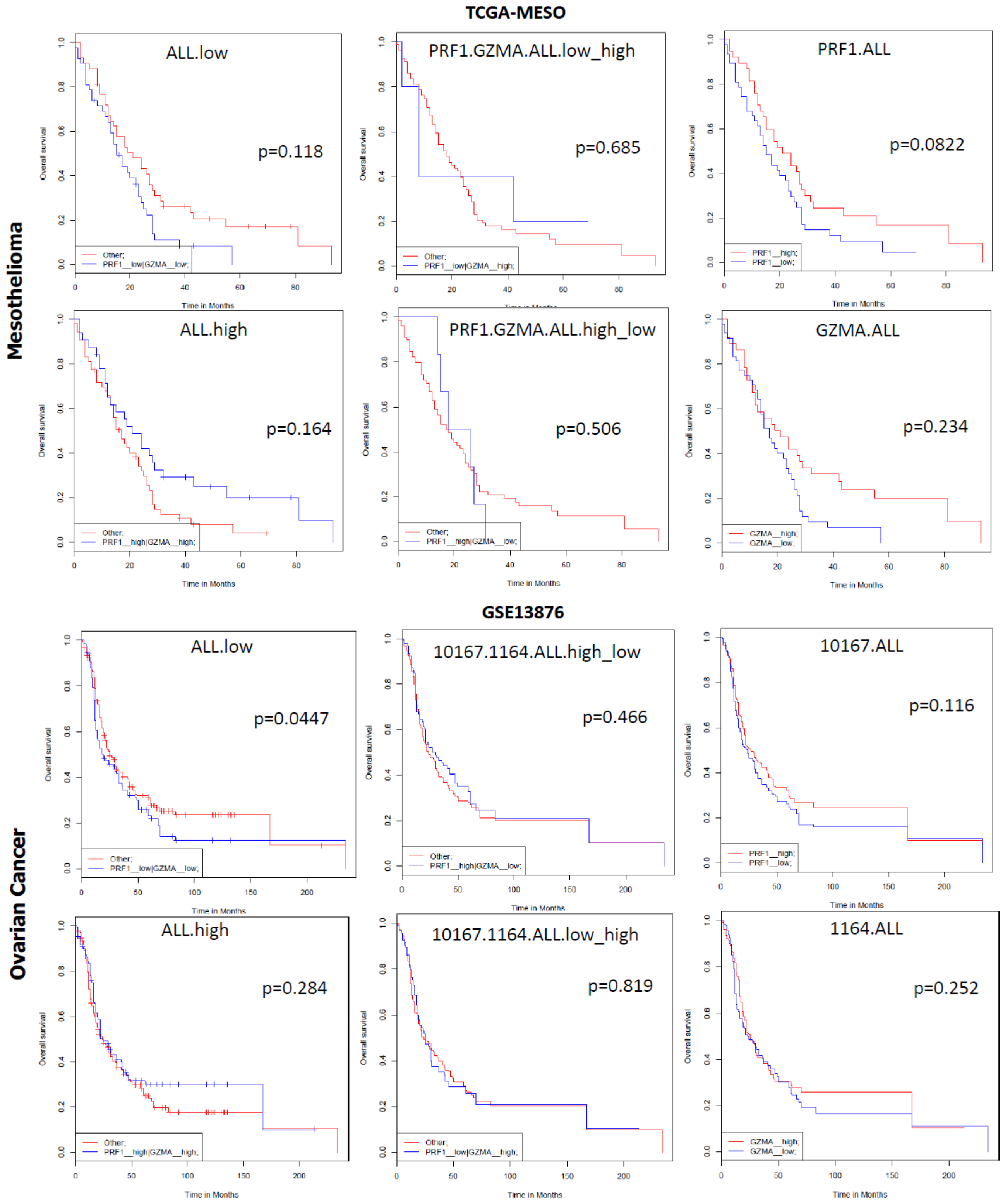

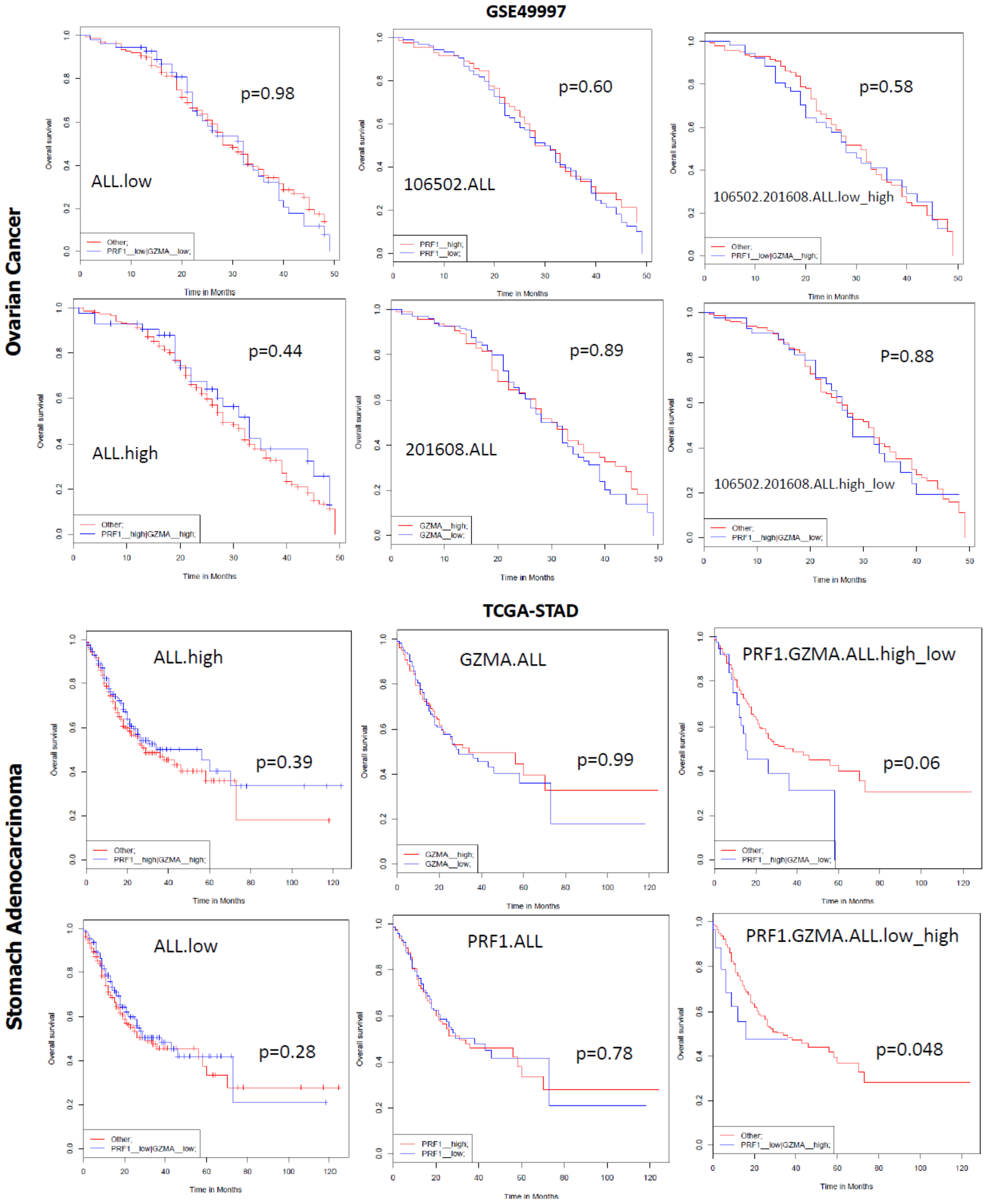
GSE49997
Ovarian Cancer
p=0.98
ALL.low
Other;
PRF1__low|GZMA__low;
p=0.60
106502.ALL
PRF1__high;
PRF1__low;
p=0.58
106502.201608.ALL.low_high
Other;
PRF1__low|GZMA__high;
p=0.44
ALL.high
Other;
PRF1__high|GZMA__high;
p=0.89
201608.ALL
GZMA__high;
GZMA__low;
P=0.88
106502.201608.ALL.high_low
Other;
PRF1__high|GZMA__low;
TCGA-STAD
Stomach Adenocarcinoma
ALL.high
p=0.39
Other;
PRF1__high|GZMA__high;
GZMA.ALL
p=0.99
GZMA__high;
GZMA__low;
PRF1.GZMA.ALL.high_low
p=0.06
Other;
PRF1__high|GZMA__low;
ALL.low
p=0.28
Other;
PRF1__low|GZMA__low;
PRF1.ALL
p=0.78
PRF1__high;
PRF1__low;
PRF1.GZMA.ALL.low_high
p=0.048
Other;
PRF1__low|GZMA__high;
Overall survival
Time in Months

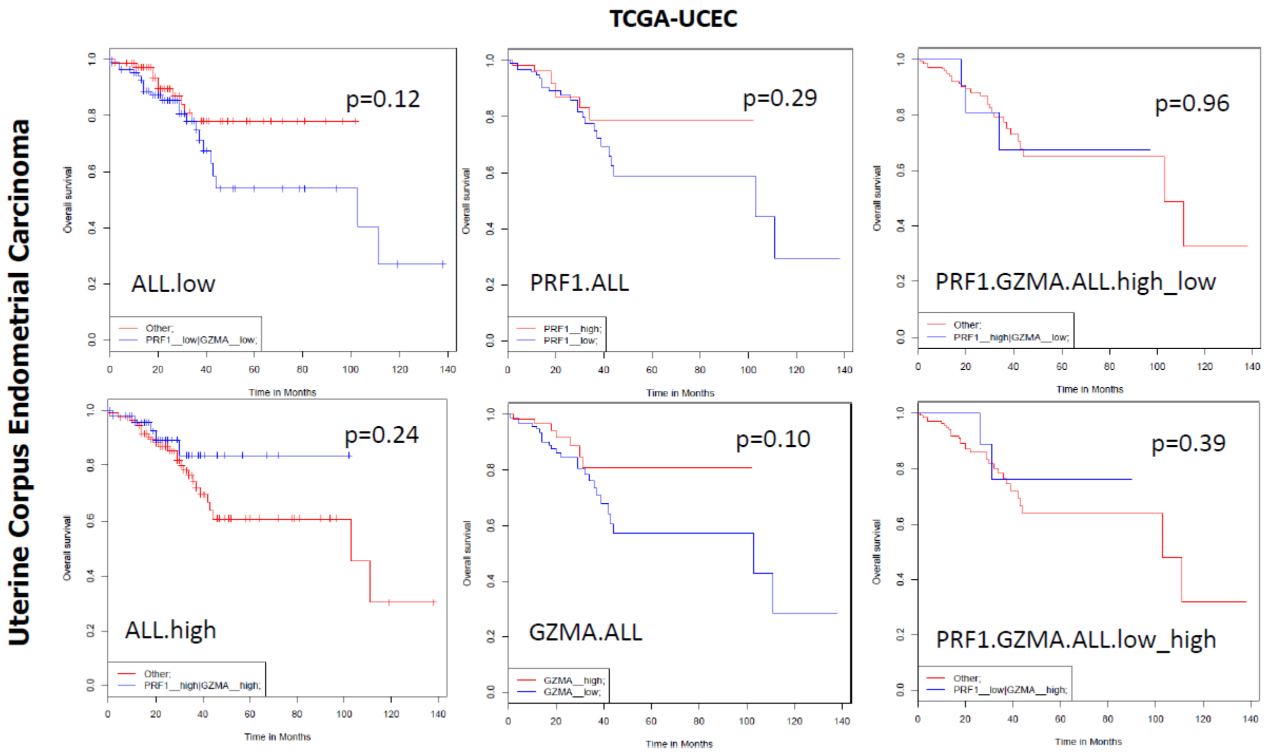

Figure S1. In liver hepatocellular carcinoma (TCGA-LIHC) only the individual high levels of PRF1 and GZMA were significantly associated with a positive effect on patient survival. A similar non-significant association of (individual or simultaneous) high GZMA and PRF1 expression with better effect on patient survival could also be observed in mesothelioma (TCGA-MESO), ovarian cancer (GSE13876 and GSE49997), stomach adenocarcinoma (TCGA-STAD), thyroid carcinoma (TCGA-THCA) and uterine corpus endometrial carcinoma (TCGA-UCEC).
