# Supplementary material for: The Expression and Prognostic Impact of Immune Cytolytic Activity-Related Markers in Human Malignancies: A Comprehensive Meta-analysis
Source: Front Oncol. 2018 Feb 21;8:27. doi: 10.3389/fonc.2018.00027 (PMC5826382; doi:10.3389/fonc.2018.00027)
Supplement: Supplementary file 2 [file image_2.PDF]

frontiers

Supplementary Material

Title: The expression and prognostic impact of immune cytolytic activity-related markers in human malignancies: A comprehensive meta-analysis

Constantinos Roufas [1,2], Dimitrios Chasiotis [1], Anestis Makris [1], Christodoulos Efstathiades [2], Christos Dimopoulos [2], Apostolos Zaravinos [1,*]

[1] Department of Life Sciences, Biomedical Sciences Program, School of Sciences, European University Cyprus, Nicosia, Cyprus.

[2] The Center for Risk and Decision Sciences (CERIDES), Department of Computer Sciences, School of Sciences, European University Cyprus, Nicosia, Cyprus.

*** Correspondence: Apostolos Zaravinos, PhD. Biomedical Sciences Program, Department of Life Sciences, School of Sciences, European University Cyprus. 6, Diogenes Str. Engomi, P.O. Box 22006, 1516, Nicosia, Cyprus. Tel: +357-22559577. Email: a.zaravinos@euc.ac.cy**

Supplementary Figures

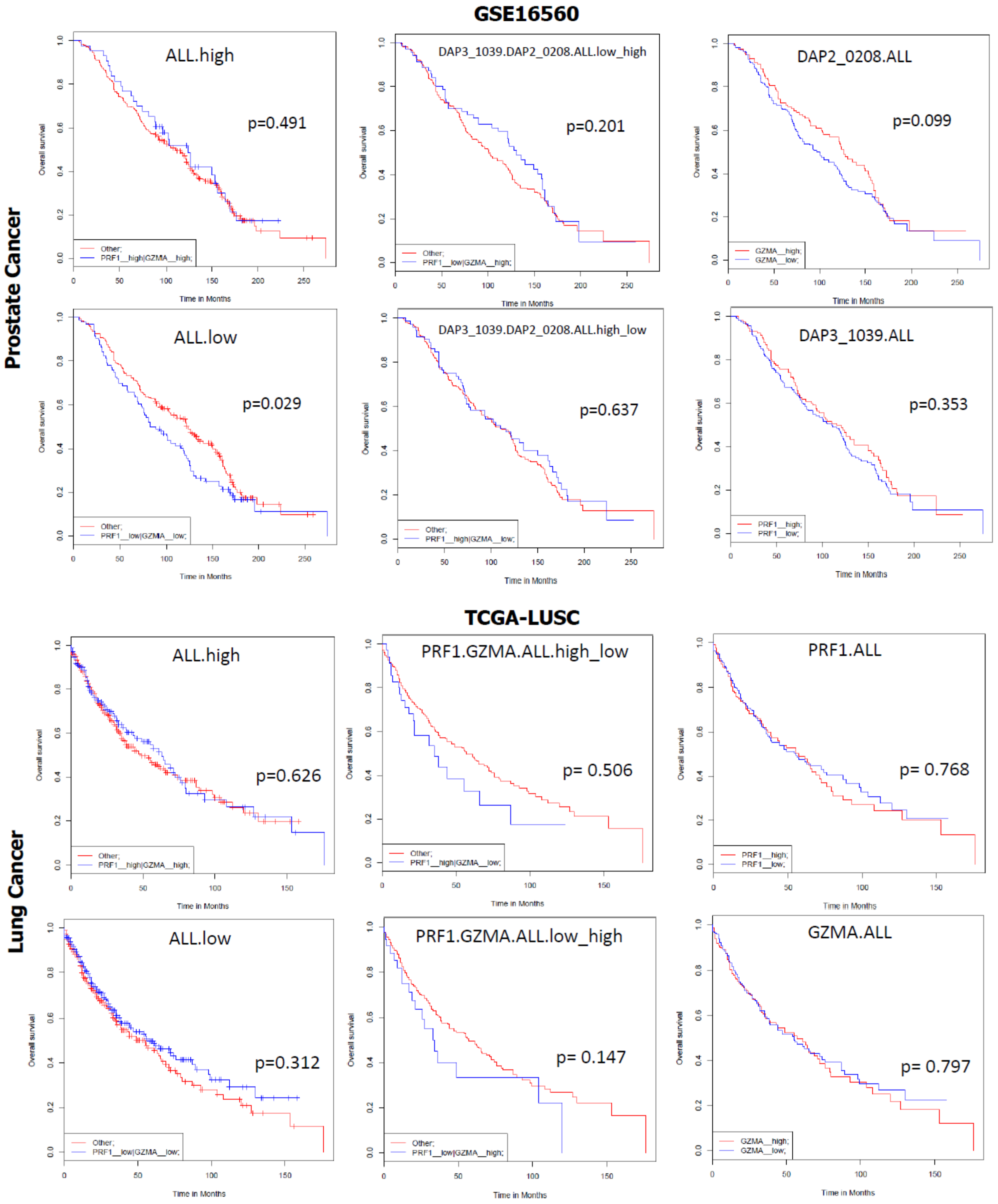

TCGA-PAAD

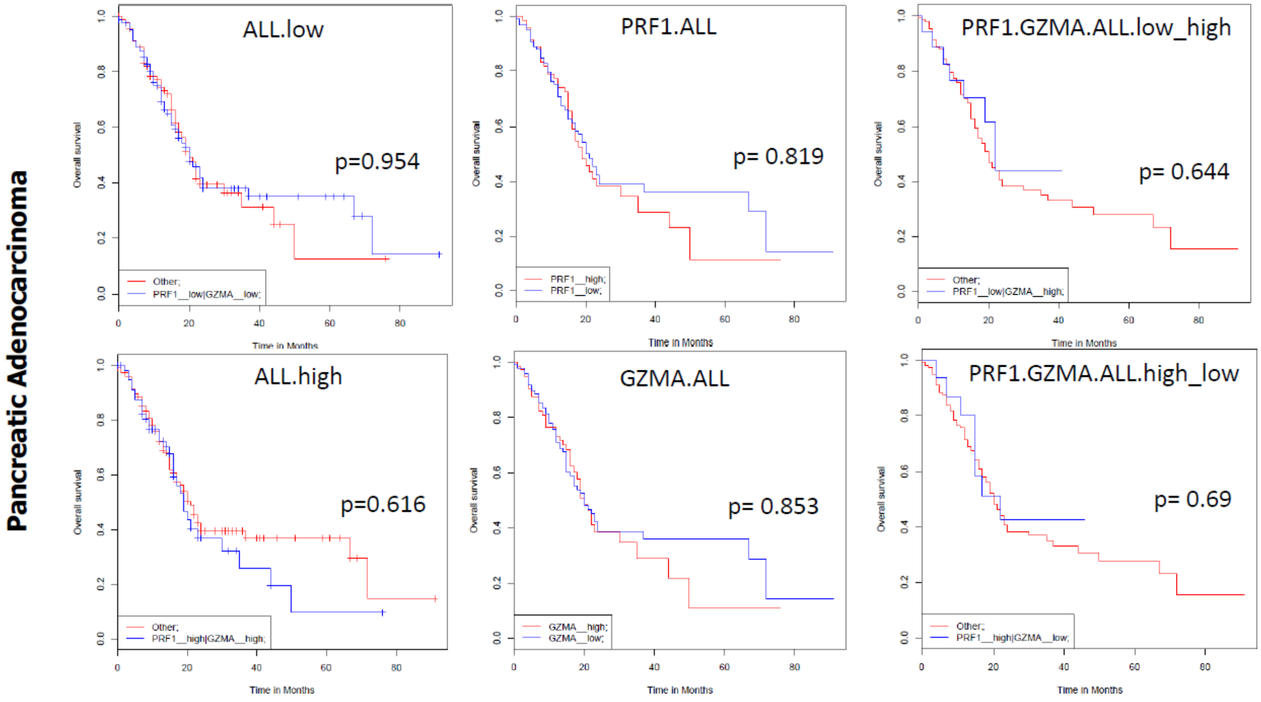

TCGA-PRAD

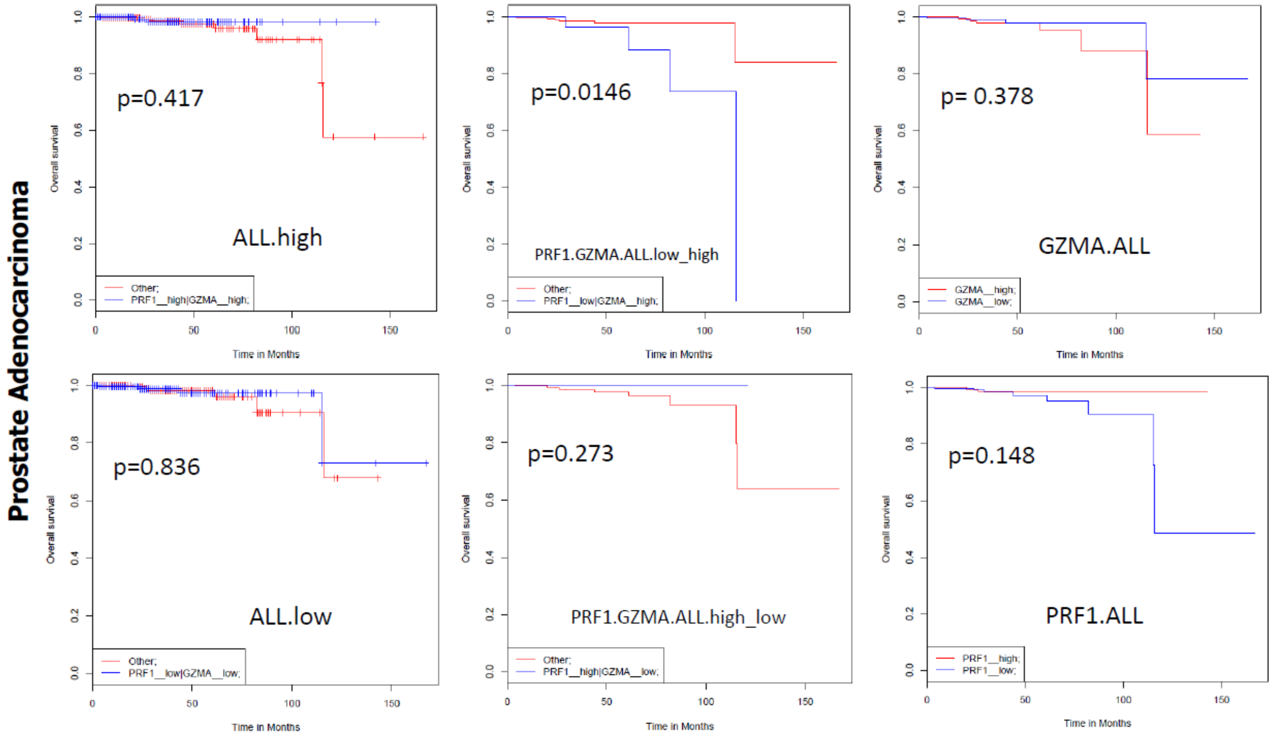

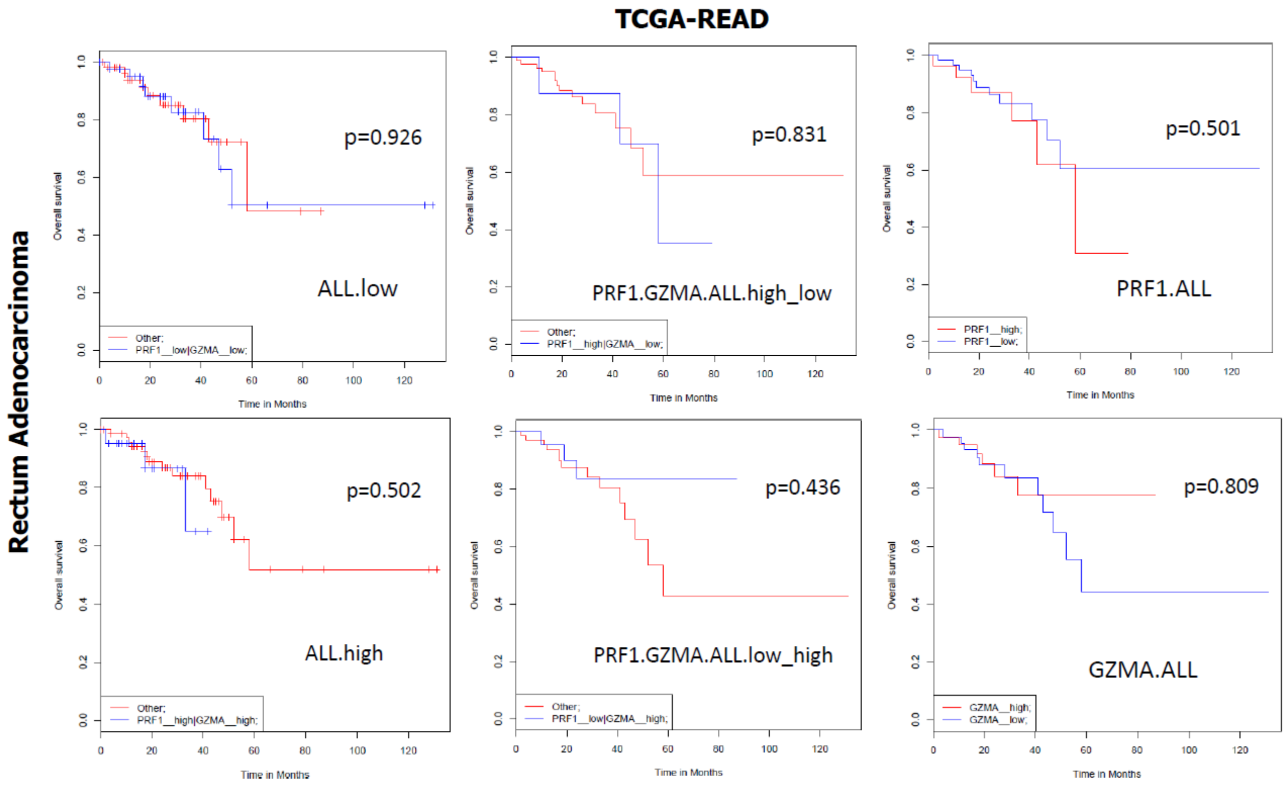
TCGA-READ
Rectum Adenocarcinoma
p=0.926
ALL.low
Other;
PRF1__low|GZMA__low;
p=0.831
PRF1.GZMA.ALL.high_low
Other;
PRF1__high|GZMA__low;
p=0.501
PRF1.ALL
PRF1__high;
PRF1__low;
p=0.502
ALL.high
Other;
PRF1__high|GZMA__high;
p=0.436
PRF1.GZMA.ALL.low_high
Other;
PRF1__low|GZMA__high;
p=0.809
GZMA.ALL
GZMA__high;
GZMA__low;
Overall survival
Time in Months

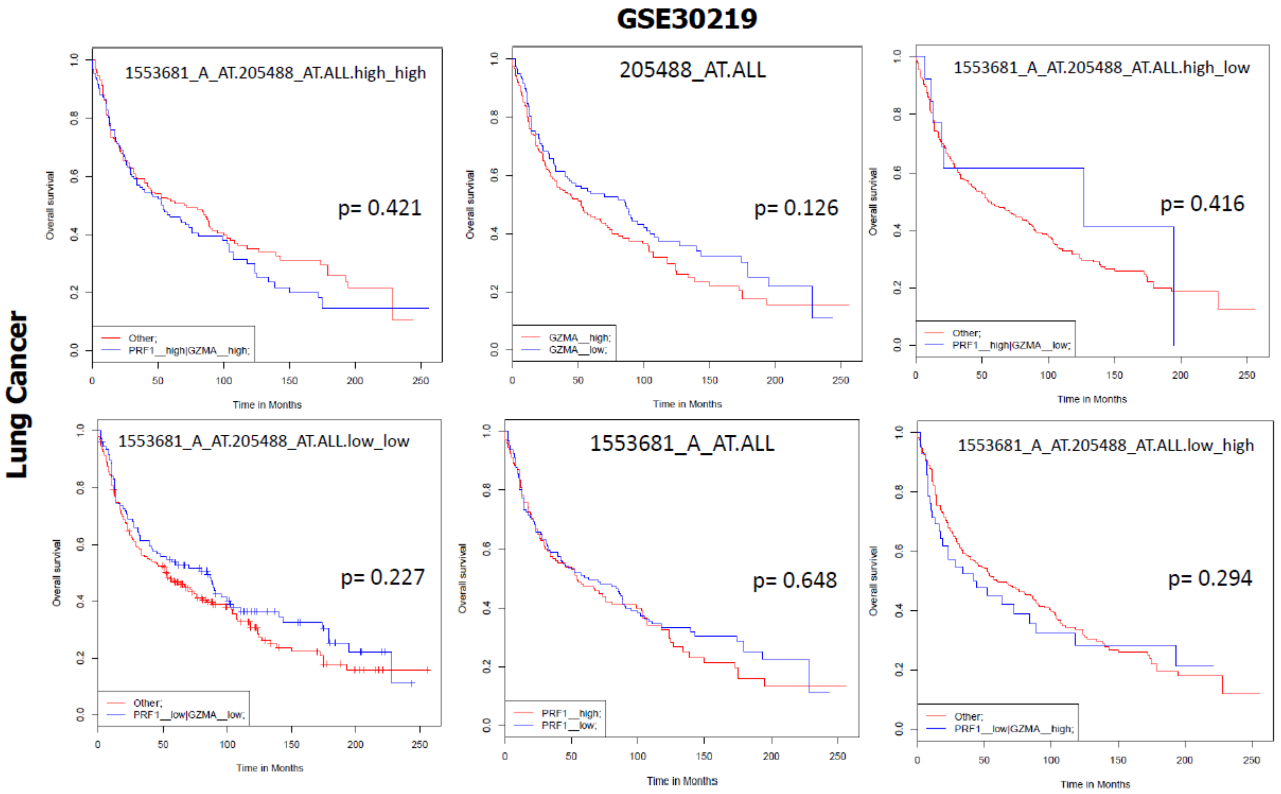
GSE30219
Lung Cancer
1553681_A_AT.205488_AT.ALL.high_high
p= 0.421
Other;
PRF1__high|GZMA__high;
205488_AT.ALL
p= 0.126
GZMA__high;
GZMA__low;
1553681_A_AT.205488_AT.ALL.high_low
p= 0.416
Other;
PRF1__high|GZMA__low;
1553681_A_AT.205488_AT.ALL.low_low
p= 0.227
Other;
PRF1__low|GZMA__low;
1553681_A_AT.ALL
p= 0.648
PRF1__high;
PRF1__low;
1553681_A_AT.205488_AT.ALL.low_high
p= 0.294
Other;
PRF1__low|GZMA__high;
Overall survival
Time in Months

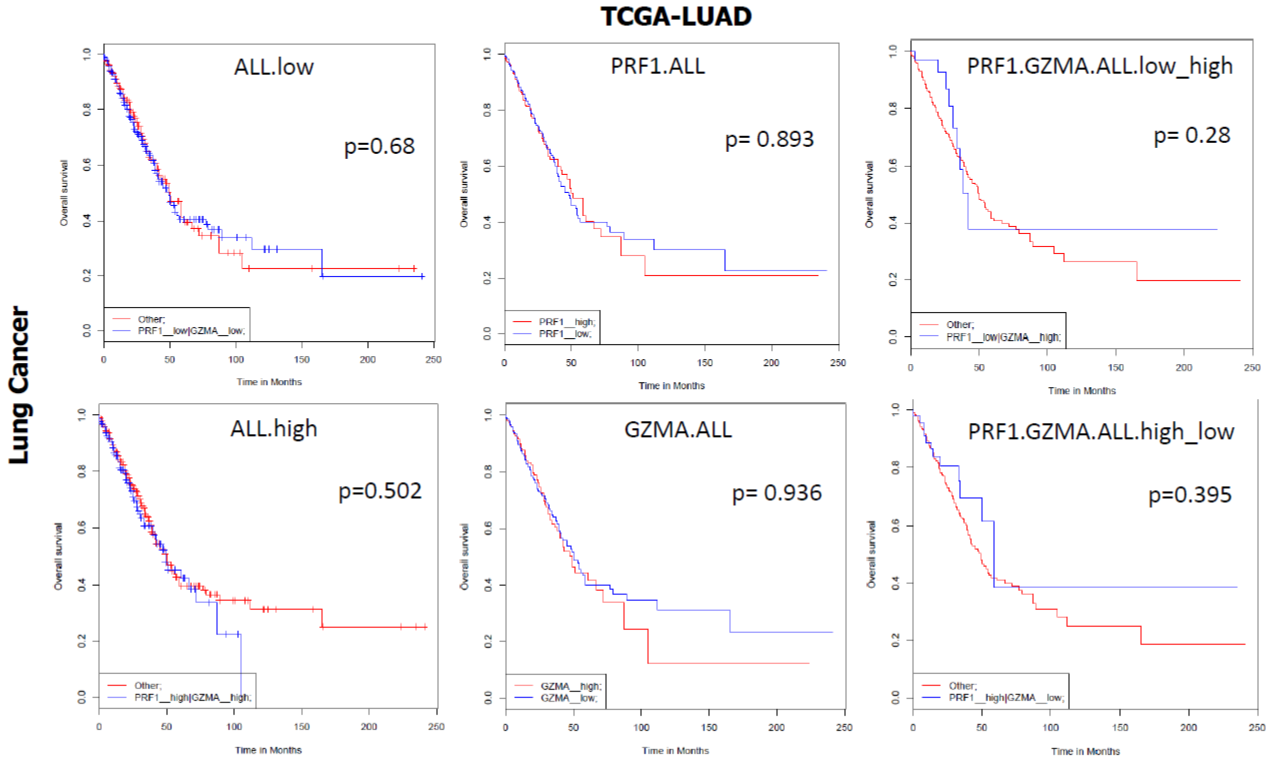

Figure S2. Non-significant associations of (individual or simultaneous) high CYT levels with worse effect on patient survival were observed in lung cancer (GSE30219, TCGA-LUAD and TCGA-LUSC), pancreatic adenocarcinoma (TCGA-PAAD), prostate adenocarcinoma (TCGA-PRAD and GSE16560), and rectum adenocarcinoma (TCGA-READ).
