# Supplementary material for: The Expression and Prognostic Impact of Immune Cytolytic Activity-Related Markers in Human Malignancies: A Comprehensive Meta-analysis
Source: Front Oncol. 2018 Feb 21;8:27. doi: 10.3389/fonc.2018.00027 (PMC5826382; doi:10.3389/fonc.2018.00027)
Supplement: Supplementary file 3 [file image_3.PDF]

frontiers

Supplementary Material

Title: The expression and prognostic impact of immune cytolytic activity-related markers in human malignancies: A comprehensive meta-analysis

Constantinos Roufas [1,2], Dimitrios Chasiotis [1], Anestis Makris [1], Christodoulos Efstathiades [2], Christos Dimopoulos [2], Apostolos Zaravinos [1,*]

[1] Department of Life Sciences, Biomedical Sciences Program, School of Sciences, European University Cyprus, Nicosia, Cyprus.

[2] The Center for Risk and Decision Sciences (CERIDES), Department of Computer Sciences, School of Sciences, European University Cyprus, Nicosia, Cyprus.

*** Correspondence: Apostolos Zaravinos, PhD. Biomedical Sciences Program, Department of Life Sciences, School of Sciences, European University Cyprus. 6, Diogenes Str. Engomi, P.O. Box 22006, 1516, Nicosia, Cyprus. Tel: +357-22559577. Email: a.zaravinos@euc.ac.cy**

Supplementary Figures

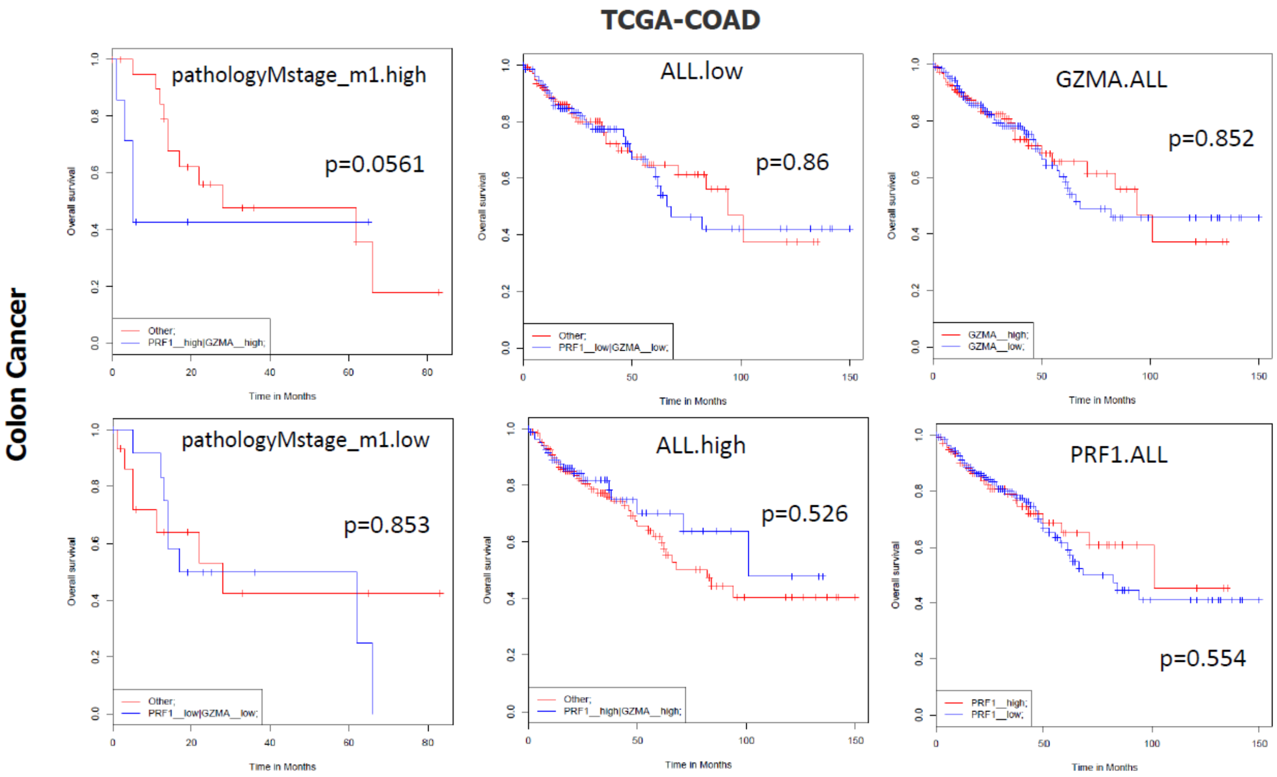

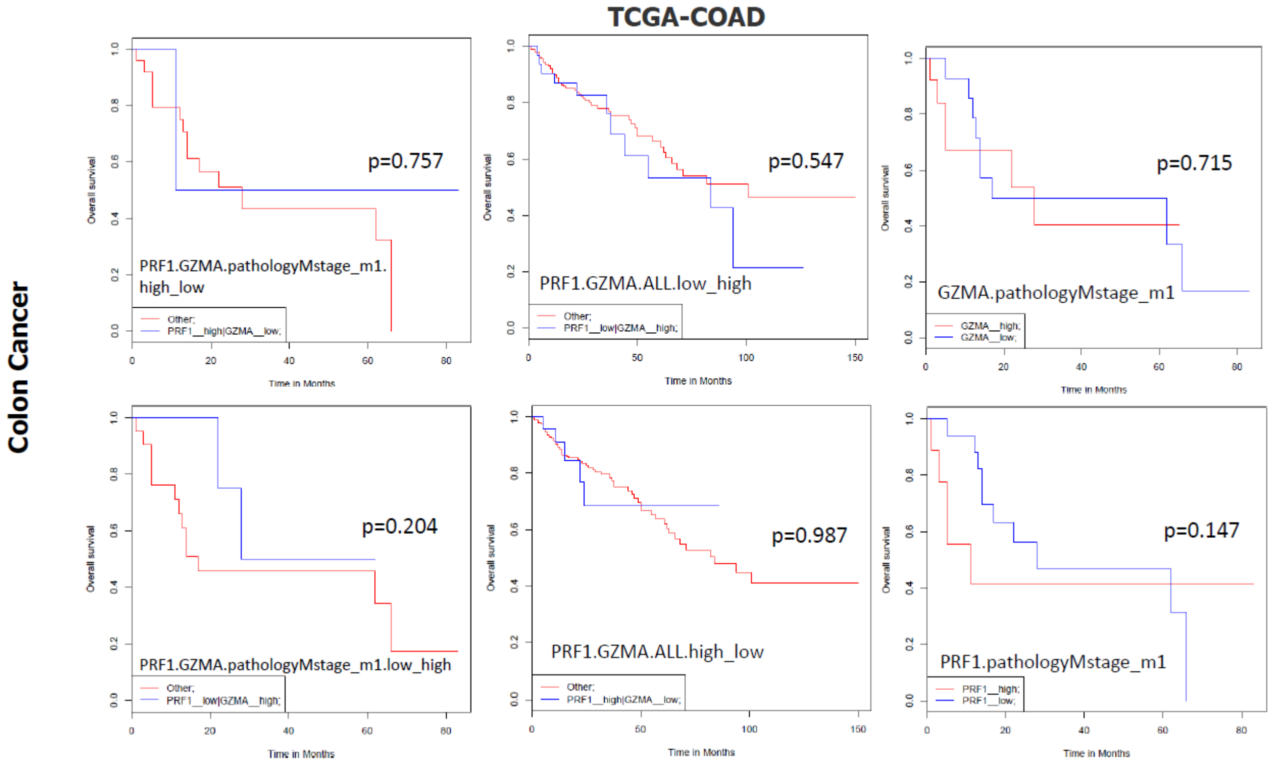

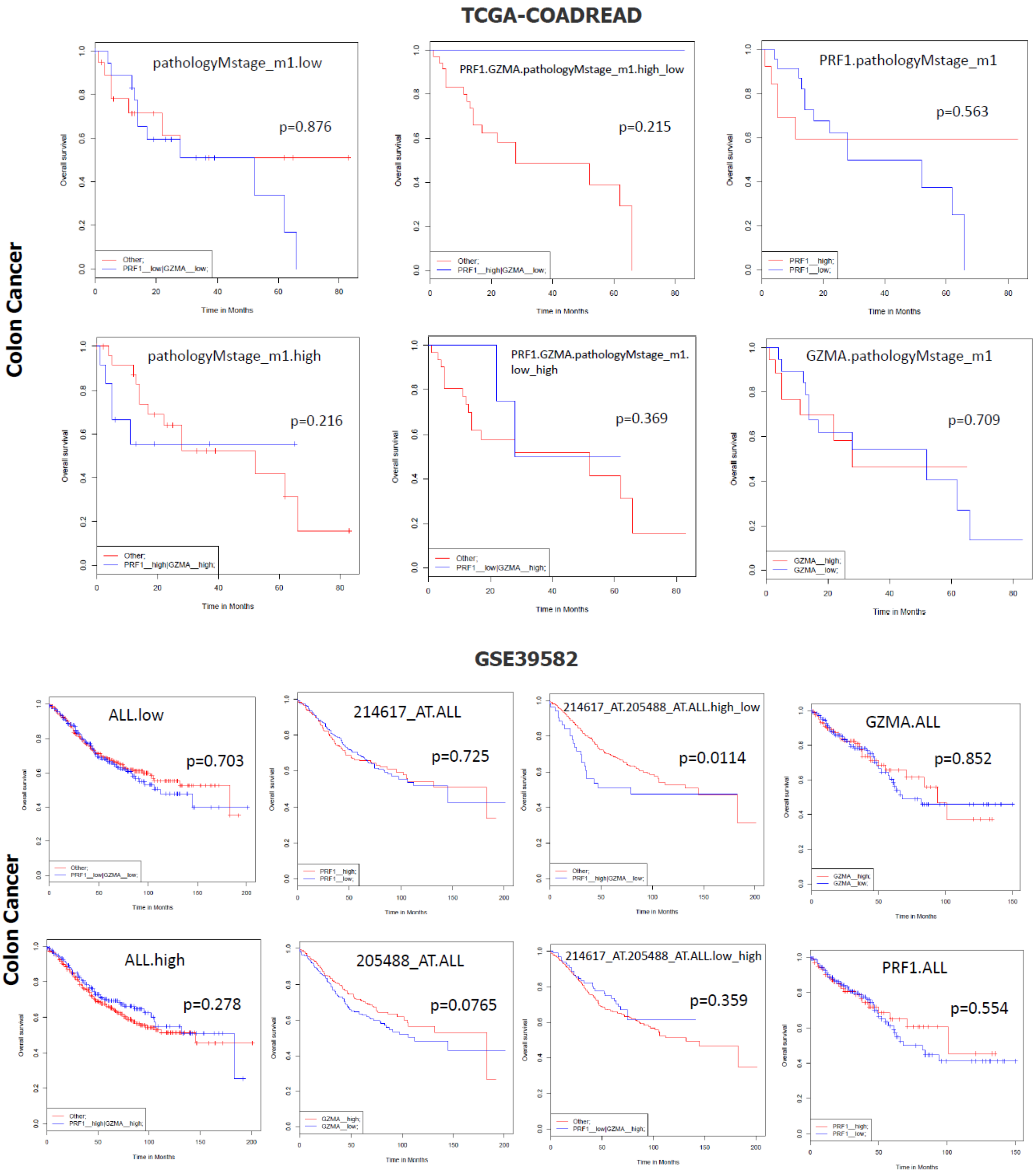
TCGA-COADREAD
Colon Cancer
pathologyMstage_m1.low
p=0.876
PRF1.GZMA.pathologyMstage_m1.high_low
p=0.215
PRF1.pathologyMstage_m1
p=0.563
pathologyMstage_m1.high
p=0.216
PRF1.GZMA.pathologyMstage_m1.low_high
p=0.369
GZMA.pathologyMstage_m1
p=0.709
GSE39582
Colon Cancer
ALL.low
p=0.703
214617_AT.ALL
p=0.725
214617_AT.205488_AT.ALL.high_low
p=0.0114
GZMA.ALL
p=0.852
ALL.high
p=0.278
205488_AT.ALL
p=0.0765
214617_AT.205488_AT.ALL.low_high
p=0.359
PRF1.ALL
p=0.554
Overall survival
Time in Months

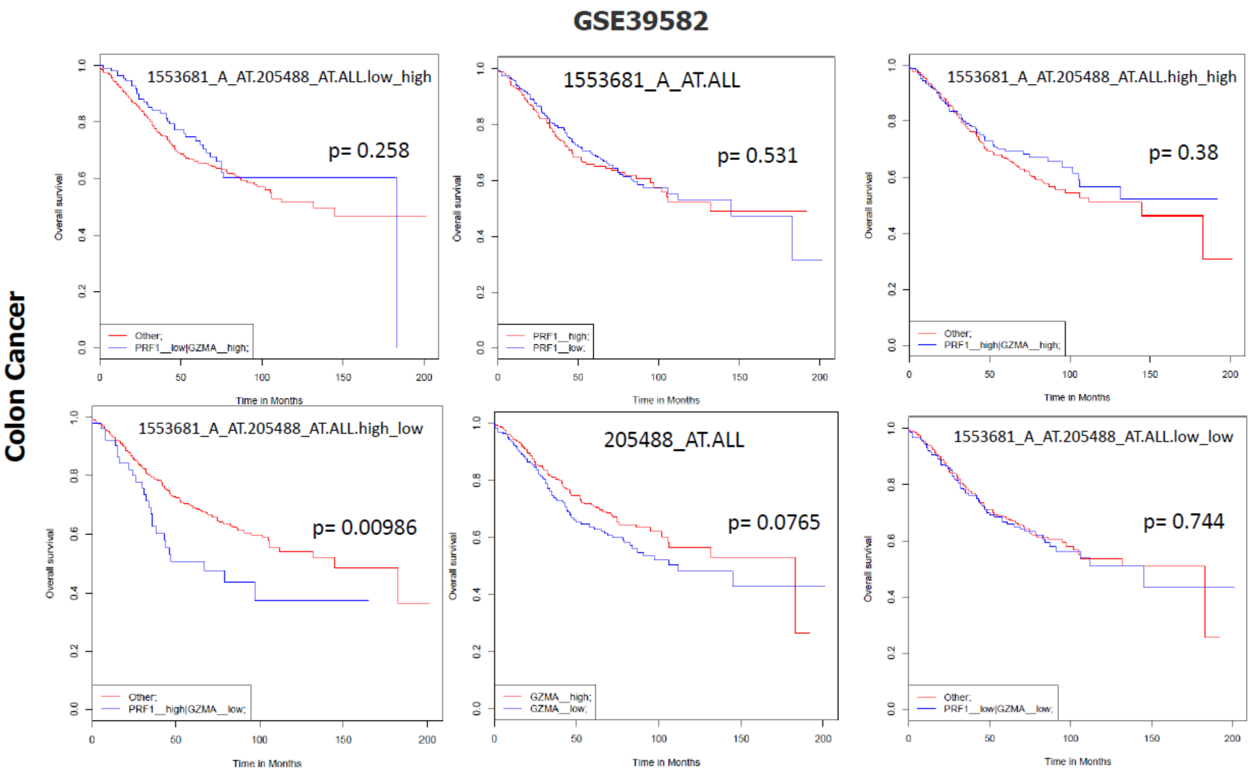

Figure S3. In colorectal cancer, neither individual nor simultaneous high levels of the two cytolytic genes were associated with a better prognosis, although simultaneous low levels of GZMA and PRF1 tended to shift towards a negative effect. Depending on the probe used, it seemed that a combination of high PRF1 levels and low GZMA levels yields a better patient outcome (GSE39582, TCGA-COAD, TCGA-COADREAD). Among metastatic colon cancer patients ("M1" patients in the TCGA-COAD dataset), simultaneous high levels of both genes was marginally significantly associated with worse prognosis, but simultaneous low levels of both genes could not provide the reverse trend.
