# Supplementary material for: The Expression and Prognostic Impact of Immune Cytolytic Activity-Related Markers in Human Malignancies: A Comprehensive Meta-analysis
Source: Front Oncol. 2018 Feb 21;8:27. doi: 10.3389/fonc.2018.00027 (PMC5826382; doi:10.3389/fonc.2018.00027)
Supplement: Supplementary file 4 [file image_4.PDF]

Supplementary Material

Title: The expression and prognostic impact of immune cytolytic activity-related markers in human malignancies: A comprehensive meta-analysis

Constantinos Roufas [1,2], Dimitrios Chasiotis [1], Anestis Makris [1], Christodoulos Efstathiades [2], Christos Dimopoulos [2], Apostolos Zaravinos [1,*]

[1] Department of Life Sciences, Biomedical Sciences Program, School of Sciences, European University Cyprus, Nicosia, Cyprus.

[2] The Center for Risk and Decision Sciences (CERIDES), Department of Computer Sciences, School of Sciences, European University Cyprus, Nicosia, Cyprus.

*** Correspondence: Apostolos Zaravinos, PhD. Biomedical Sciences Program, Department of Life Sciences, School of Sciences, European University Cyprus. 6, Diogenes Str. Engomi, P.O. Box 22006, 1516, Nicosia, Cyprus. Tel: +357-22559577. Email: a.zaravinos@euc.ac.cy**

Supplementary Figures

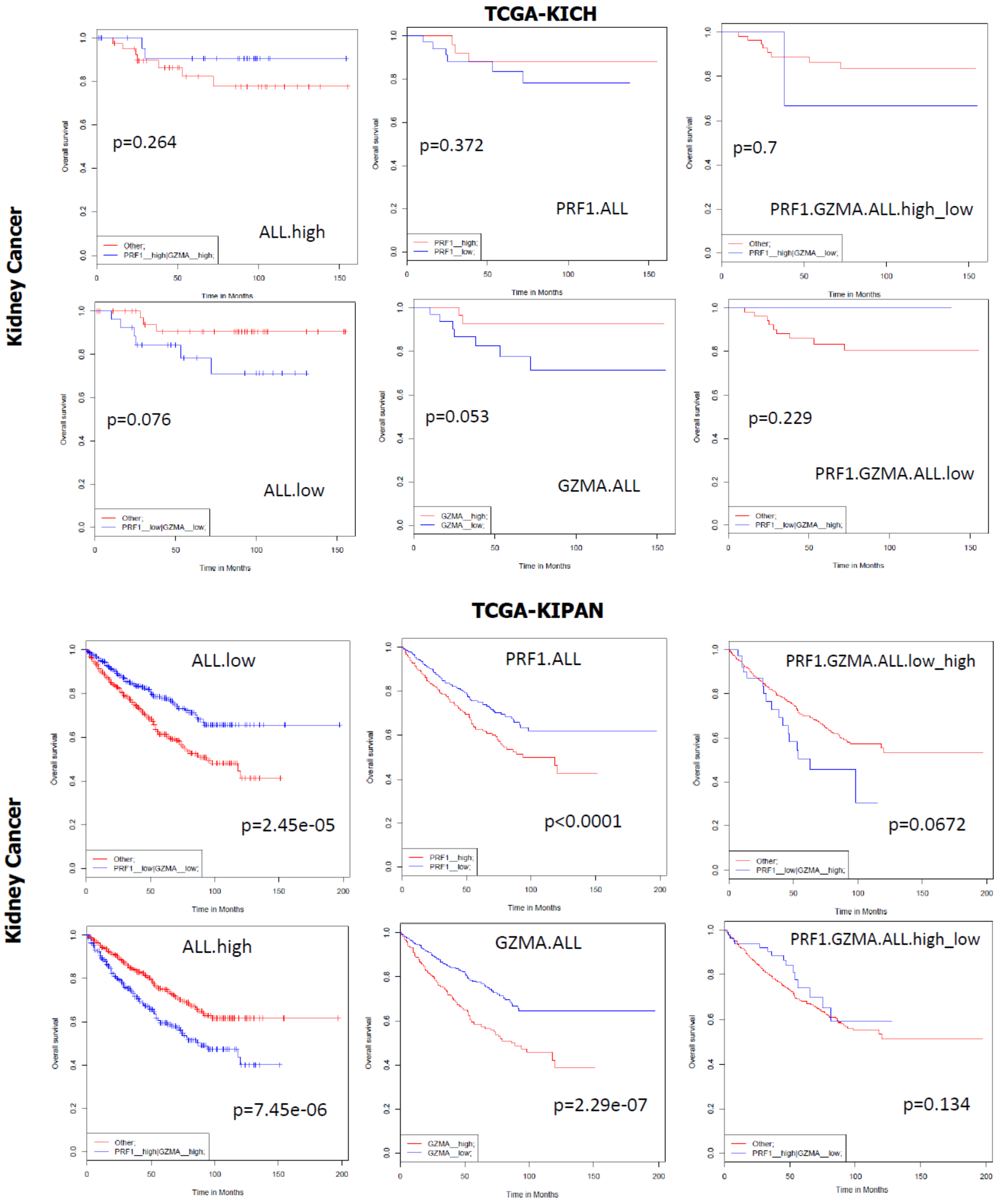

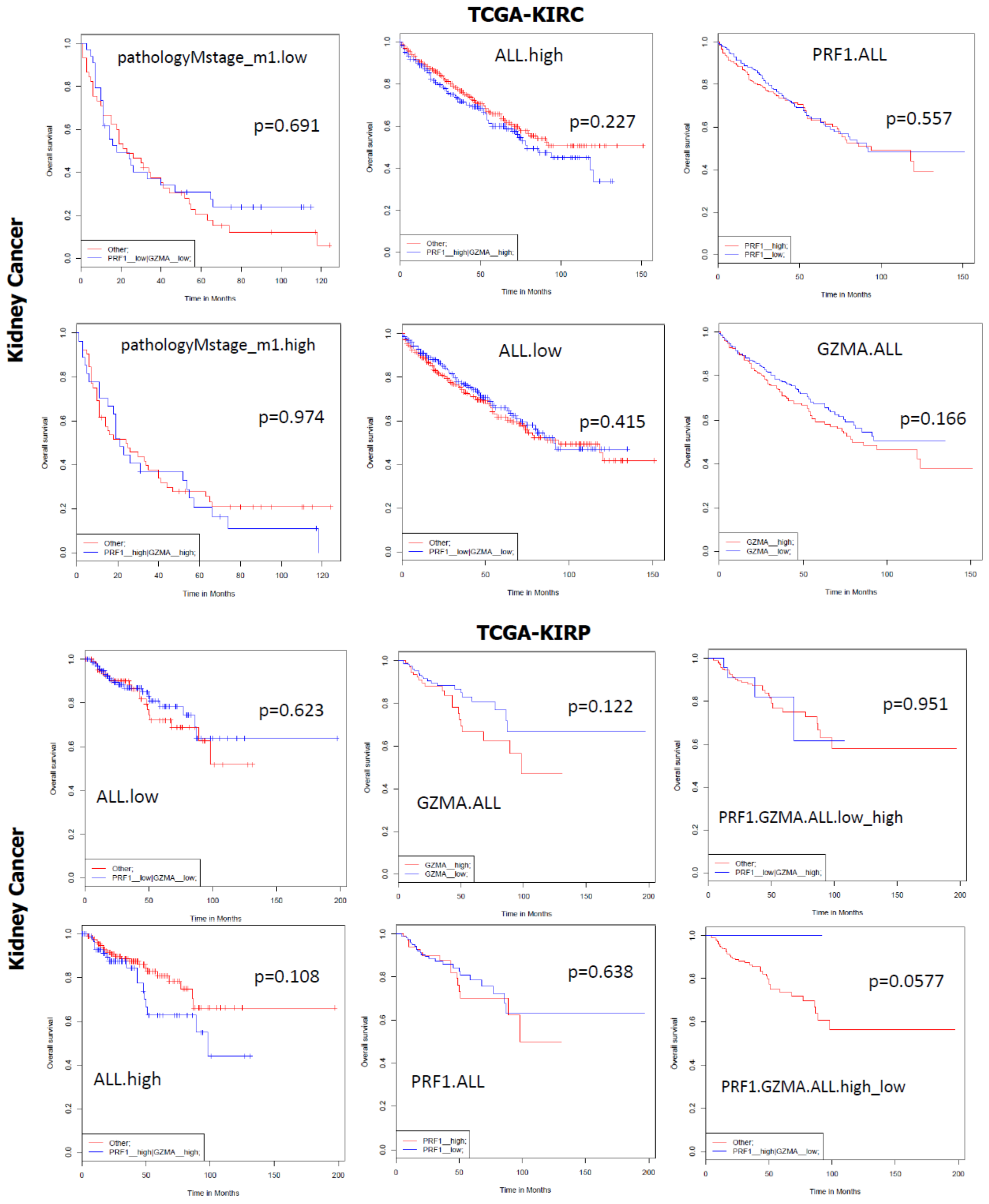

Figure S4. Among clear-cell (TCGA-KIRC) and papillary renal cell carcinomas (TCGA-KIRP), we could not deduce any similar association between metastatic or non-metastatic tumors. In chromophobe renal carcinoma (TCGA-KICH) though, individual and simultaneous high levels of both cytolytic genes tended to associate with better patient survival. On the other hand, concurrent

low GZMA and PRF1 levels tended to associate with worse prognosis. Interestingly, in the TCGA-KIPAN kidney cancer dataset, both the individual and synchronized high levels of GZMA and PRF1 significantly connected with worse patient survival. The simultaneous low expression of both genes exhibited the reverse outcome.
