# Supplementary material for: The Expression and Prognostic Impact of Immune Cytolytic Activity-Related Markers in Human Malignancies: A Comprehensive Meta-analysis
Source: Front Oncol. 2018 Feb 21;8:27. doi: 10.3389/fonc.2018.00027 (PMC5826382; doi:10.3389/fonc.2018.00027)
Supplement: Supplementary file 5 [file image_5.PDF]

frontiers

Supplementary Material

Title: The expression and prognostic impact of immune cytolytic activity-related markers in human malignancies: A comprehensive meta-analysis

Constantinos Roufas [1,2], Dimitrios Chasiotis [1], Anestis Makris [1], Christodoulos Efstathiades [2], Christos Dimopoulos [2], Apostolos Zaravinos [1,*]

[1] Department of Life Sciences, Biomedical Sciences Program, School of Sciences, European University Cyprus, Nicosia, Cyprus.

[2] The Center for Risk and Decision Sciences (CERIDES), Department of Computer Sciences, School of Sciences, European University Cyprus, Nicosia, Cyprus.

*** Correspondence: Apostolos Zaravinos, PhD. Biomedical Sciences Program, Department of Life Sciences, School of Sciences, European University Cyprus. 6, Diogenes Str. Engomi, P.O. Box 22006, 1516, Nicosia, Cyprus. Tel: +357-22559577. Email: a.zaravinos@euc.ac.cy**

Supplementary Figures

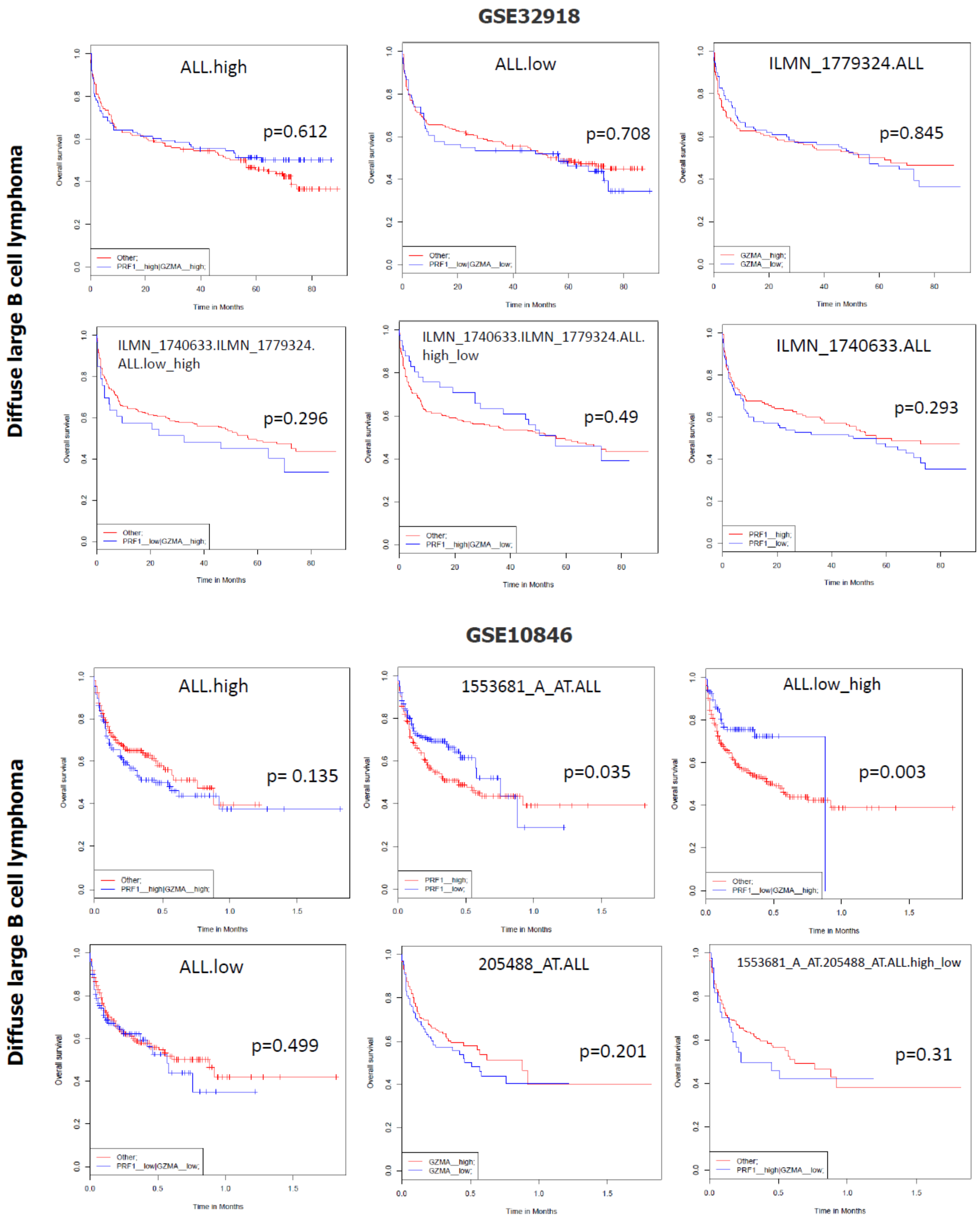

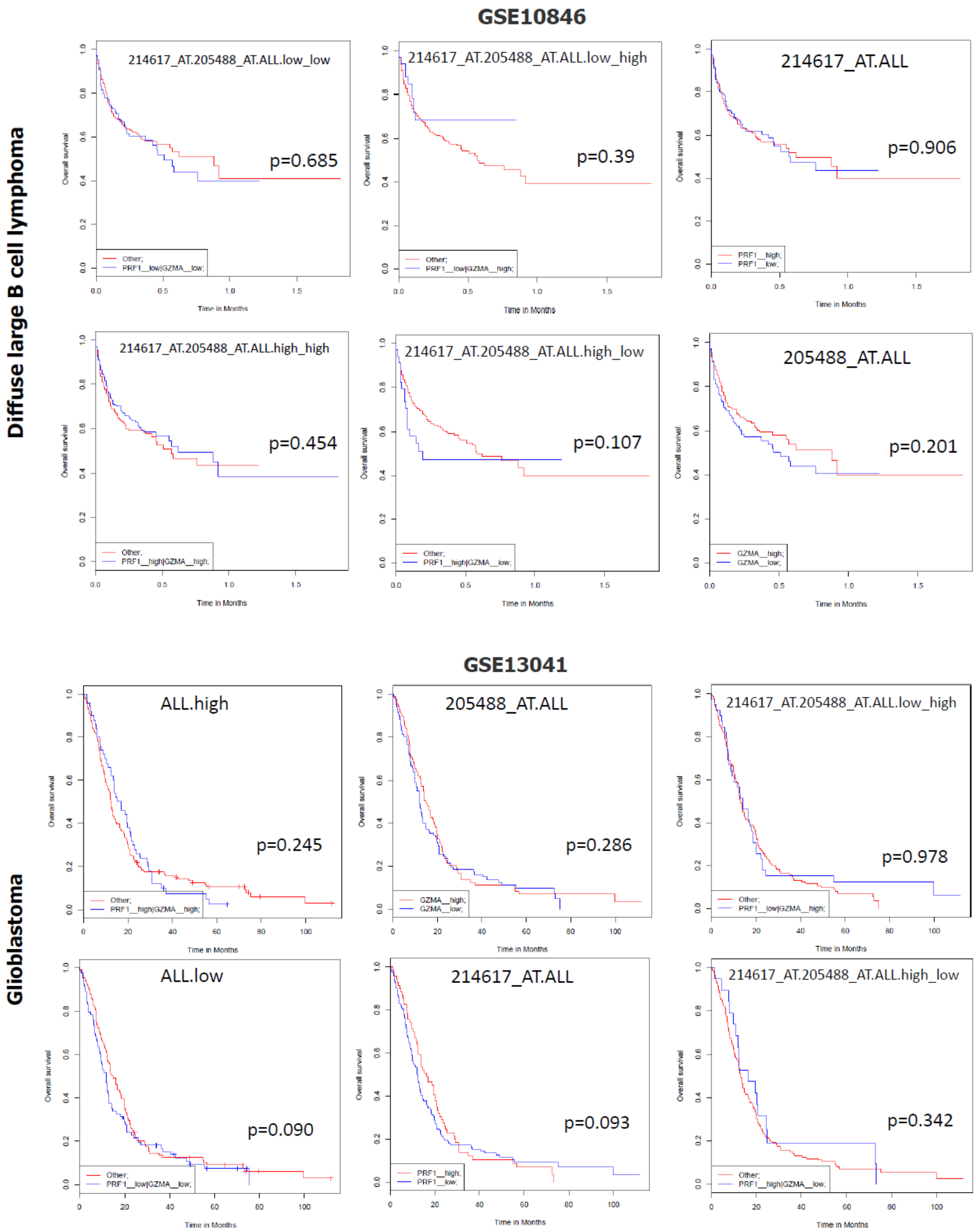
GSE10846
Diffuse large B cell lymphoma
214617_AT.205488_AT.ALL.low_low
p=0.685
Other;
PRF1__low|GZMA__low;
214617_AT.205488_AT.ALL.low_high
p=0.39
Other;
PRF1__low|GZMA__high;
214617_AT.ALL
p=0.906
PRF1__high;
PRF1__low;
214617_AT.205488_AT.ALL.high_high
p=0.454
Other;
PRF1__high|GZMA__high;
214617_AT.205488_AT.ALL.high_low
p=0.107
Other;
PRF1__high|GZMA__low;
205488_AT.ALL
p=0.201
GZMA__high;
GZMA__low;
Overall survival
Time in Months
GSE13041
Glioblastoma
ALL.high
p=0.245
Other;
PRF1__high|GZMA__high;
205488_AT.ALL
p=0.286
GZMA__high;
GZMA__low;
214617_AT.205488_AT.ALL.low_high
p=0.978
Other;
PRF1__low|GZMA__high;
ALL.low
p=0.090
Other;
PRF1__low|GZMA__low;
214617_AT.ALL
p=0.093
PRF1__high;
PRF1__low;
214617_AT.205488_AT.ALL.high_low
p=0.342
Other;
PRF1__high|GZMA__low;
Overall survival
Time in Months

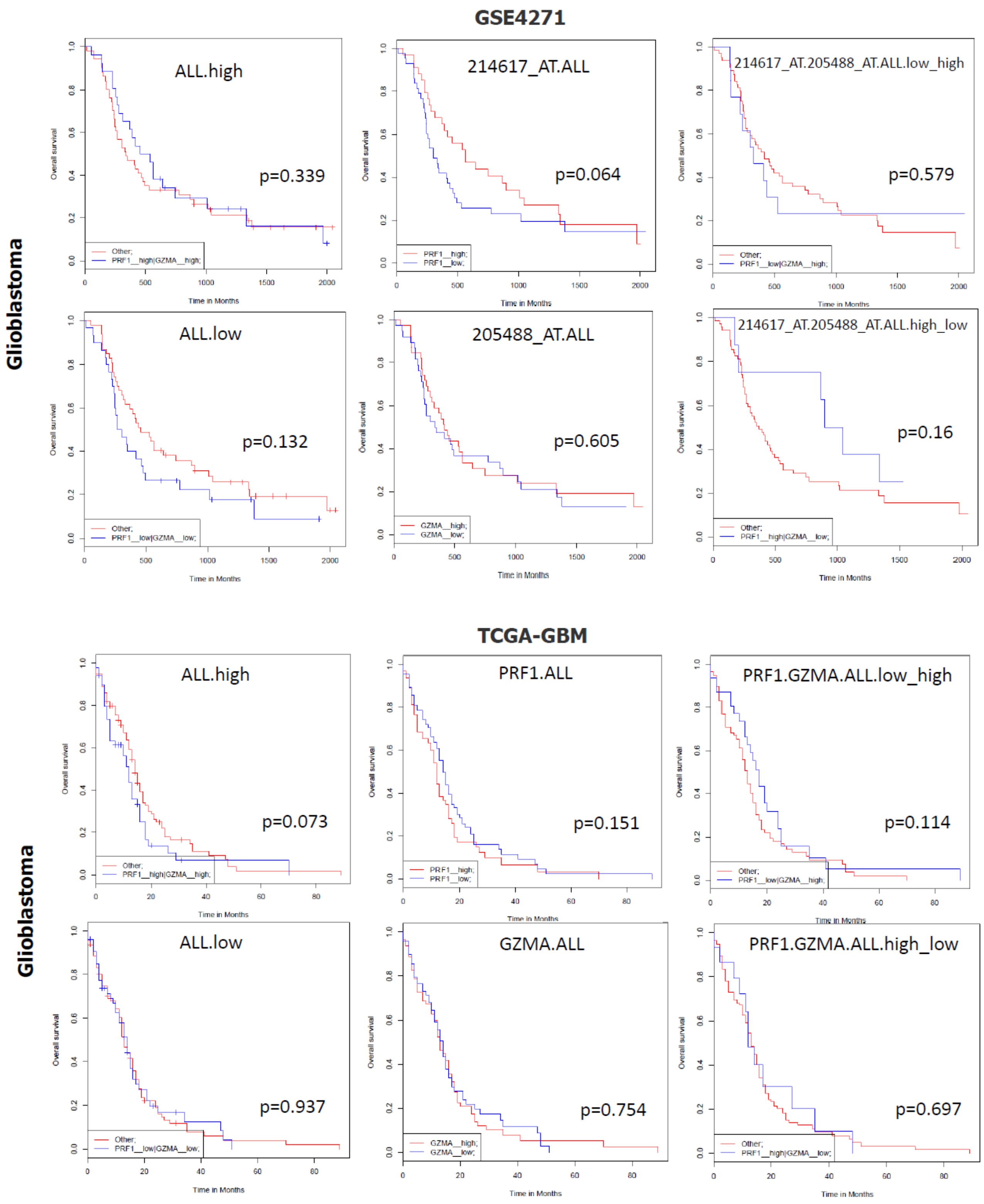
GSE4271
Glioblastoma
ALL.high
p=0.339
Other;
PRF1__high|GZMA__high;
214617_AT.ALL
p=0.064
PRF1__high;
PRF1__low;
214617_AT.205488_AT.ALL.low_high
p=0.579
Other;
PRF1__low|GZMA__high;
ALL.low
p=0.132
Other;
PRF1__low|GZMA__low;
205488_AT.ALL
p=0.605
GZMA__high;
GZMA__low;
214617_AT.205488_AT.ALL.high_low
p=0.16
Other;
PRF1__high|GZMA__low;
Overall survival
Time in Months
TCGA-GBM
Glioblastoma
ALL.high
p=0.073
Other;
PRF1__high|GZMA__high;
PRF1.ALL
p=0.151
PRF1__high;
PRF1__low;
PRF1.GZMA.ALL.low_high
p=0.114
Other;
PRF1__low|GZMA__high;
ALL.low
p=0.937
Other;
PRF1__low|GZMA__low;
GZMA.ALL
p=0.754
GZMA__high;
GZMA__low;
PRF1.GZMA.ALL.high_low
p=0.697
Other;
PRF1__high|GZMA__low;
Overall survival
Time in Months

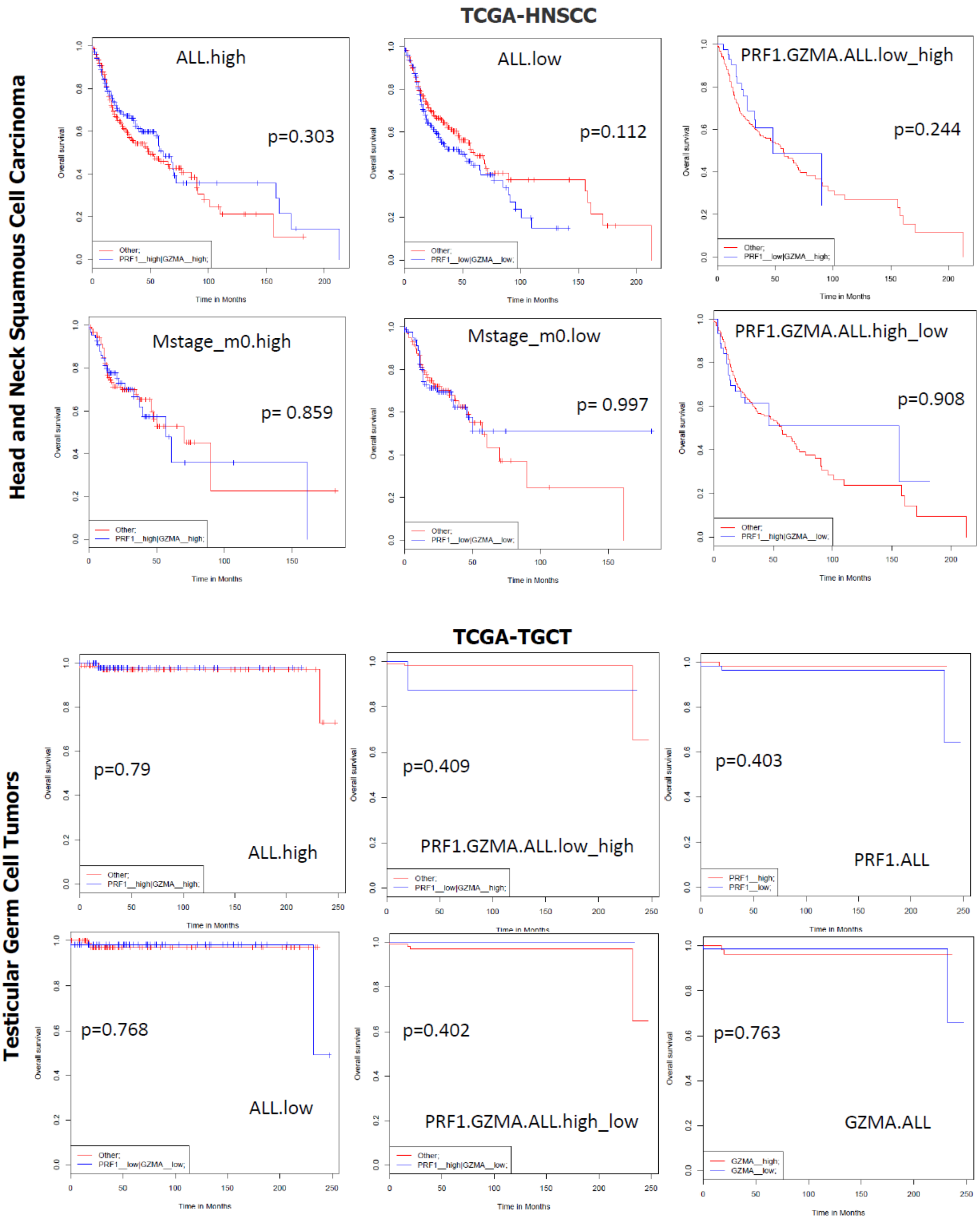
TCGA-HNSCC
Head and Neck Squamous Cell Carcinoma
ALL.high
p=0.303
ALL.low
p=0.112
PRF1.GZMA.ALL.low_high
p=0.244
Mstage_m0.high
p= 0.859
Mstage_m0.low
p= 0.997
PRF1.GZMA.ALL.high_low
p=0.908
TCGA-TGCT
Testicular Germ Cell Tumors
p=0.79
ALL.high
p=0.409
PRF1.GZMA.ALL.low_high
p=0.403
PRF1.ALL
p=0.768
ALL.low
p=0.402
PRF1.GZMA.ALL.high_low
p=0.763
GZMA.ALL
Overall survival
Time in Months

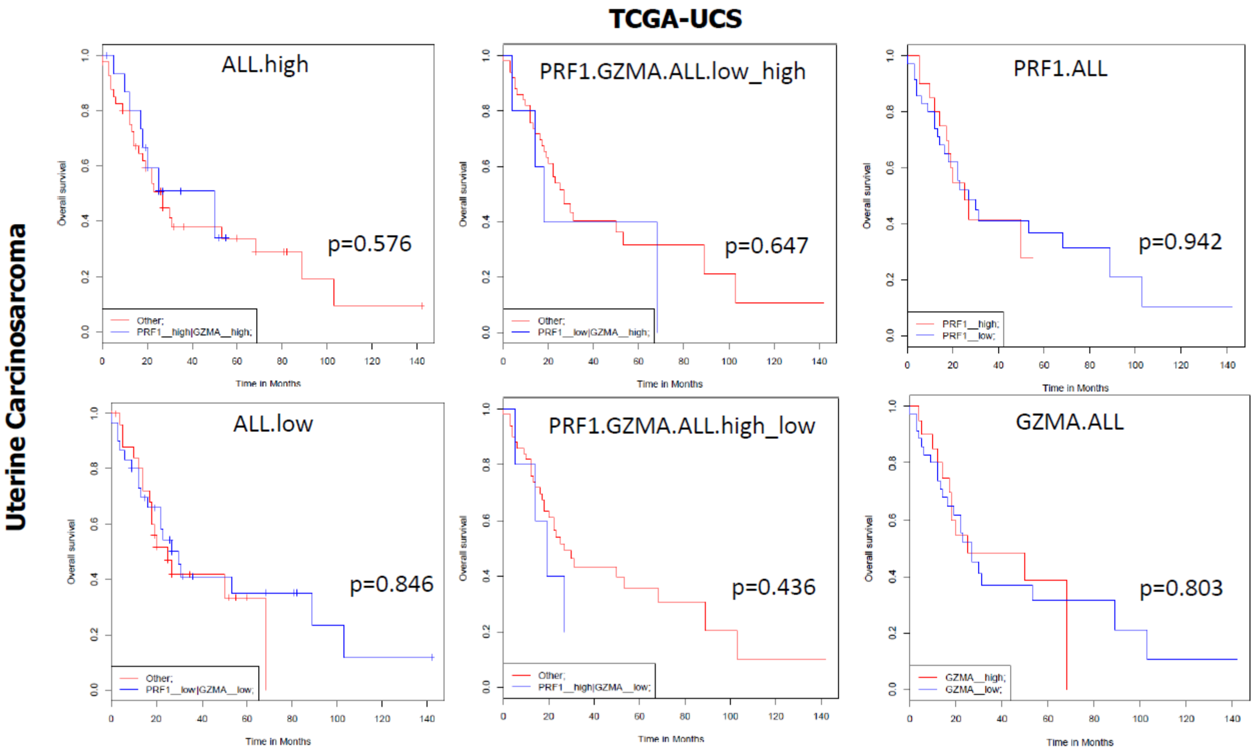

Figure S5. In diffuse large B cell lymphoma (GSE10846 and GSE32918), various combinations of distinct molecular probes for PRF1 and GZMA (PRF1, 1553681_A_AT, 214617_AT or ILMN_1740633; GZMA, 205488_AT or ILMN_1779324) could not provide any significant association with patient survival. Similar absence of significant association was also detected in glioblastoma (GSE4271, GSE13041 and TCGA-GBM) and non-metastatic head and neck squamous cell carcinomas (TCGA-HNSCC). No association or trend could be further deduced between GZMA and PRF1 expression and the survival of testicular germ cell tumor (TCGA-TGCT) or uterine carcinosarcoma patients (TCGA-UCS).
