# Supplementary material for: The Expression and Prognostic Impact of Immune Cytolytic Activity-Related Markers in Human Malignancies: A Comprehensive Meta-analysis
Source: Front Oncol. 2018 Feb 21;8:27. doi: 10.3389/fonc.2018.00027 (PMC5826382; doi:10.3389/fonc.2018.00027)
Supplement: Supplementary file 6 [file image_6.PDF]

frontiers

Supplementary Material

Title: The expression and prognostic impact of immune cytolytic activity-related markers in human malignancies: A comprehensive meta-analysis

Constantinos Roufas [1,2], Dimitrios Chasiotis [1], Anestis Makris [1], Christodoulos Efstathiades [2], Christos Dimopoulos [2], Apostolos Zaravinos [1,*]

[1] Department of Life Sciences, Biomedical Sciences Program, School of Sciences, European University Cyprus, Nicosia, Cyprus.

[2] The Center for Risk and Decision Sciences (CERIDES), Department of Computer Sciences, School of Sciences, European University Cyprus, Nicosia, Cyprus.

*** Correspondence: Apostolos Zaravinos, PhD. Biomedical Sciences Program, Department of Life Sciences, School of Sciences, European University Cyprus. 6, Diogenes Str. Engomi, P.O. Box 22006, 1516, Nicosia, Cyprus. Tel: +357-22559577. Email: a.zaravinos@euc.ac.cy**

Supplementary Figures

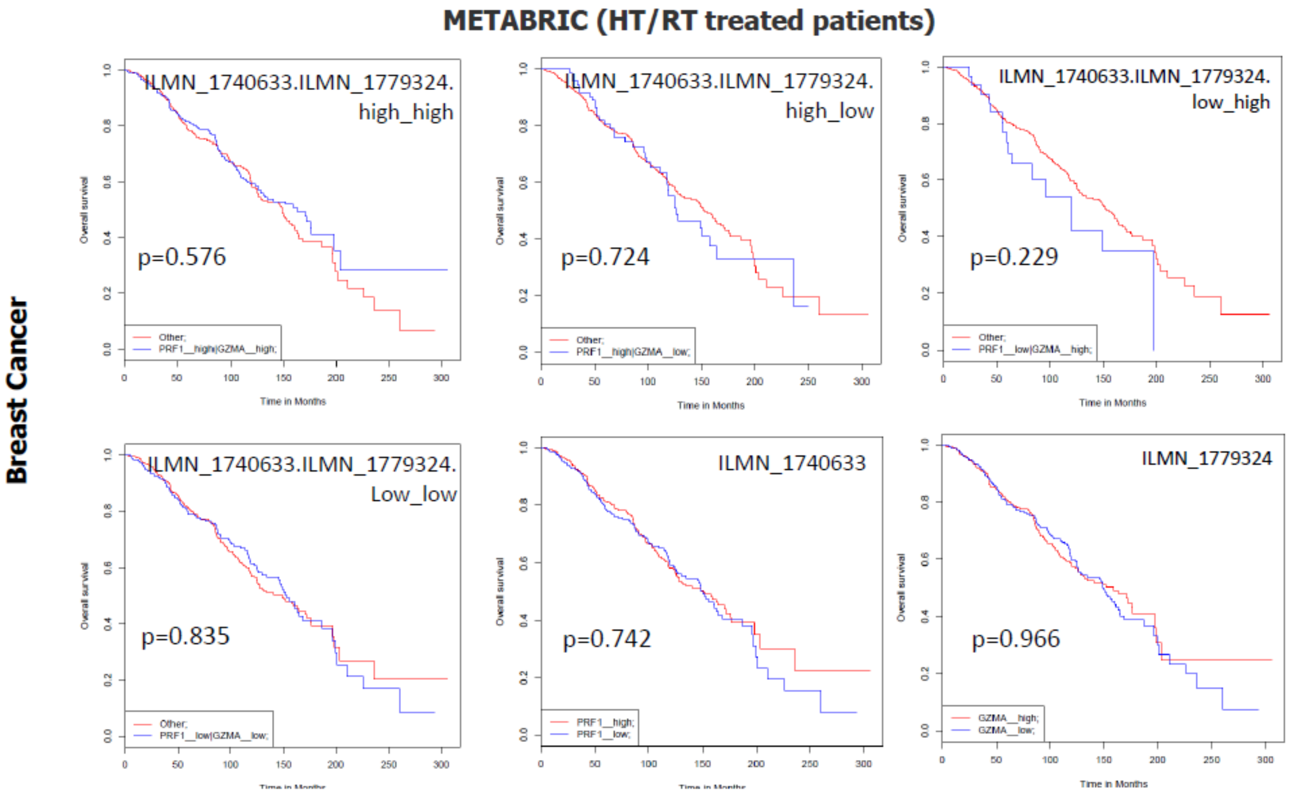

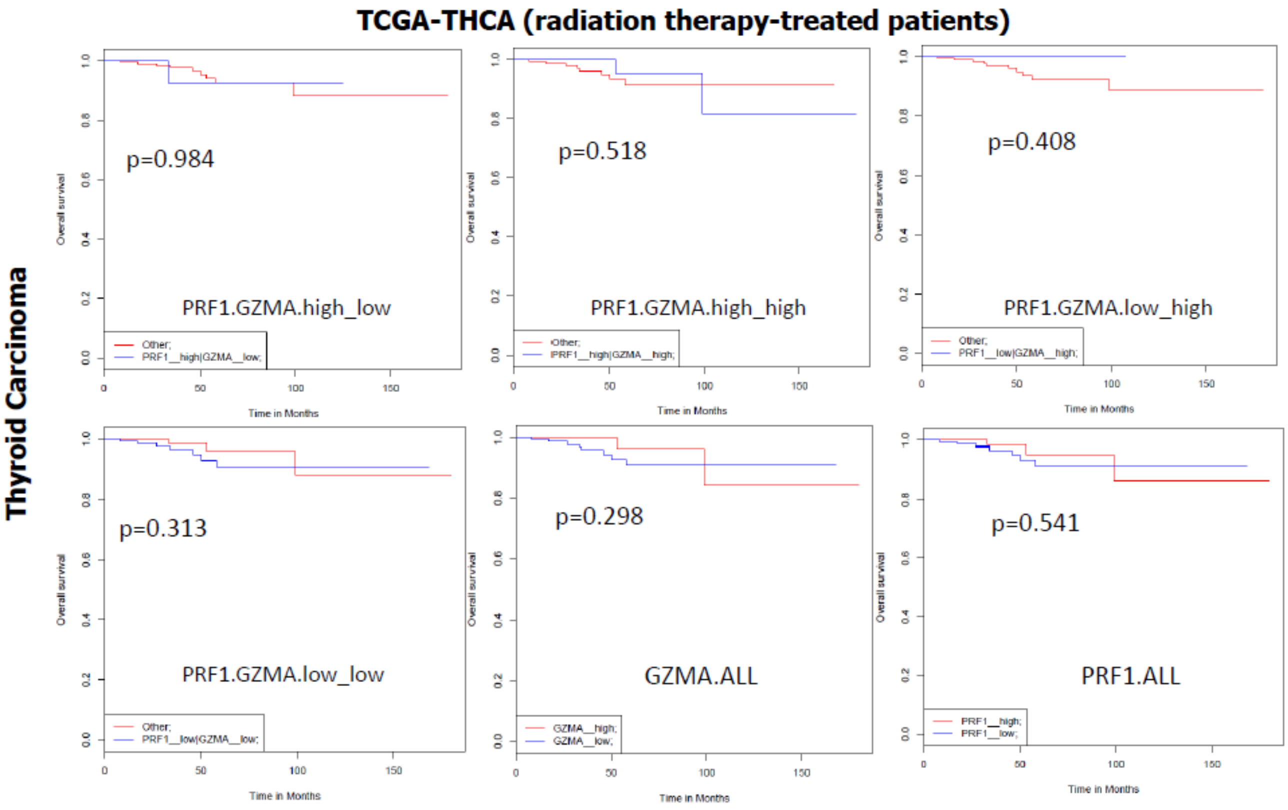

Figure S6. In the METABRIC dataset, breast cancer patients who were treated with hormonal therapy plus radiotherapy (HT/RT) (n=605) were separated from the untreated patients. Similarly, in the THCA dataset, thyroid carcinoma patients who were treated with radiotherapy (n=305) were separated from the untreated patients. The Kaplan-Meier curves were plotted using various combinations of distinct molecular probes for PRF1 and GZMA (PRF1, 1553681_A_AT, 214617_AT or ILMN_1740633; GZMA, 205488_AT or ILMN_1779324); however, without providing any significant association with patient survival.
