# Supplementary material for: The Expression and Prognostic Impact of Immune Cytolytic Activity-Related Markers in Human Malignancies: A Comprehensive Meta-analysis
Source: Front Oncol. 2018 Feb 21;8:27. doi: 10.3389/fonc.2018.00027 (PMC5826382; doi:10.3389/fonc.2018.00027)
Supplement: Supplementary file 7 [file image_7.PDF]

*Supplementary Material*

# Title: The expression and prognostic impact of immune cytolytic activity-related markers in human malignancies: A comprehensive meta-analysis

Constantinos Roufas [1,2], Dimitrios Chasiotis [1], Anestis Makris [1], Christodoulos Efstathiades [2], Christos Dimopoulos [2], Apostolos Zaravinos [1,*]

[1] Department of Life Sciences, Biomedical Sciences Program, School of Sciences, European University Cyprus, Nicosia, Cyprus.

[2] The Center for Risk and Decision Sciences (CERIDES), Department of Computer Sciences, School of Sciences, European University Cyprus, Nicosia, Cyprus.

*** Correspondence: Apostolos Zaravinos, PhD. Biomedical Sciences Program, Department of Life Sciences, School of Sciences, European University Cyprus. 6, Diogenes Str. Engomi, P.O. Box 22006, 1516, Nicosia, Cyprus. Tel: +357-22559577. Email: a.zaravinos@euc.ac.cy**

## Supplementary Figures

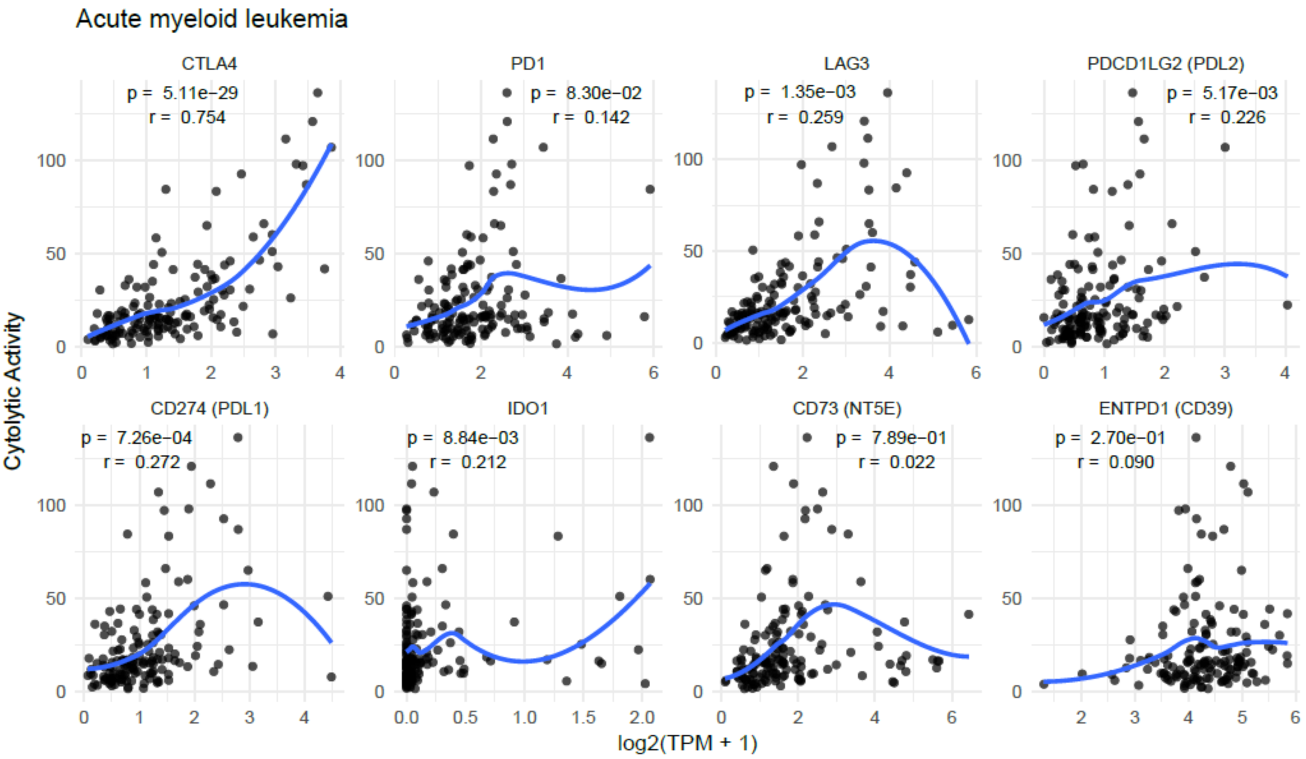


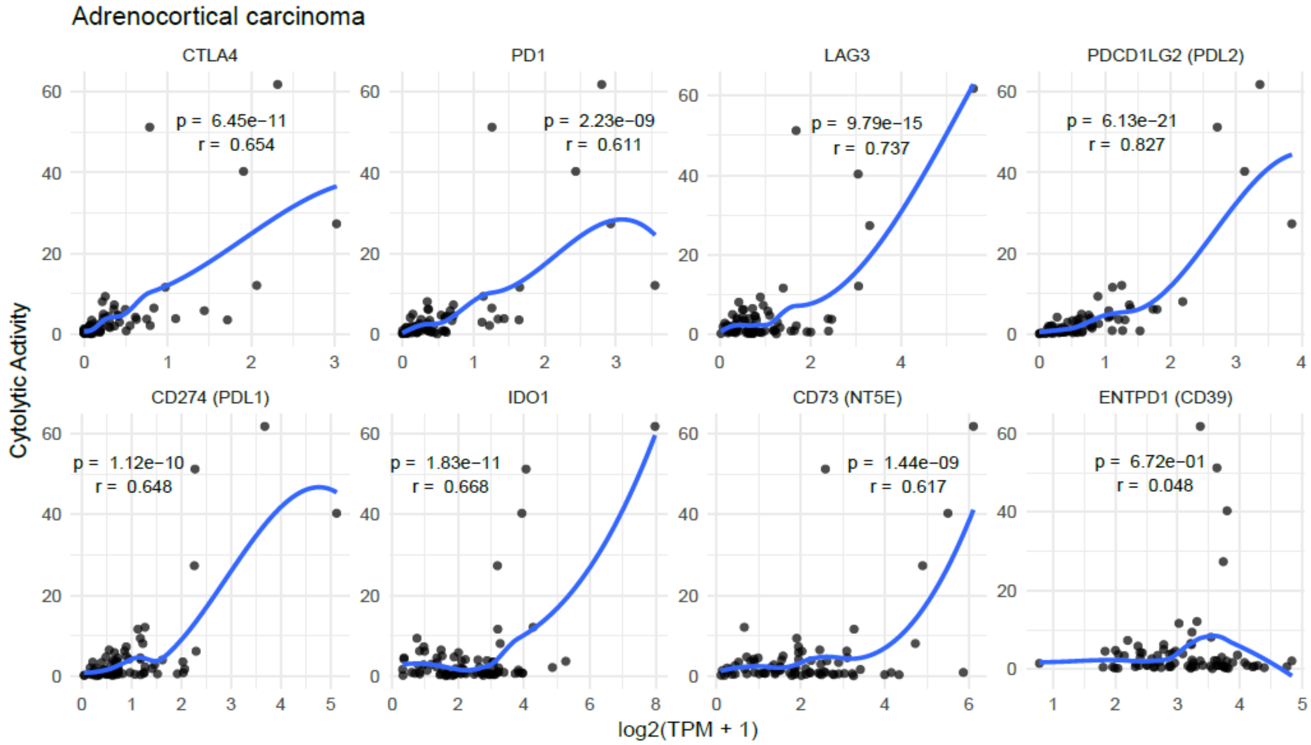

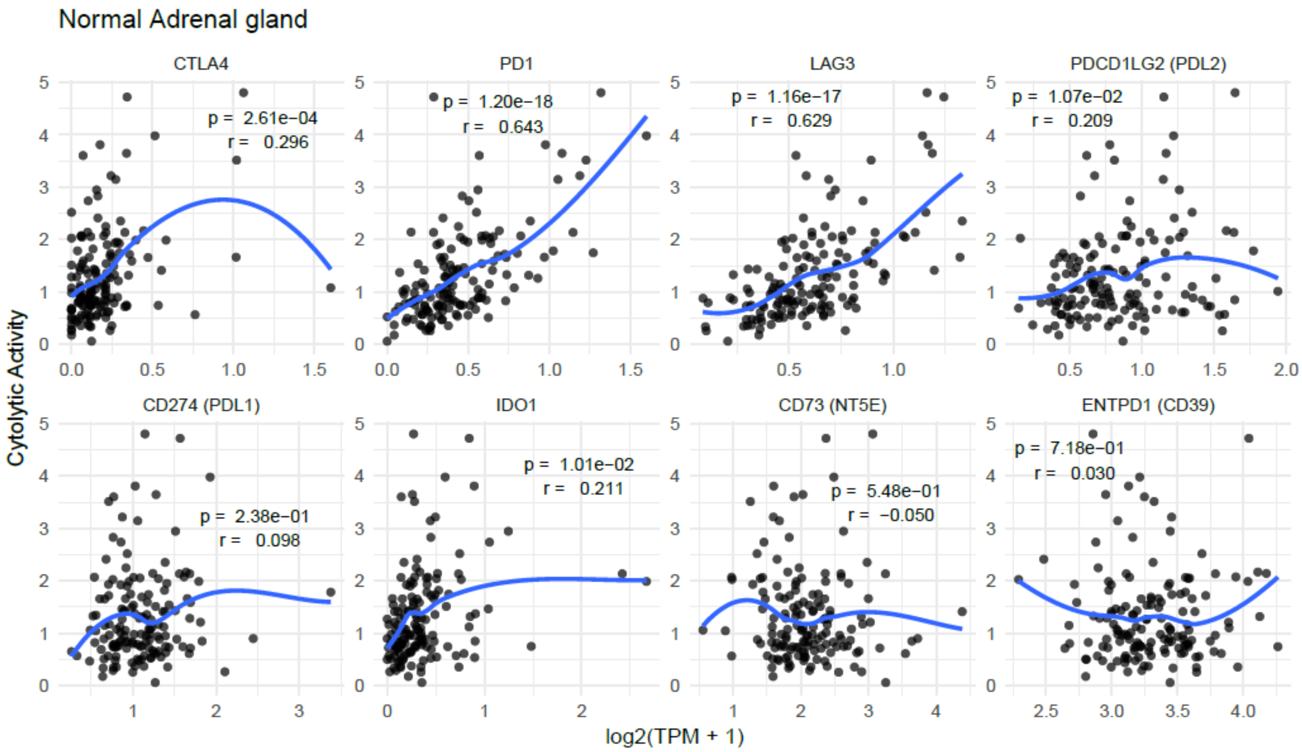
Normal Adrenal gland
CTLA4
p = 2.61e−04
r = 0.296
PD1
p = 1.20e−18
r = 0.643
LAG3
p = 1.16e−17
r = 0.629
PDCD1LG2 (PDL2)
p = 1.07e−02
r = 0.209
CD274 (PDL1)
p = 2.38e−01
r = 0.098
IDO1
p = 1.01e−02
r = 0.211
CD73 (NT5E)
p = 5.48e−01
r = −0.050
ENTPD1 (CD39)
p = 7.18e−01
r = 0.030
Cytolytic Activity
log2(TPM + 1)

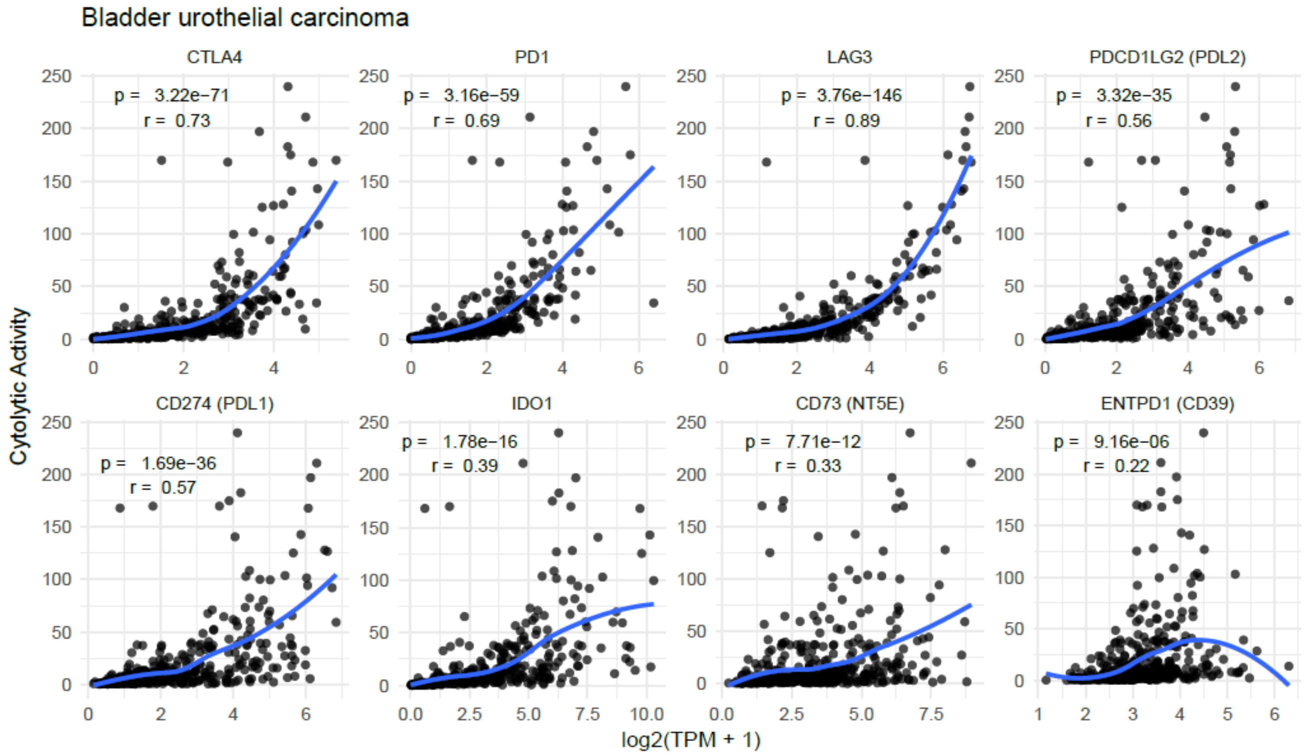
Bladder urothelial carcinoma
CTLA4
p = 3.22e−71
r = 0.73
PD1
p = 3.16e−59
r = 0.69
LAG3
p = 3.76e−146
r = 0.89
PDCD1LG2 (PDL2)
p = 3.32e−35
r = 0.56
CD274 (PDL1)
p = 1.69e−36
r = 0.57
IDO1
p = 1.78e−16
r = 0.39
CD73 (NT5E)
p = 7.71e−12
r = 0.33
ENTPD1 (CD39)
p = 9.16e−06
r = 0.22
Cytolytic Activity
log2(TPM + 1)

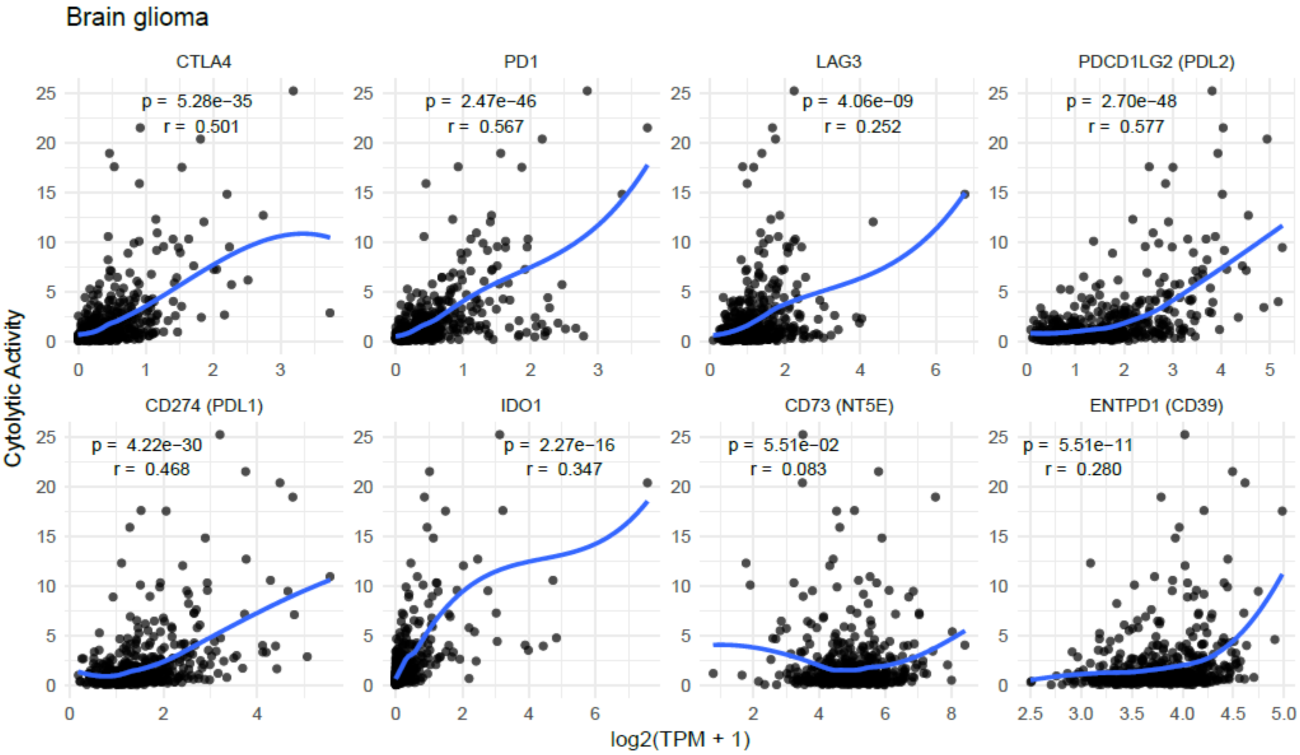
Brain glioma
CTLA4
p = 5.28e−35
r = 0.501
PD1
p = 2.47e−46
r = 0.567
LAG3
p = 4.06e−09
r = 0.252
PDCD1LG2 (PDL2)
p = 2.70e−48
r = 0.577
CD274 (PDL1)
p = 4.22e−30
r = 0.468
IDO1
p = 2.27e−16
r = 0.347
CD73 (NT5E)
p = 5.51e−02
r = 0.083
ENTPD1 (CD39)
p = 5.51e−11
r = 0.280
Cytolytic Activity
log2(TPM + 1)

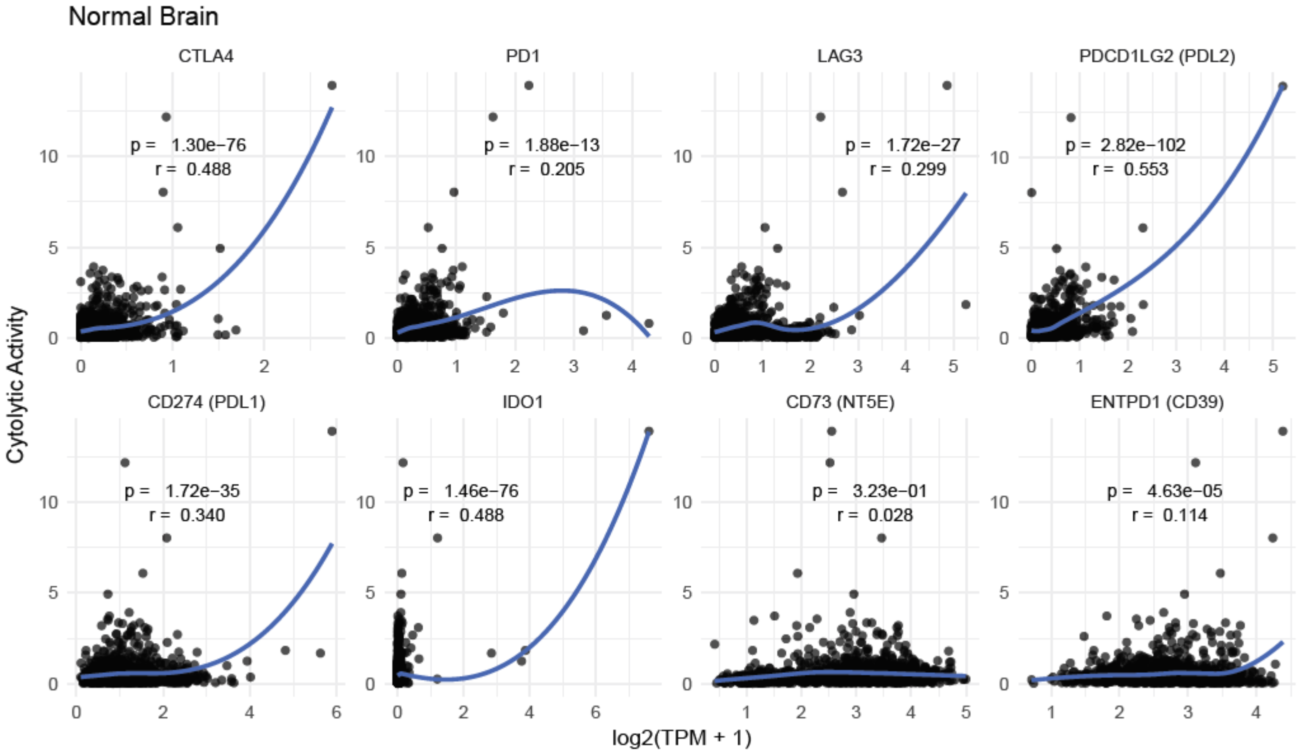
Normal Brain
CTLA4
p = 1.30e−76
r = 0.488
PD1
p = 1.88e−13
r = 0.205
LAG3
p = 1.72e−27
r = 0.299
PDCD1LG2 (PDL2)
p = 2.82e−102
r = 0.553
CD274 (PDL1)
p = 1.72e−35
r = 0.340
IDO1
p = 1.46e−76
r = 0.488
CD73 (NT5E)
p = 3.23e−01
r = 0.028
ENTPD1 (CD39)
p = 4.63e−05
r = 0.114
Cytolytic Activity
log2(TPM + 1)

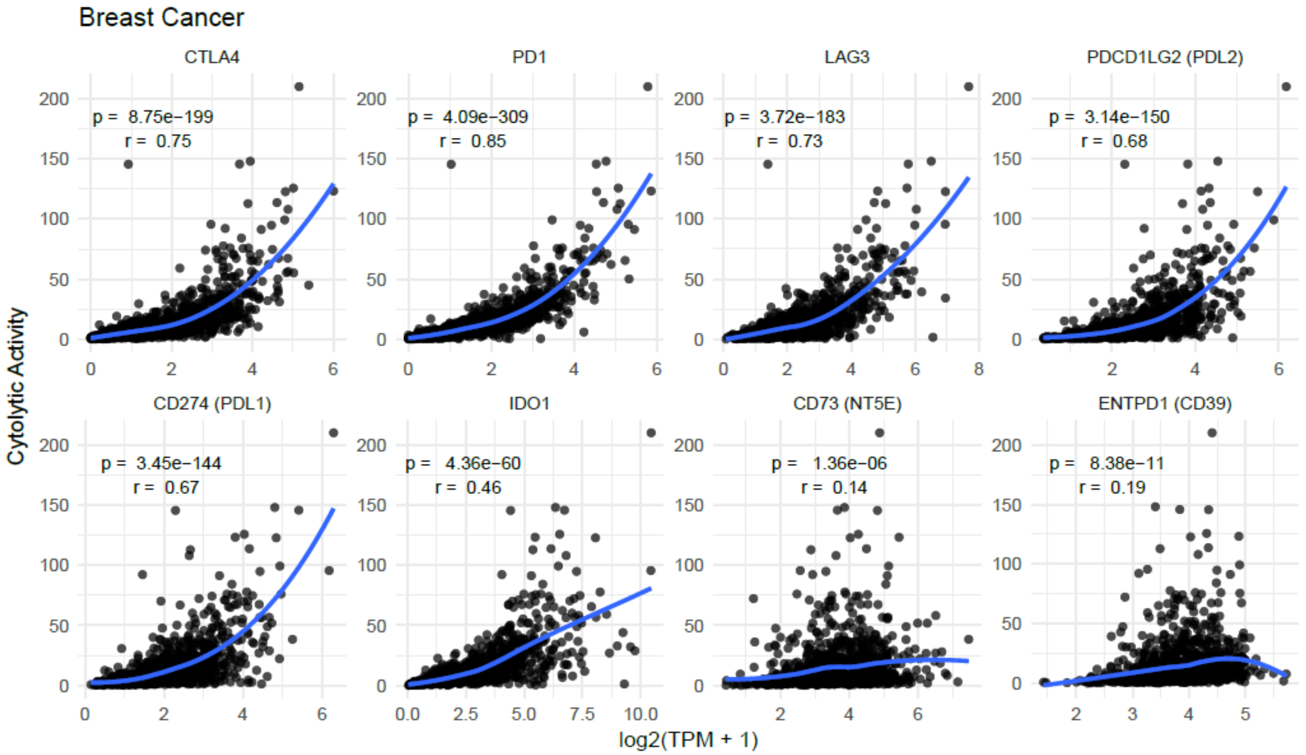
Breast Cancer
CTLA4
p = 8.75e−199
r = 0.75
PD1
p = 4.09e−309
r = 0.85
LAG3
p = 3.72e−183
r = 0.73
PDCD1LG2 (PDL2)
p = 3.14e−150
r = 0.68
CD274 (PDL1)
p = 3.45e−144
r = 0.67
IDO1
p = 4.36e−60
r = 0.46
CD73 (NT5E)
p = 1.36e−06
r = 0.14
ENTPD1 (CD39)
p = 8.38e−11
r = 0.19
Cytolytic Activity
log2(TPM + 1)

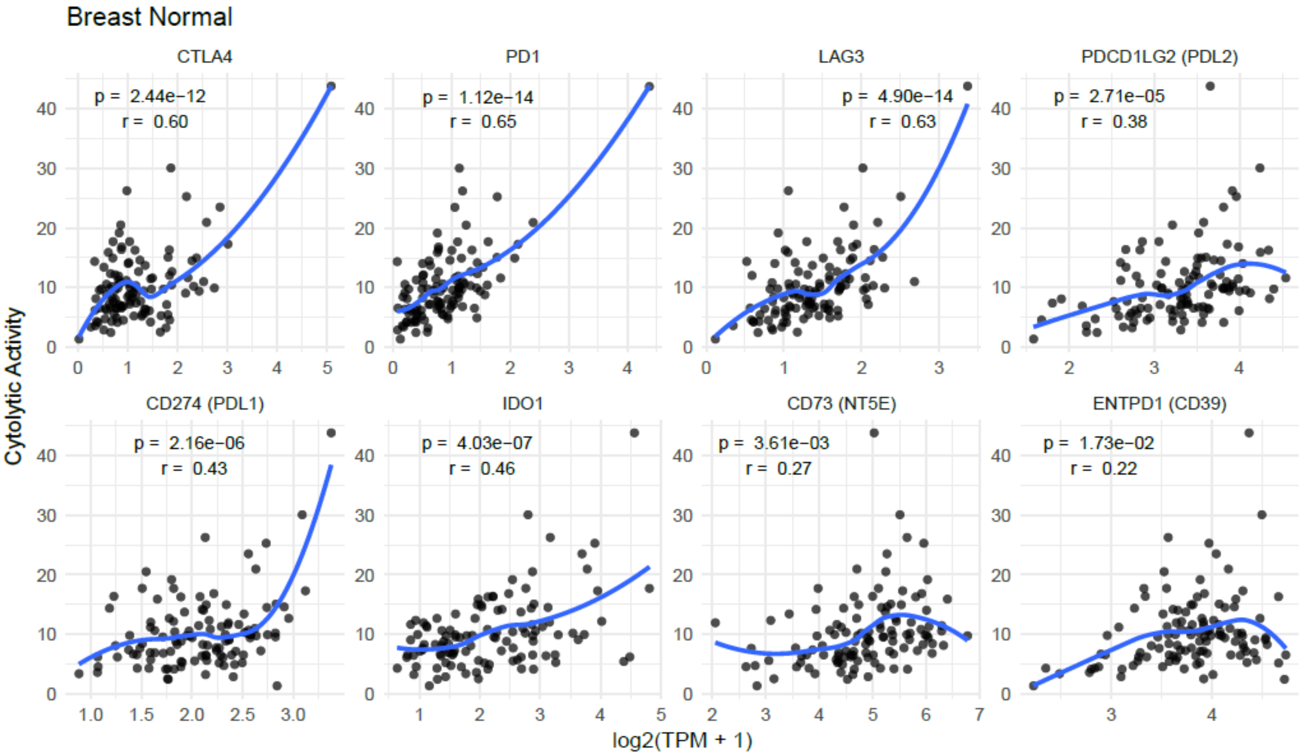
Breast Normal
CTLA4
p = 2.44e−12
r = 0.60
PD1
p = 1.12e−14
r = 0.65
LAG3
p = 4.90e−14
r = 0.63
PDCD1LG2 (PDL2)
p = 2.71e−05
r = 0.38
CD274 (PDL1)
p = 2.16e−06
r = 0.43
IDO1
p = 4.03e−07
r = 0.46
CD73 (NT5E)
p = 3.61e−03
r = 0.27
ENTPD1 (CD39)
p = 1.73e−02
r = 0.22
Cytolytic Activity
log2(TPM + 1)

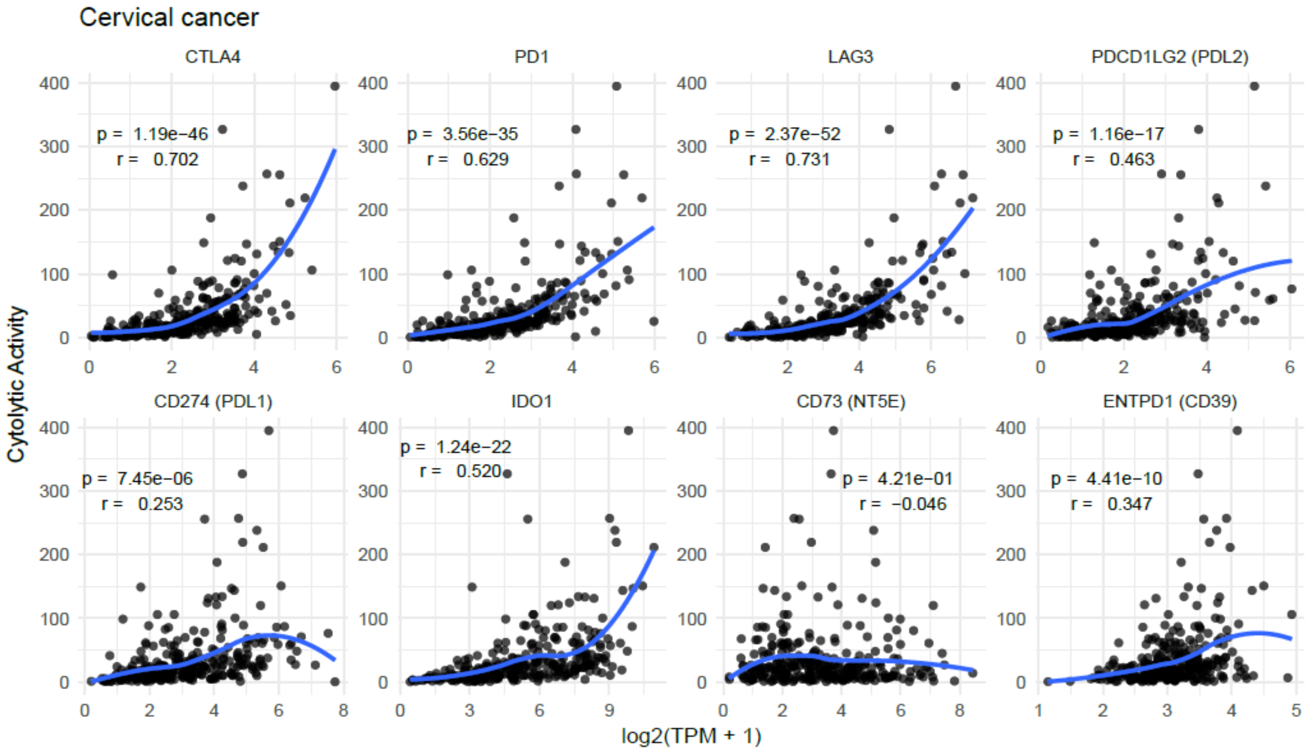
Cervical cancer
CTLA4
p = 1.19e−46
r = 0.702
PD1
p = 3.56e−35
r = 0.629
LAG3
p = 2.37e−52
r = 0.731
PDCD1LG2 (PDL2)
p = 1.16e−17
r = 0.463
CD274 (PDL1)
p = 7.45e−06
r = 0.253
IDO1
p = 1.24e−22
r = 0.520
CD73 (NT5E)
p = 4.21e−01
r = −0.046
ENTPD1 (CD39)
p = 4.41e−10
r = 0.347
Cytolytic Activity
log2(TPM + 1)

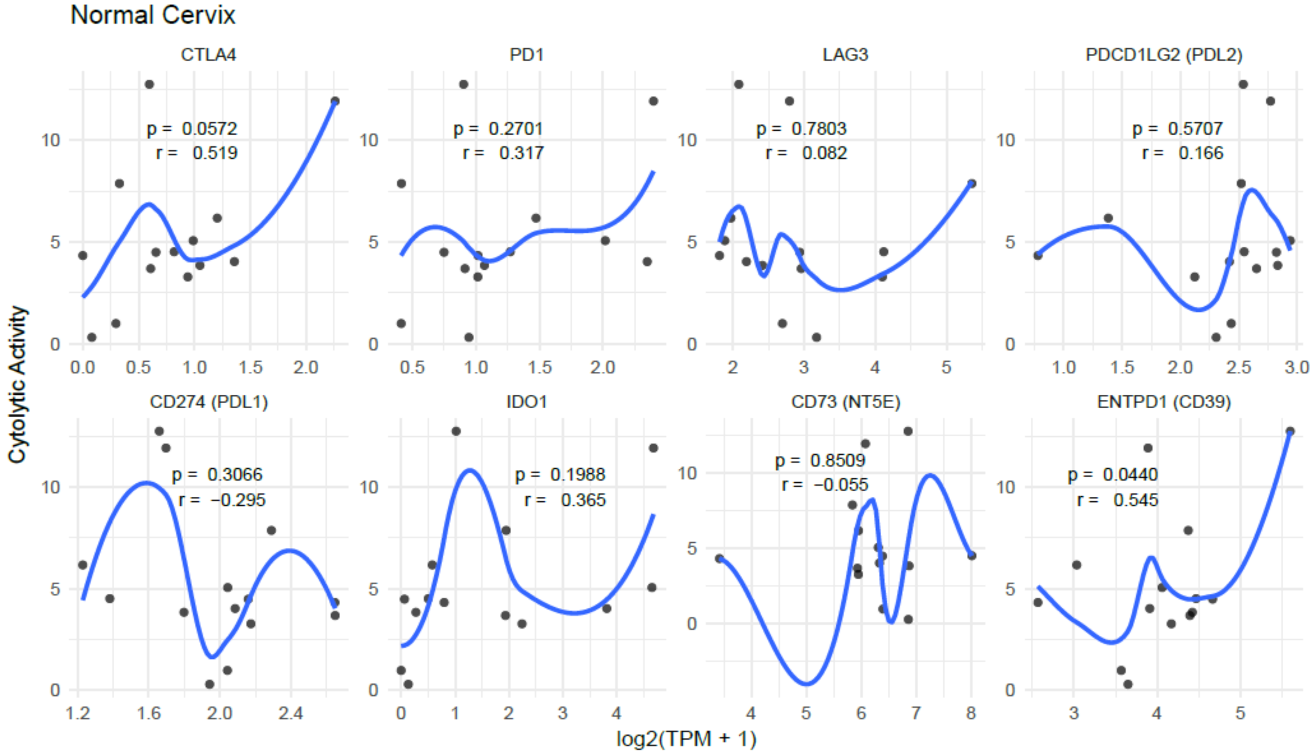
Normal Cervix
CTLA4
p = 0.0572
r = 0.519
PD1
p = 0.2701
r = 0.317
LAG3
p = 0.7803
r = 0.082
PDCD1LG2 (PDL2)
p = 0.5707
r = 0.166
CD274 (PDL1)
p = 0.3066
r = −0.295
IDO1
p = 0.1988
r = 0.365
CD73 (NT5E)
p = 0.8509
r = −0.055
ENTPD1 (CD39)
p = 0.0440
r = 0.545
Cytolytic Activity
log2(TPM + 1)

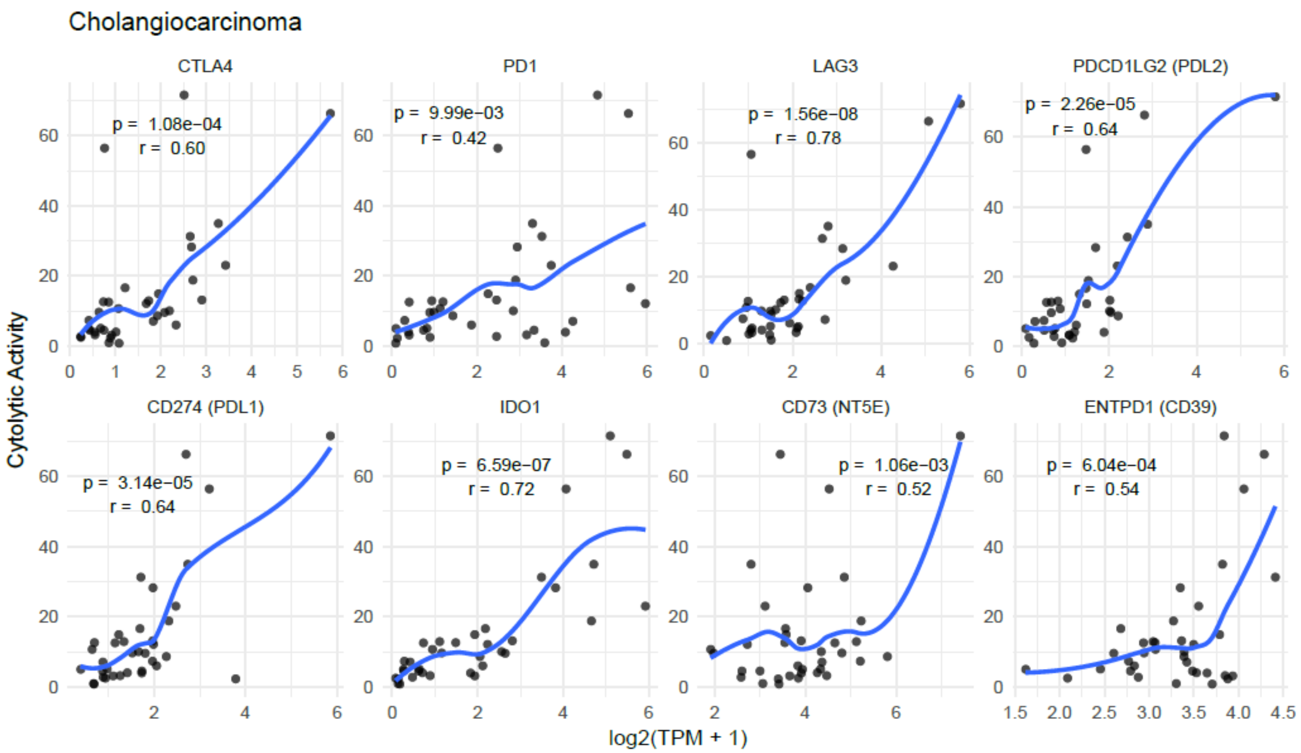
Cholangiocarcinoma
CTLA4
p = 1.08e−04
r = 0.60
PD1
p = 9.99e−03
r = 0.42
LAG3
p = 1.56e−08
r = 0.78
PDCD1LG2 (PDL2)
p = 2.26e−05
r = 0.64
CD274 (PDL1)
p = 3.14e−05
r = 0.64
IDO1
p = 6.59e−07
r = 0.72
CD73 (NT5E)
p = 1.06e−03
r = 0.52
ENTPD1 (CD39)
p = 6.04e−04
r = 0.54
Cytolytic Activity
log2(TPM + 1)

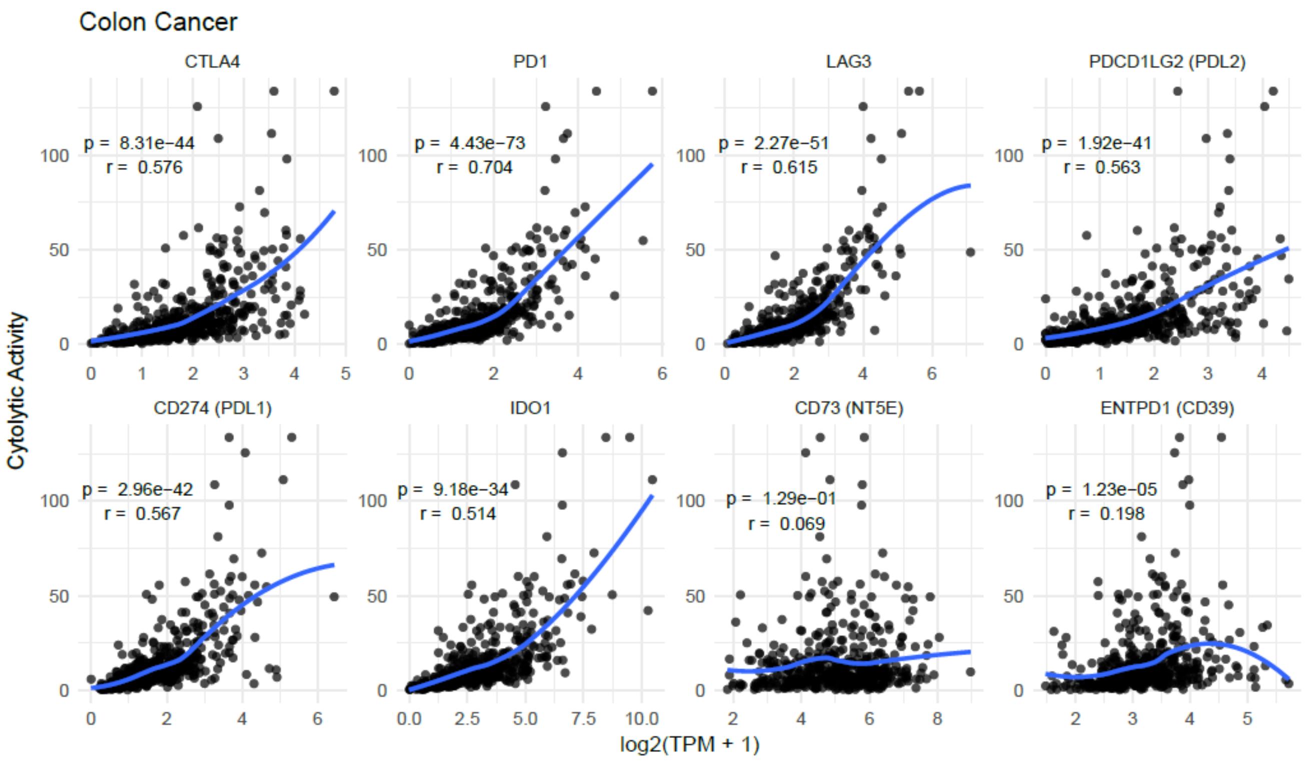
Colon Cancer
CTLA4
p = 8.31e−44
r = 0.576
PD1
p = 4.43e−73
r = 0.704
LAG3
p = 2.27e−51
r = 0.615
PDCD1LG2 (PDL2)
p = 1.92e−41
r = 0.563
CD274 (PDL1)
p = 2.96e−42
r = 0.567
IDO1
p = 9.18e−34
r = 0.514
CD73 (NT5E)
p = 1.29e−01
r = 0.069
ENTPD1 (CD39)
p = 1.23e−05
r = 0.198
Cytolytic Activity
log2(TPM + 1)

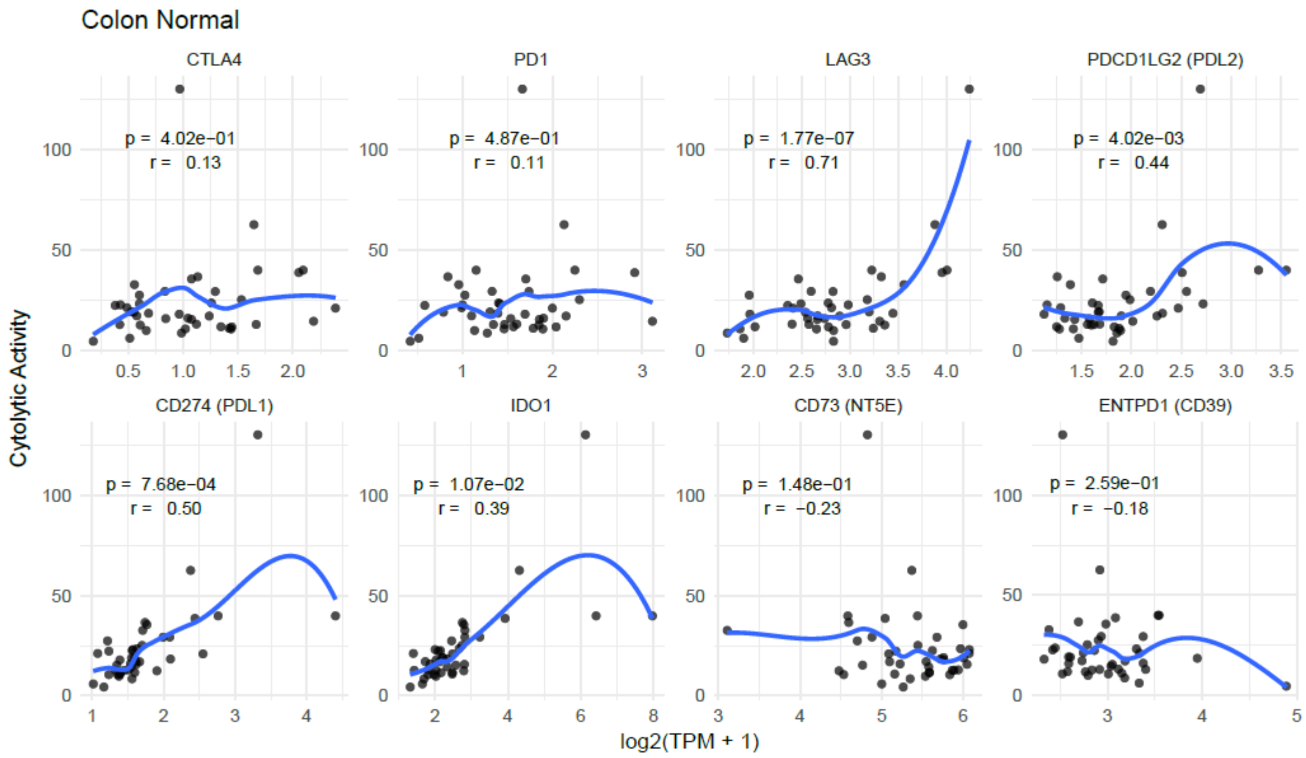
Colon Normal
CTLA4
p = 4.02e−01
r = 0.13
PD1
p = 4.87e−01
r = 0.11
LAG3
p = 1.77e−07
r = 0.71
PDCD1LG2 (PDL2)
p = 4.02e−03
r = 0.44
CD274 (PDL1)
p = 7.68e−04
r = 0.50
IDO1
p = 1.07e−02
r = 0.39
CD73 (NT5E)
p = 1.48e−01
r = −0.23
ENTPD1 (CD39)
p = 2.59e−01
r = −0.18
Cytolytic Activity
log2(TPM + 1)

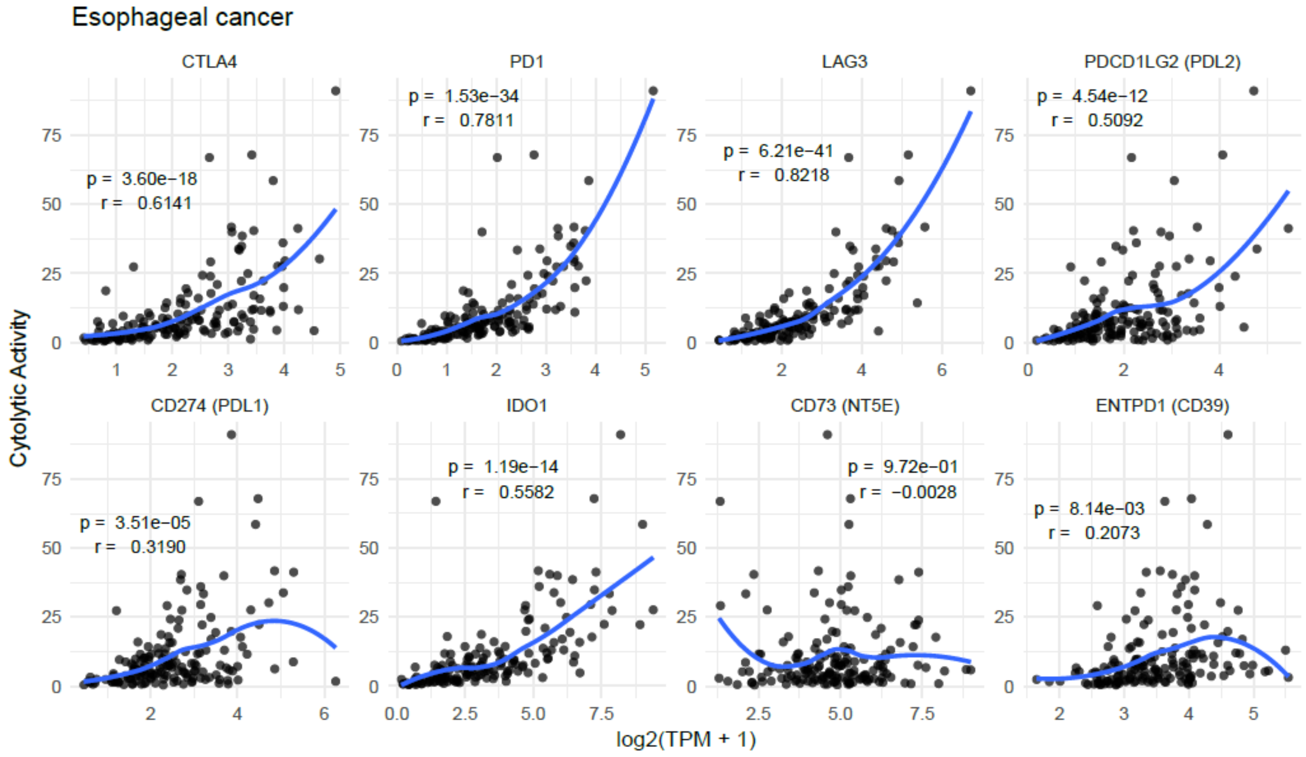
Esophageal cancer
CTLA4
p = 3.60e−18
r = 0.6141
PD1
p = 1.53e−34
r = 0.7811
LAG3
p = 6.21e−41
r = 0.8218
PDCD1LG2 (PDL2)
p = 4.54e−12
r = 0.5092
CD274 (PDL1)
p = 3.51e−05
r = 0.3190
IDO1
p = 1.19e−14
r = 0.5582
CD73 (NT5E)
p = 9.72e−01
r = −0.0028
ENTPD1 (CD39)
p = 8.14e−03
r = 0.2073
Cytolytic Activity
log2(TPM + 1)

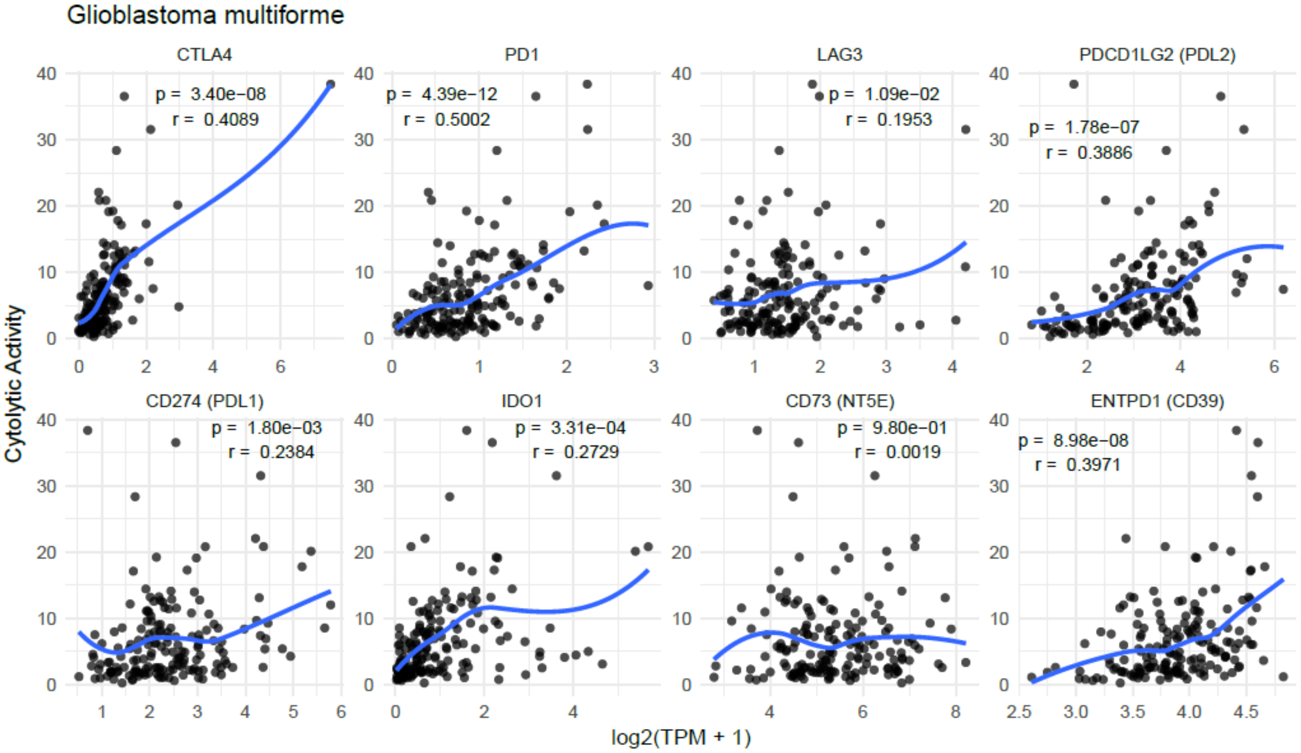
Glioblastoma multiforme
CTLA4
p = 3.40e−08
r = 0.4089
PD1
p = 4.39e−12
r = 0.5002
LAG3
p = 1.09e−02
r = 0.1953
PDCD1LG2 (PDL2)
p = 1.78e−07
r = 0.3886
CD274 (PDL1)
p = 1.80e−03
r = 0.2384
IDO1
p = 3.31e−04
r = 0.2729
CD73 (NT5E)
p = 9.80e−01
r = 0.0019
ENTPD1 (CD39)
p = 8.98e−08
r = 0.3971
Cytolytic Activity
log2(TPM + 1)

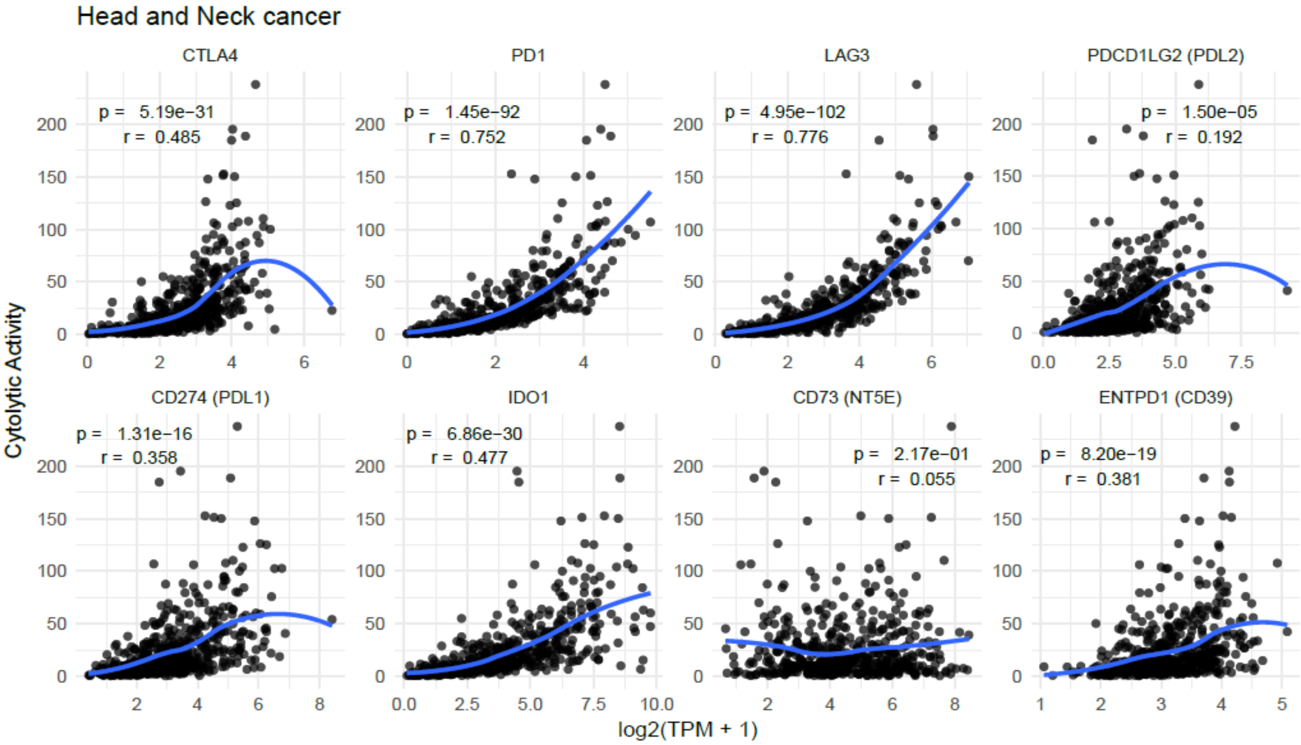
Head and Neck cancer
CTLA4
p = 5.19e−31
r = 0.485
PD1
p = 1.45e−92
r = 0.752
LAG3
p = 4.95e−102
r = 0.776
PDCD1LG2 (PDL2)
p = 1.50e−05
r = 0.192
CD274 (PDL1)
p = 1.31e−16
r = 0.358
IDO1
p = 6.86e−30
r = 0.477
CD73 (NT5E)
p = 2.17e−01
r = 0.055
ENTPD1 (CD39)
p = 8.20e−19
r = 0.381
Cytolytic Activity
log2(TPM + 1)

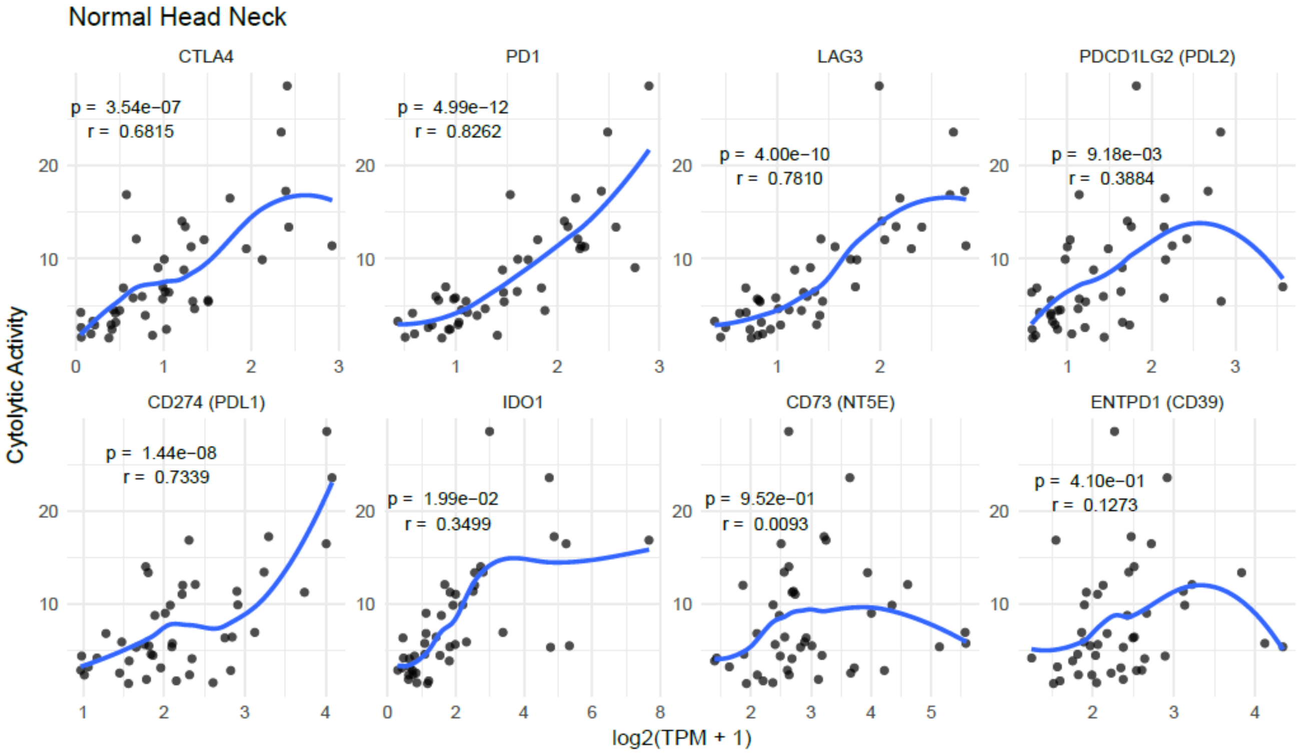
Normal Head Neck
CTLA4
p = 3.54e−07
r = 0.6815
PD1
p = 4.99e−12
r = 0.8262
LAG3
p = 4.00e−10
r = 0.7810
PDCD1LG2 (PDL2)
p = 9.18e−03
r = 0.3884
CD274 (PDL1)
p = 1.44e−08
r = 0.7339
IDO1
p = 1.99e−02
r = 0.3499
CD73 (NT5E)
p = 9.52e−01
r = 0.0093
ENTPD1 (CD39)
p = 4.10e−01
r = 0.1273
Cytolytic Activity
log2(TPM + 1)

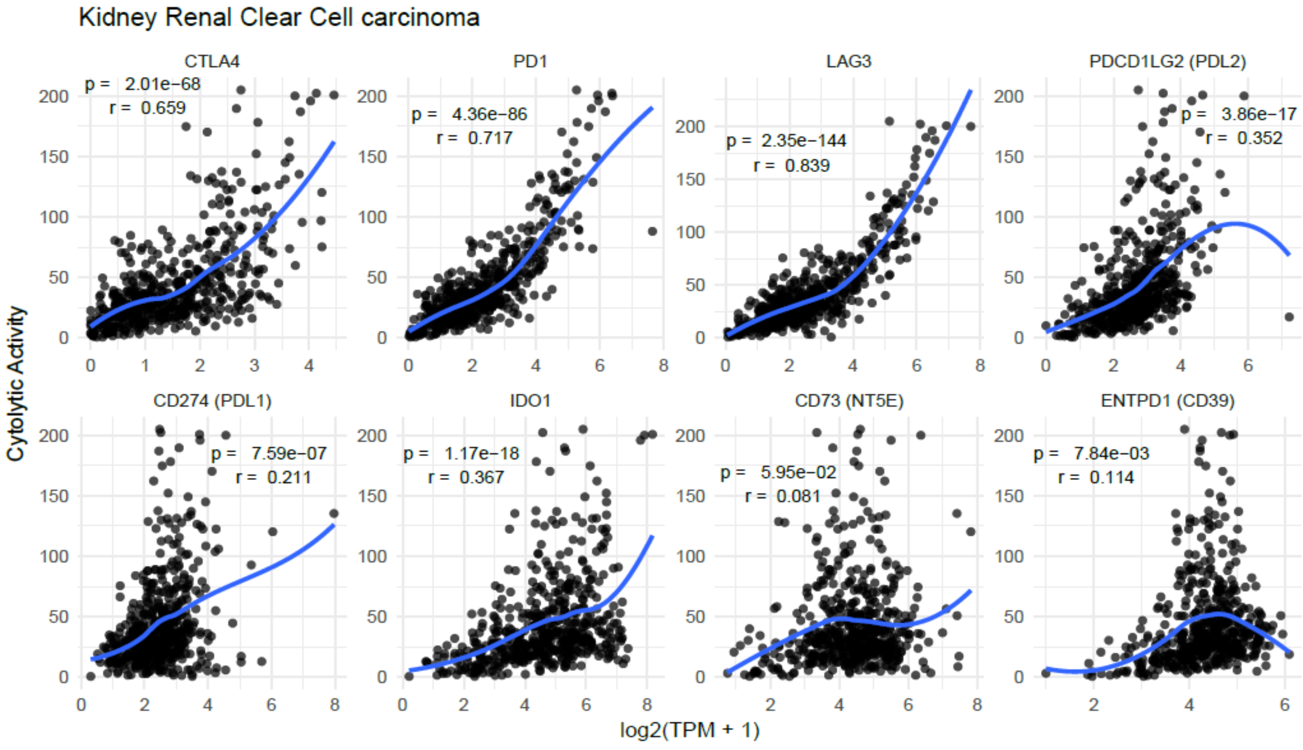
Kidney Renal Clear Cell carcinoma
CTLA4
p = 2.01e−68
r = 0.659
PD1
p = 4.36e−86
r = 0.717
LAG3
p = 2.35e−144
r = 0.839
PDCD1LG2 (PDL2)
p = 3.86e−17
r = 0.352
CD274 (PDL1)
p = 7.59e−07
r = 0.211
IDO1
p = 1.17e−18
r = 0.367
CD73 (NT5E)
p = 5.95e−02
r = 0.081
ENTPD1 (CD39)
p = 7.84e−03
r = 0.114
Cytolytic Activity
log2(TPM + 1)

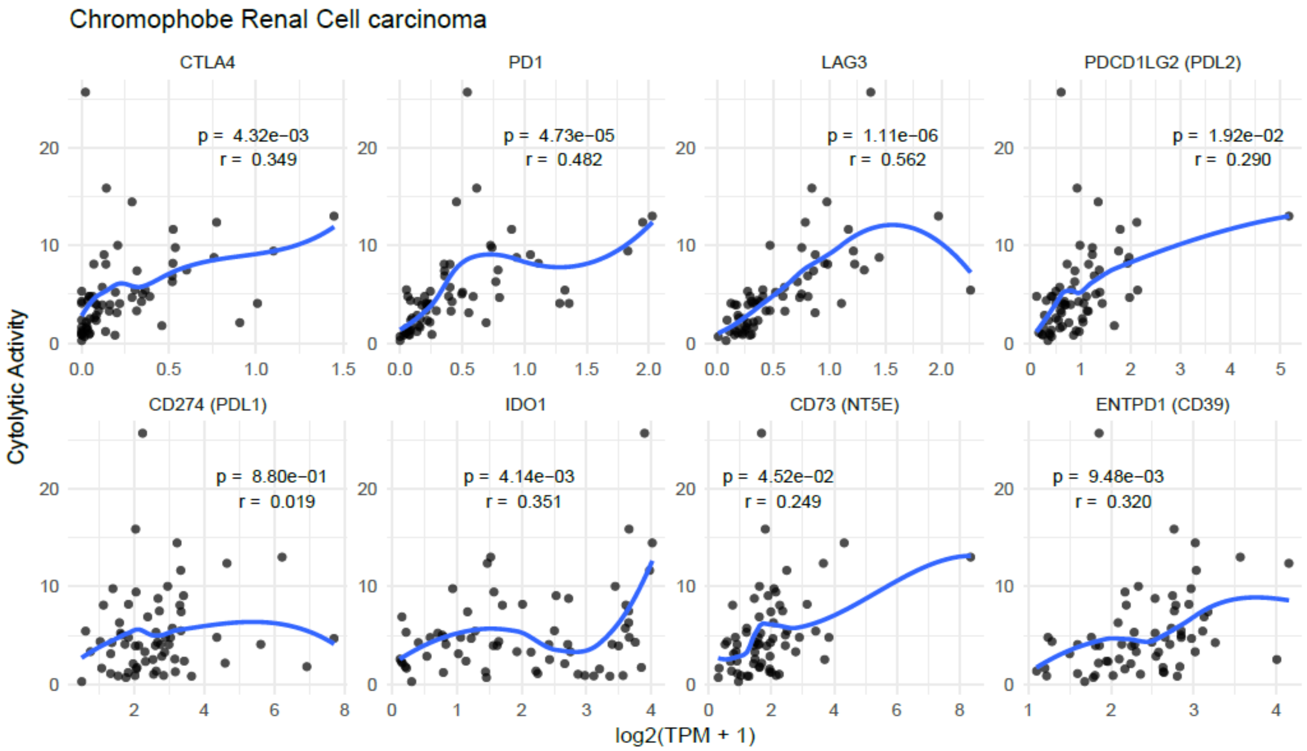
Chromophobe Renal Cell carcinoma
CTLA4
p = 4.32e−03
r = 0.349
PD1
p = 4.73e−05
r = 0.482
LAG3
p = 1.11e−06
r = 0.562
PDCD1LG2 (PDL2)
p = 1.92e−02
r = 0.290
CD274 (PDL1)
p = 8.80e−01
r = 0.019
IDO1
p = 4.14e−03
r = 0.351
CD73 (NT5E)
p = 4.52e−02
r = 0.249
ENTPD1 (CD39)
p = 9.48e−03
r = 0.320
Cytolytic Activity
log2(TPM + 1)

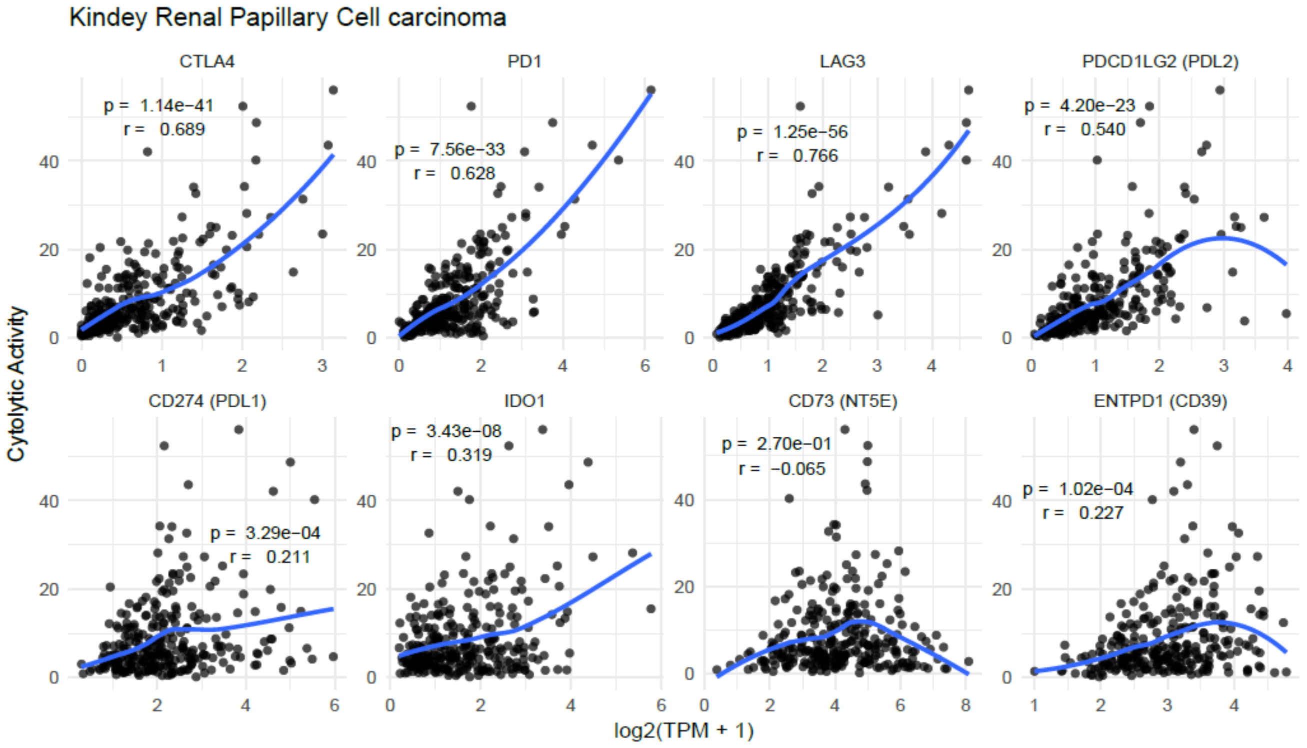
Kindey Renal Papillary Cell carcinoma
CTLA4
p = 1.14e−41
r = 0.689
PD1
p = 7.56e−33
r = 0.628
LAG3
p = 1.25e−56
r = 0.766
PDCD1LG2 (PDL2)
p = 4.20e−23
r = 0.540
CD274 (PDL1)
p = 3.29e−04
r = 0.211
IDO1
p = 3.43e−08
r = 0.319
CD73 (NT5E)
p = 2.70e−01
r = −0.065
ENTPD1 (CD39)
p = 1.02e−04
r = 0.227
Cytolytic Activity
log2(TPM + 1)

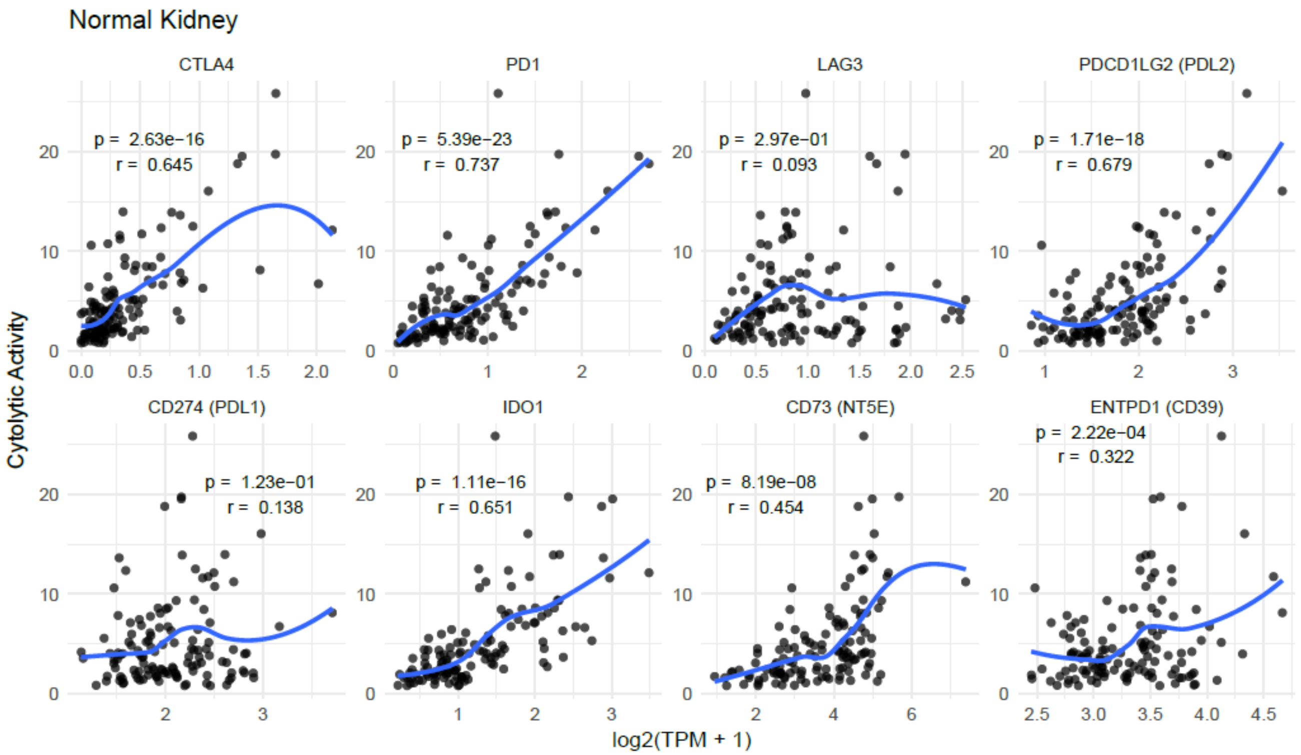
Normal Kidney
CTLA4
p = 2.63e−16
r = 0.645
PD1
p = 5.39e−23
r = 0.737
LAG3
p = 2.97e−01
r = 0.093
PDCD1LG2 (PDL2)
p = 1.71e−18
r = 0.679
CD274 (PDL1)
p = 1.23e−01
r = 0.138
IDO1
p = 1.11e−16
r = 0.651
CD73 (NT5E)
p = 8.19e−08
r = 0.454
ENTPD1 (CD39)
p = 2.22e−04
r = 0.322
Cytolytic Activity
log2(TPM + 1)

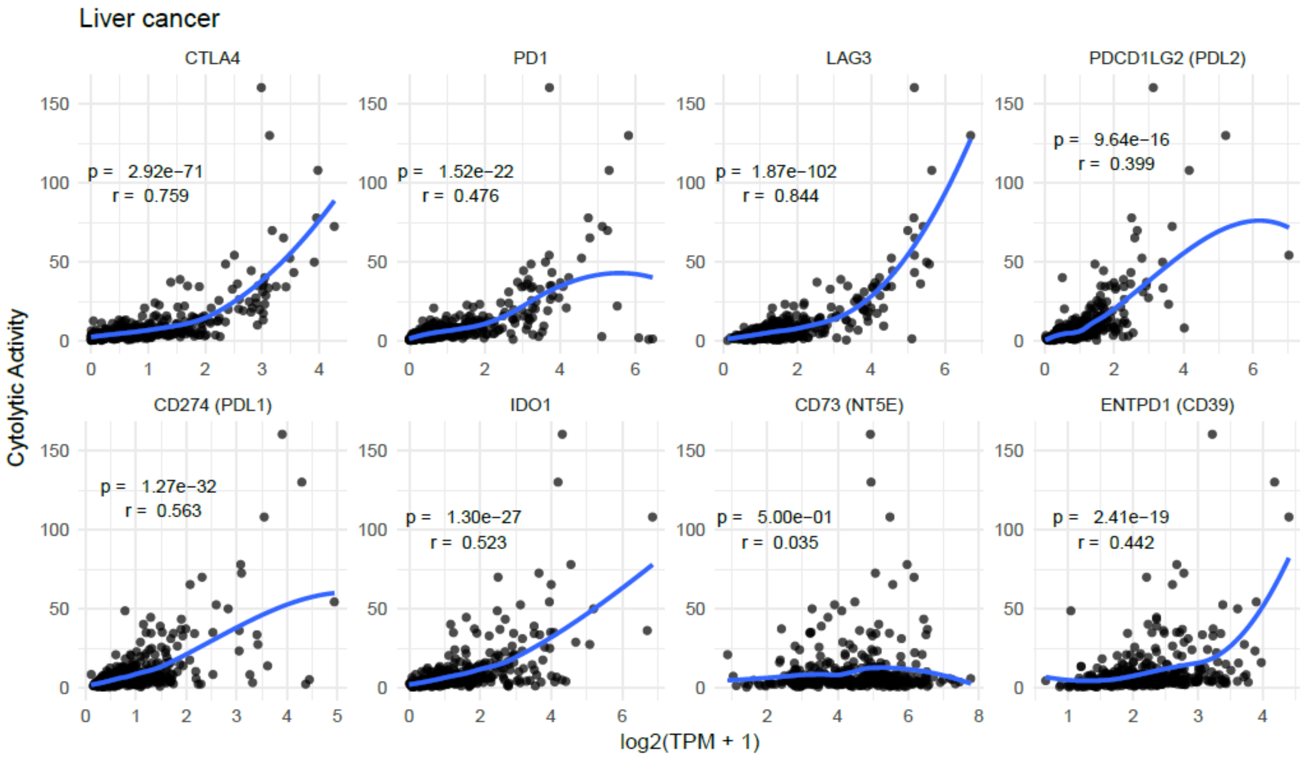
Liver cancer
CTLA4
p = 2.92e−71
r = 0.759
PD1
p = 1.52e−22
r = 0.476
LAG3
p = 1.87e−102
r = 0.844
PDCD1LG2 (PDL2)
p = 9.64e−16
r = 0.399
CD274 (PDL1)
p = 1.27e−32
r = 0.563
IDO1
p = 1.30e−27
r = 0.523
CD73 (NT5E)
p = 5.00e−01
r = 0.035
ENTPD1 (CD39)
p = 2.41e−19
r = 0.442
Cytolytic Activity
log2(TPM + 1)

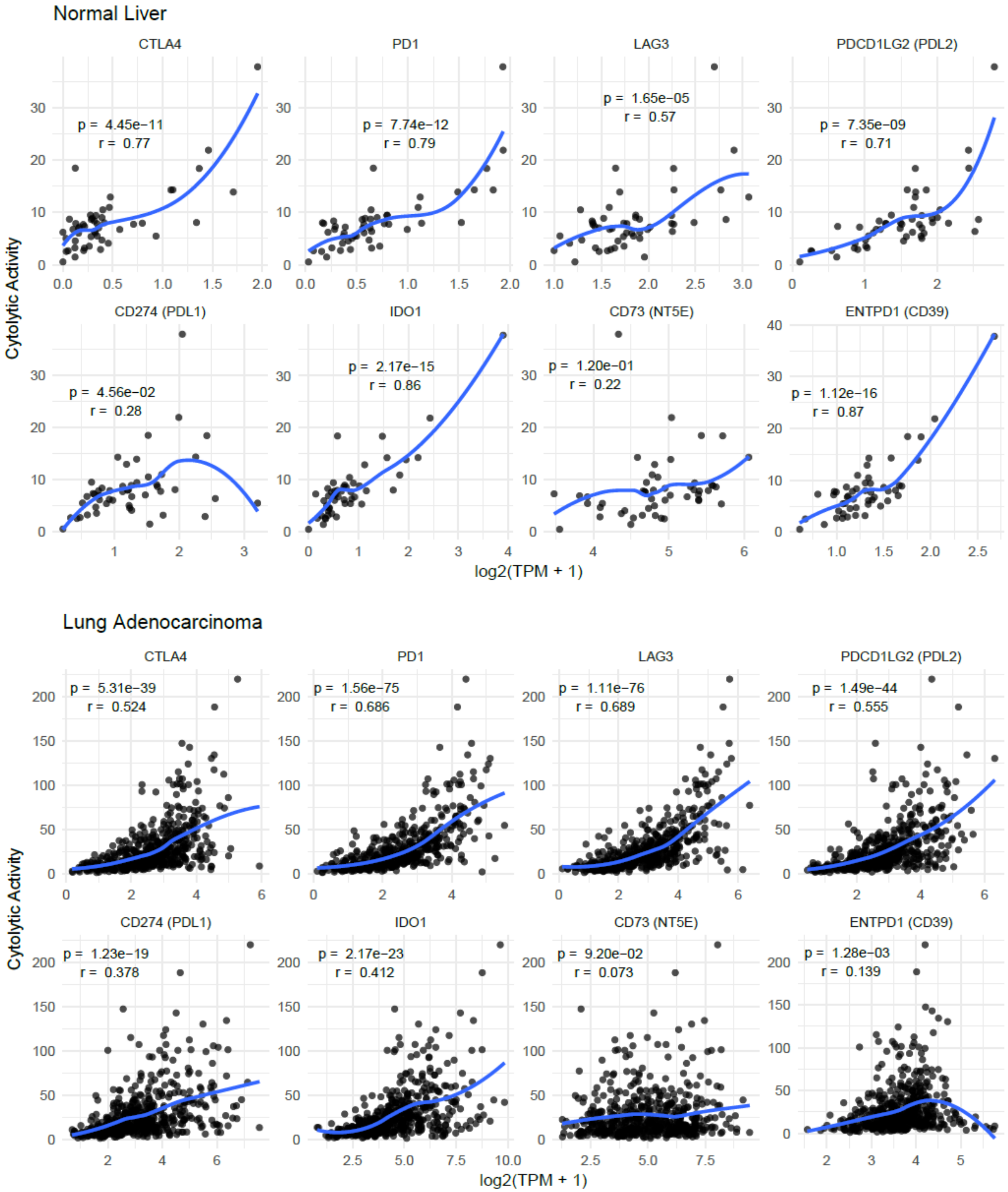
Normal Liver
Cytolytic Activity
CTLA4
p = 4.45e−11
r = 0.77
PD1
p = 7.74e−12
r = 0.79
LAG3
p = 1.65e−05
r = 0.57
PDCD1LG2 (PDL2)
p = 7.35e−09
r = 0.71
CD274 (PDL1)
p = 4.56e−02
r = 0.28
IDO1
p = 2.17e−15
r = 0.86
CD73 (NT5E)
p = 1.20e−01
r = 0.22
ENTPD1 (CD39)
p = 1.12e−16
r = 0.87
log2(TPM + 1)
Lung Adenocarcinoma
Cytolytic Activity
CTLA4
p = 5.31e−39
r = 0.524
PD1
p = 1.56e−75
r = 0.686
LAG3
p = 1.11e−76
r = 0.689
PDCD1LG2 (PDL2)
p = 1.49e−44
r = 0.555
CD274 (PDL1)
p = 1.23e−19
r = 0.378
IDO1
p = 2.17e−23
r = 0.412
CD73 (NT5E)
p = 9.20e−02
r = 0.073
ENTPD1 (CD39)
p = 1.28e−03
r = 0.139
log2(TPM + 1)

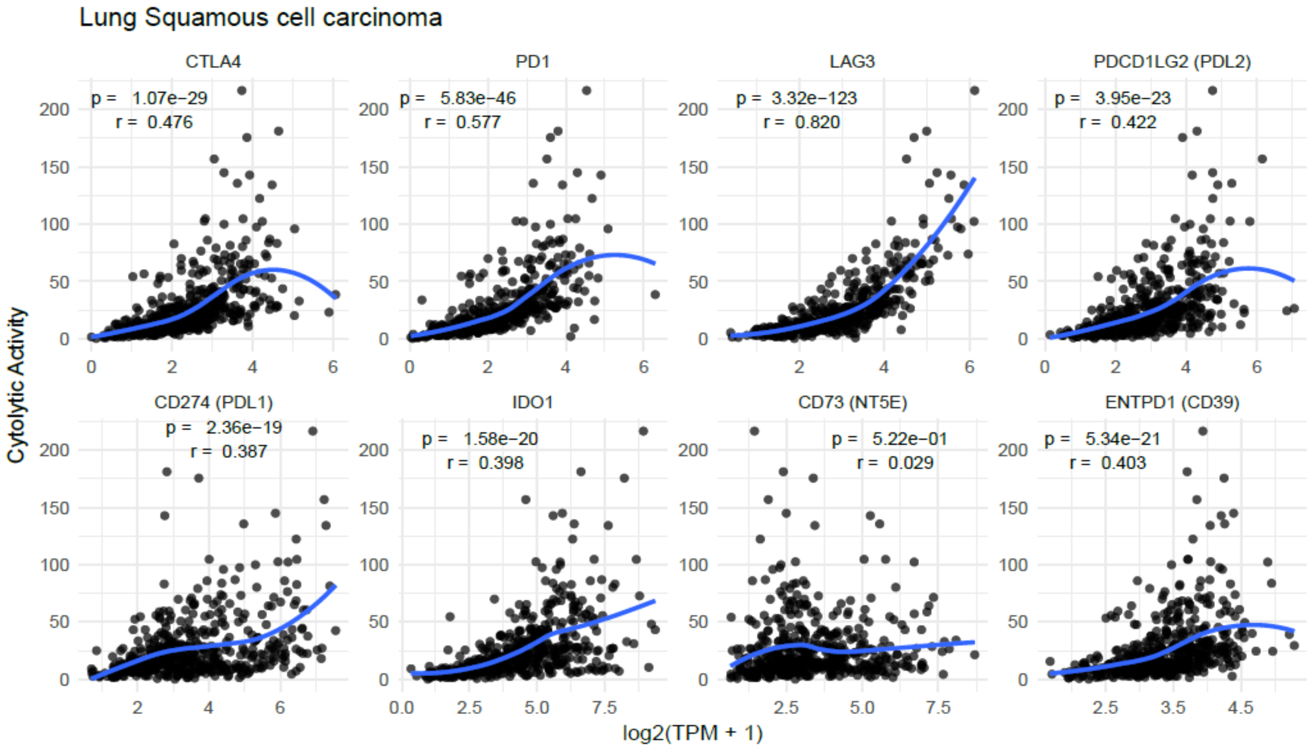
Lung Squamous cell carcinoma
CTLA4
p = 1.07e−29
r = 0.476
PD1
p = 5.83e−46
r = 0.577
LAG3
p = 3.32e−123
r = 0.820
PDCD1LG2 (PDL2)
p = 3.95e−23
r = 0.422
CD274 (PDL1)
p = 2.36e−19
r = 0.387
IDO1
p = 1.58e−20
r = 0.398
CD73 (NT5E)
p = 5.22e−01
r = 0.029
ENTPD1 (CD39)
p = 5.34e−21
r = 0.403
Cytolytic Activity
log2(TPM + 1)

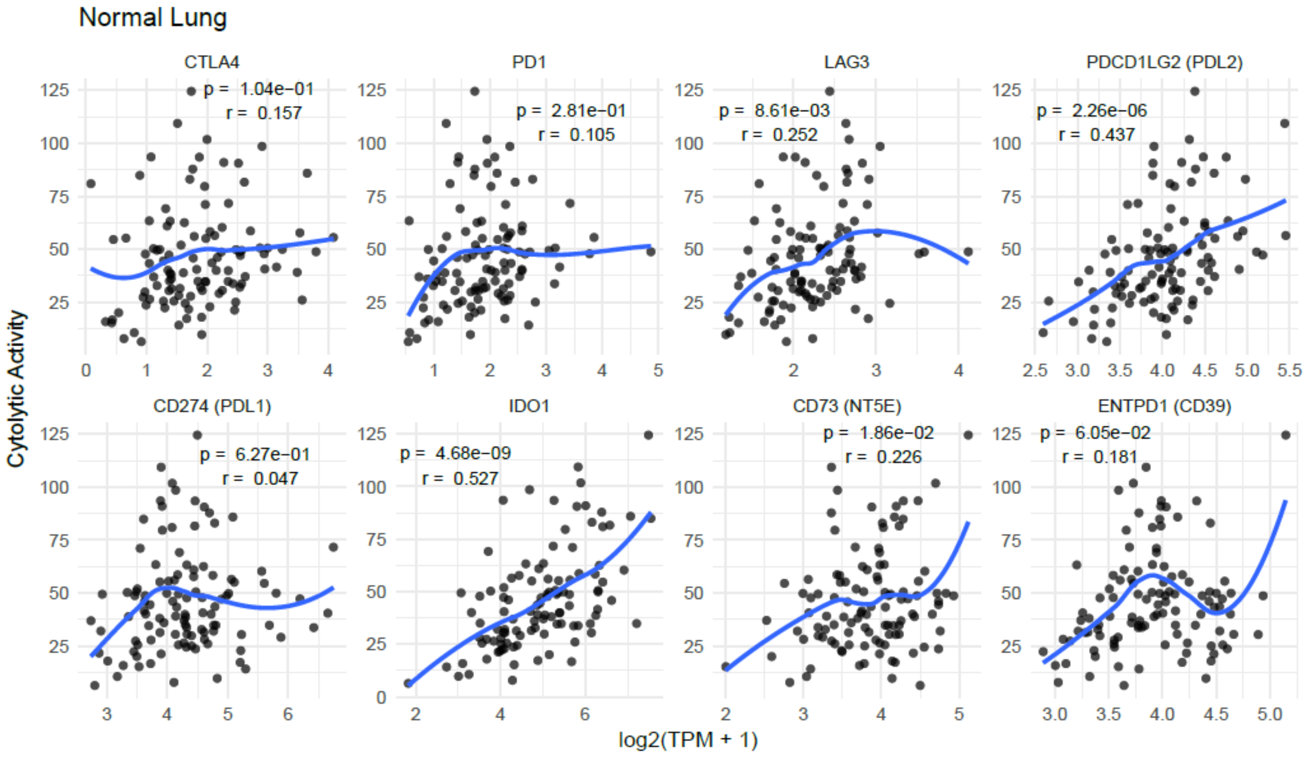
Normal Lung
CTLA4
p = 1.04e−01
r = 0.157
PD1
p = 2.81e−01
r = 0.105
LAG3
p = 8.61e−03
r = 0.252
PDCD1LG2 (PDL2)
p = 2.26e−06
r = 0.437
CD274 (PDL1)
p = 6.27e−01
r = 0.047
IDO1
p = 4.68e−09
r = 0.527
CD73 (NT5E)
p = 1.86e−02
r = 0.226
ENTPD1 (CD39)
p = 6.05e−02
r = 0.181
Cytolytic Activity
log2(TPM + 1)

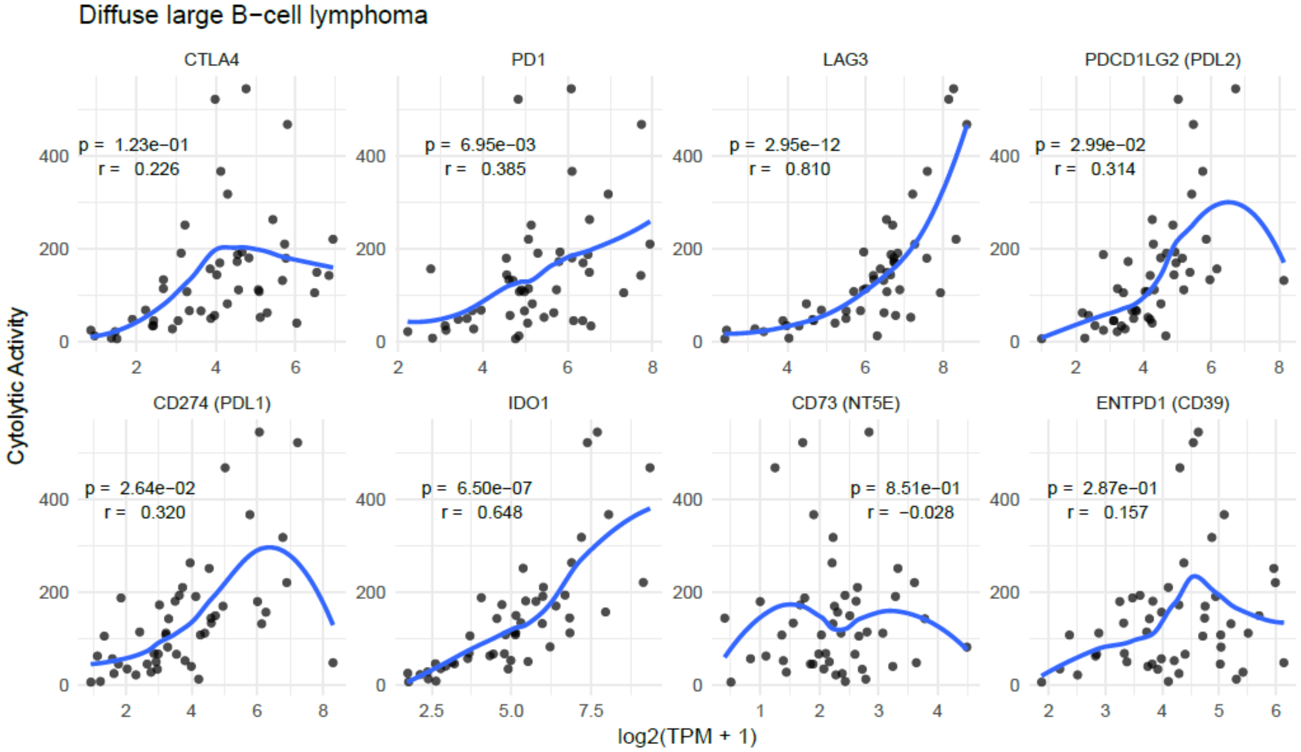
Diffuse large B−cell lymphoma
CTLA4
p = 1.23e−01
r = 0.226
PD1
p = 6.95e−03
r = 0.385
LAG3
p = 2.95e−12
r = 0.810
PDCD1LG2 (PDL2)
p = 2.99e−02
r = 0.314
CD274 (PDL1)
p = 2.64e−02
r = 0.320
IDO1
p = 6.50e−07
r = 0.648
CD73 (NT5E)
p = 8.51e−01
r = −0.028
ENTPD1 (CD39)
p = 2.87e−01
r = 0.157
Cytolytic Activity
log2(TPM + 1)

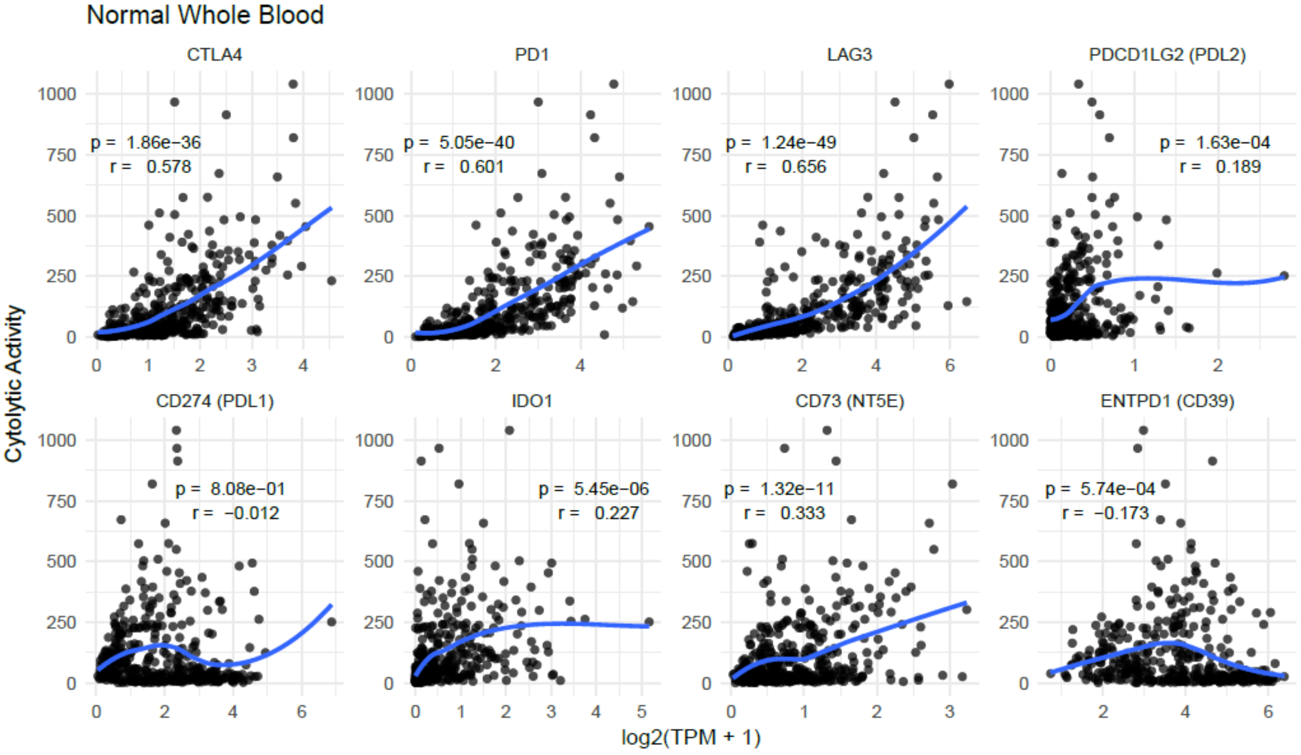
Normal Whole Blood
CTLA4
p = 1.86e−36
r = 0.578
PD1
p = 5.05e−40
r = 0.601
LAG3
p = 1.24e−49
r = 0.656
PDCD1LG2 (PDL2)
p = 1.63e−04
r = 0.189
CD274 (PDL1)
p = 8.08e−01
r = −0.012
IDO1
p = 5.45e−06
r = 0.227
CD73 (NT5E)
p = 1.32e−11
r = 0.333
ENTPD1 (CD39)
p = 5.74e−04
r = −0.173
Cytolytic Activity
log2(TPM + 1)

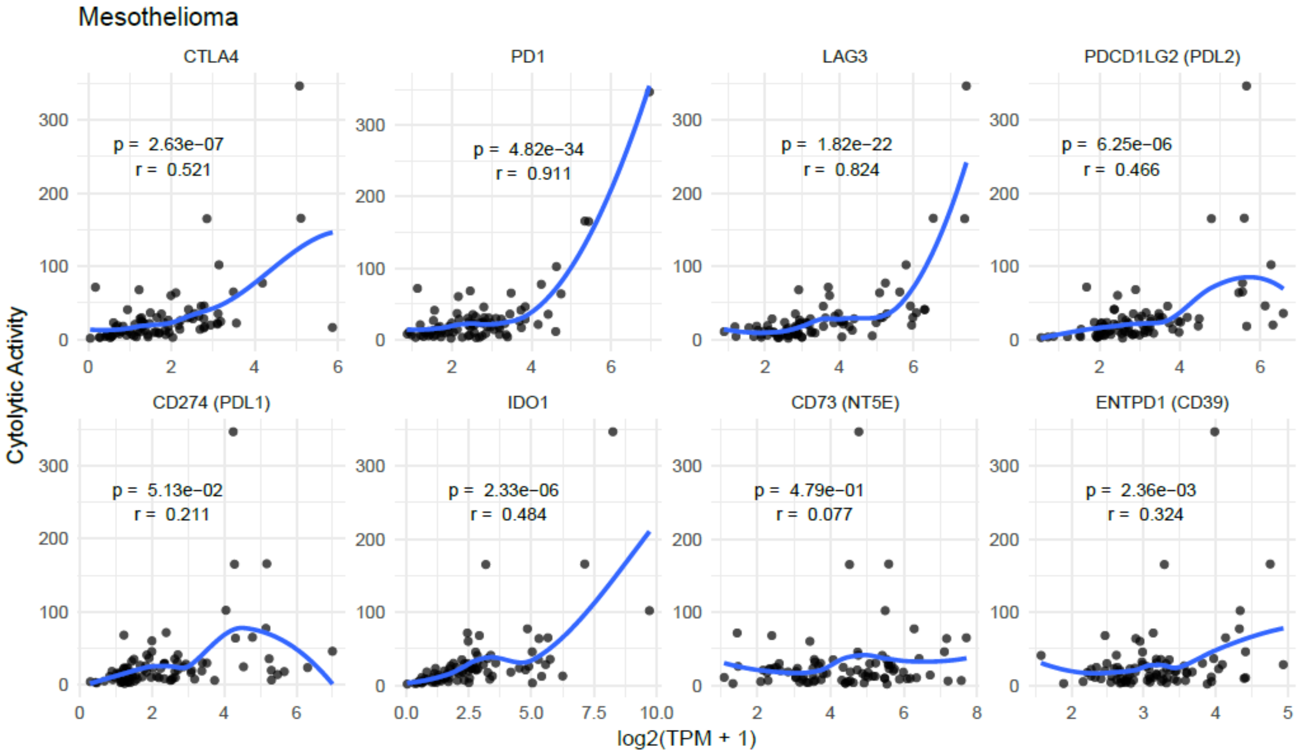
Mesothelioma
CTLA4
p = 2.63e−07
r = 0.521
PD1
p = 4.82e−34
r = 0.911
LAG3
p = 1.82e−22
r = 0.824
PDCD1LG2 (PDL2)
p = 6.25e−06
r = 0.466
CD274 (PDL1)
p = 5.13e−02
r = 0.211
IDO1
p = 2.33e−06
r = 0.484
CD73 (NT5E)
p = 4.79e−01
r = 0.077
ENTPD1 (CD39)
p = 2.36e−03
r = 0.324
Cytolytic Activity
log2(TPM + 1)

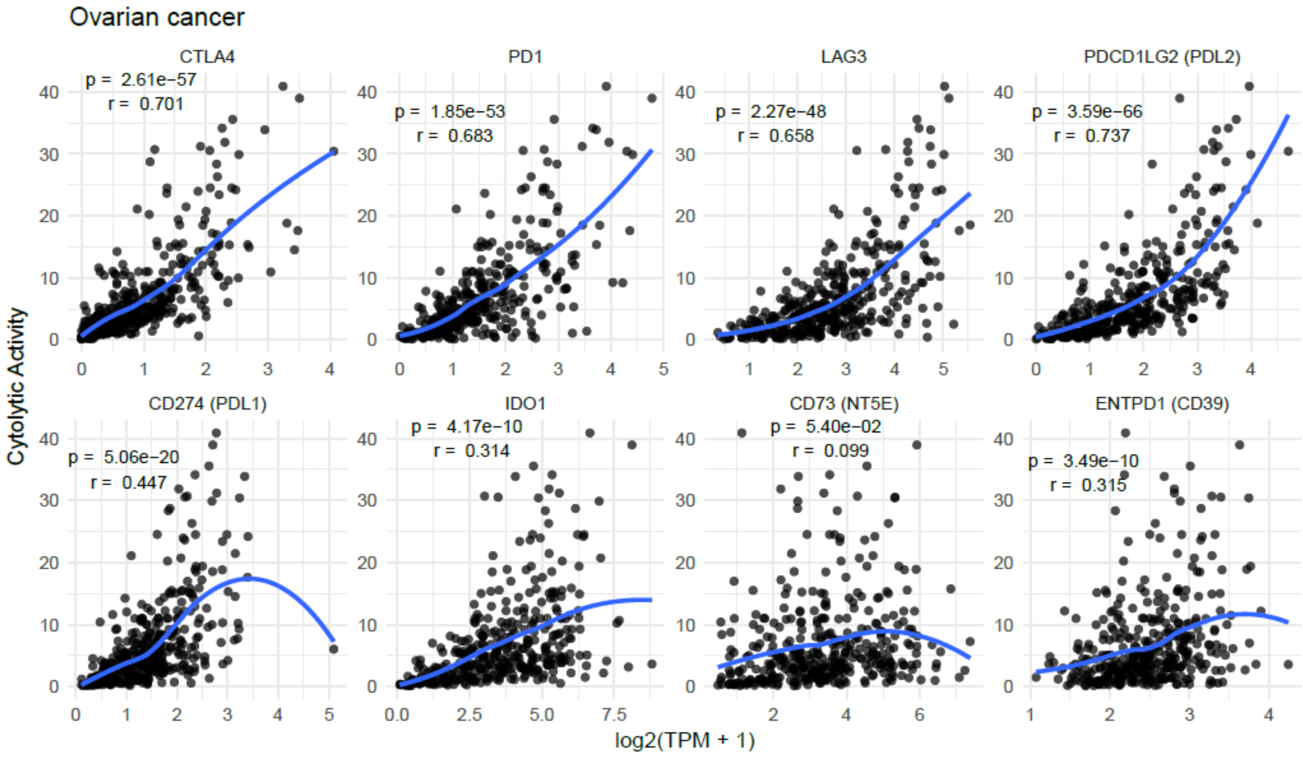
Ovarian cancer
CTLA4
p = 2.61e−57
r = 0.701
PD1
p = 1.85e−53
r = 0.683
LAG3
p = 2.27e−48
r = 0.658
PDCD1LG2 (PDL2)
p = 3.59e−66
r = 0.737
CD274 (PDL1)
p = 5.06e−20
r = 0.447
IDO1
p = 4.17e−10
r = 0.314
CD73 (NT5E)
p = 5.40e−02
r = 0.099
ENTPD1 (CD39)
p = 3.49e−10
r = 0.315
Cytolytic Activity
log2(TPM + 1)

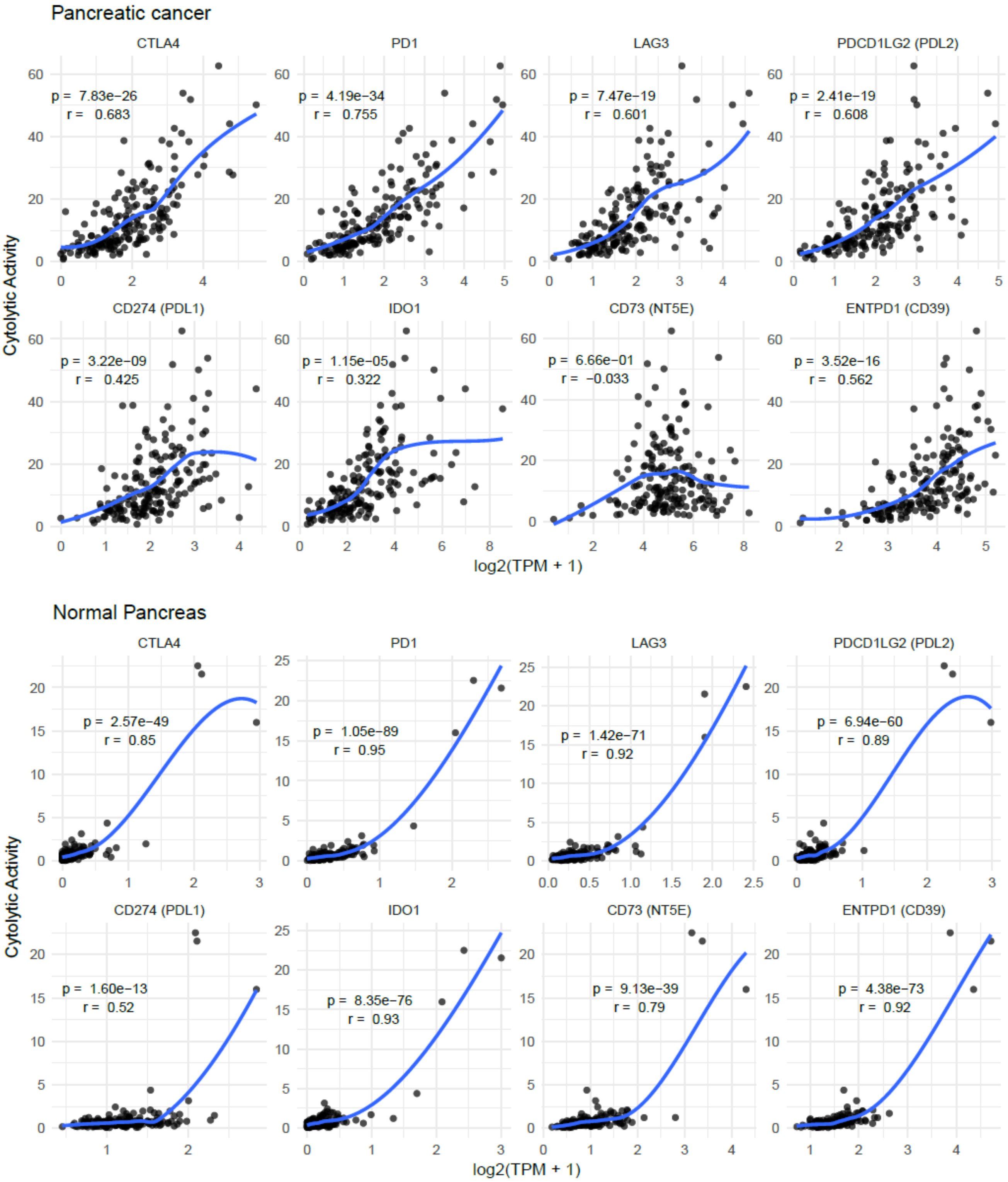
Pancreatic cancer
CTLA4
p = 7.83e−26
r = 0.683
PD1
p = 4.19e−34
r = 0.755
LAG3
p = 7.47e−19
r = 0.601
PDCD1LG2 (PDL2)
p = 2.41e−19
r = 0.608
CD274 (PDL1)
p = 3.22e−09
r = 0.425
IDO1
p = 1.15e−05
r = 0.322
CD73 (NT5E)
p = 6.66e−01
r = −0.033
ENTPD1 (CD39)
p = 3.52e−16
r = 0.562
Cytolytic Activity
log2(TPM + 1)
Normal Pancreas
CTLA4
p = 2.57e−49
r = 0.85
PD1
p = 1.05e−89
r = 0.95
LAG3
p = 1.42e−71
r = 0.92
PDCD1LG2 (PDL2)
p = 6.94e−60
r = 0.89
CD274 (PDL1)
p = 1.60e−13
r = 0.52
IDO1
p = 8.35e−76
r = 0.93
CD73 (NT5E)
p = 9.13e−39
r = 0.79
ENTPD1 (CD39)
p = 4.38e−73
r = 0.92
Cytolytic Activity
log2(TPM + 1)

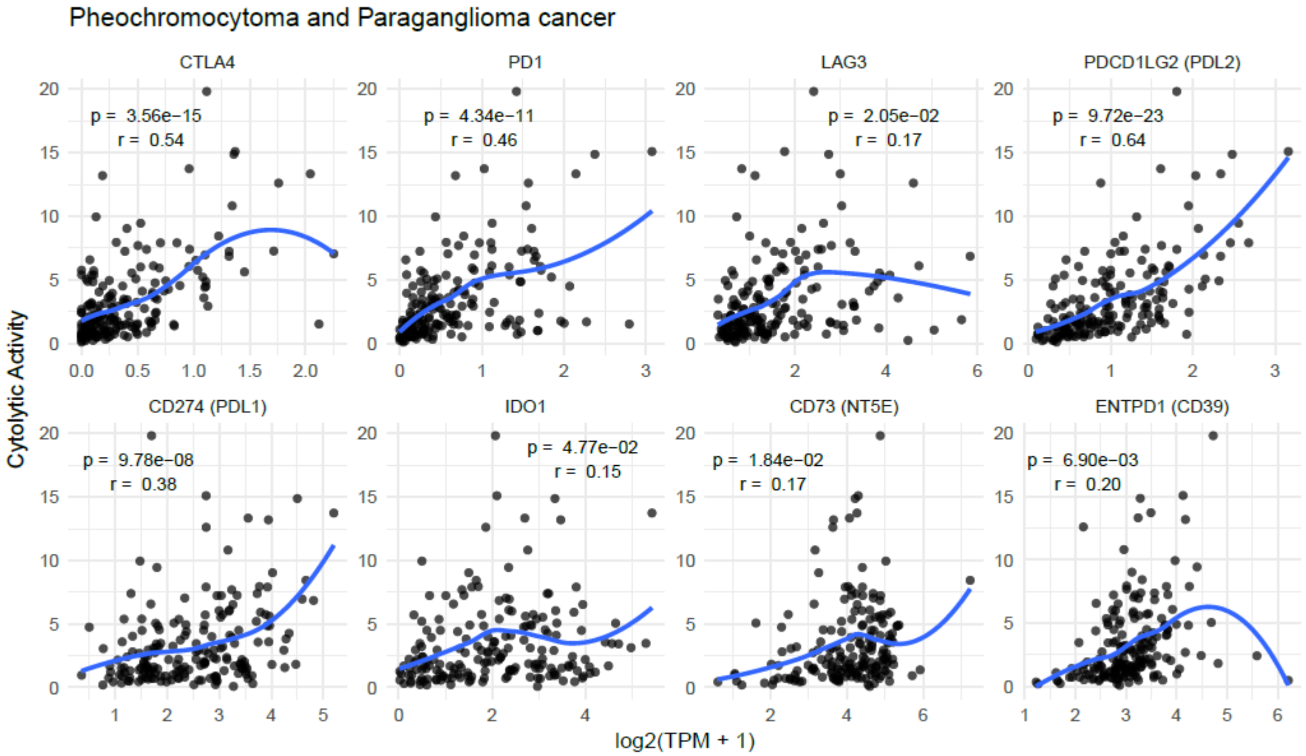
Pheochromocytoma and Paraganglioma cancer
CTLA4
p = 3.56e−15
r = 0.54
PD1
p = 4.34e−11
r = 0.46
LAG3
p = 2.05e−02
r = 0.17
PDCD1LG2 (PDL2)
p = 9.72e−23
r = 0.64
CD274 (PDL1)
p = 9.78e−08
r = 0.38
IDO1
p = 4.77e−02
r = 0.15
CD73 (NT5E)
p = 1.84e−02
r = 0.17
ENTPD1 (CD39)
p = 6.90e−03
r = 0.20
Cytolytic Activity
log2(TPM + 1)

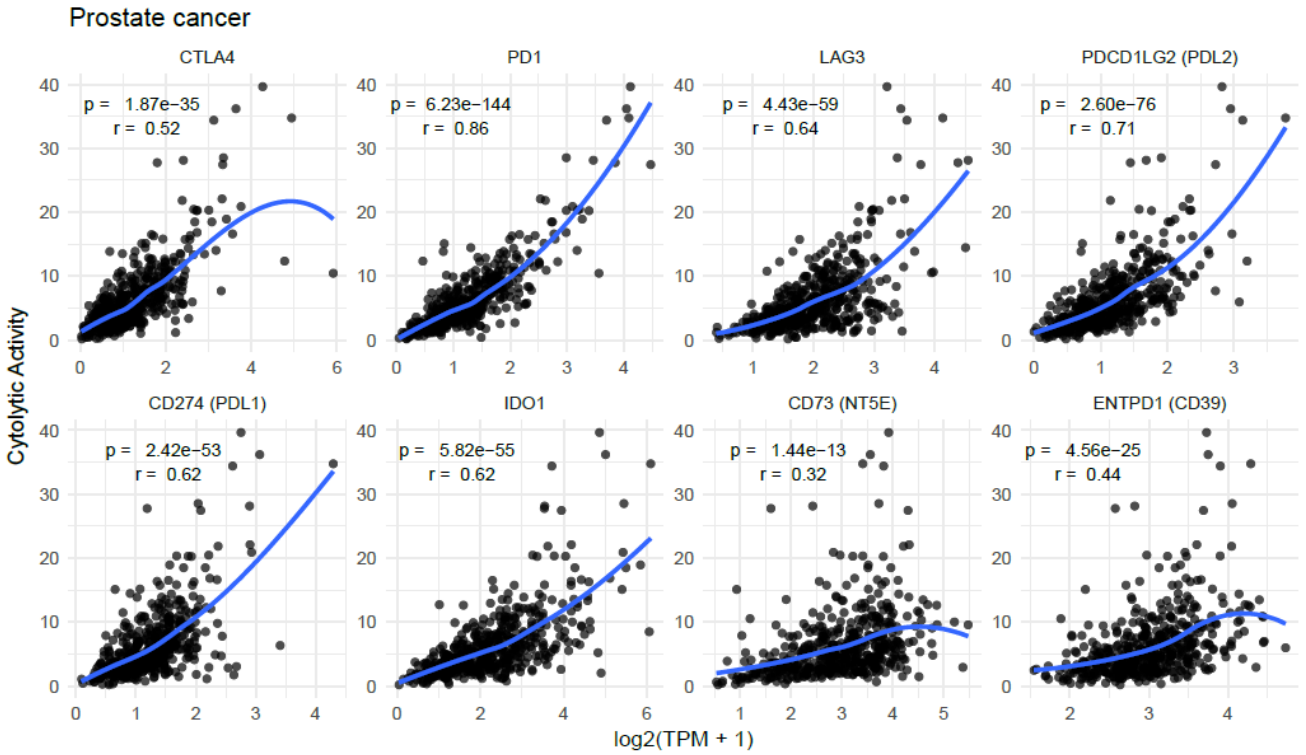
Prostate cancer
CTLA4
p = 1.87e−35
r = 0.52
PD1
p = 6.23e−144
r = 0.86
LAG3
p = 4.43e−59
r = 0.64
PDCD1LG2 (PDL2)
p = 2.60e−76
r = 0.71
CD274 (PDL1)
p = 2.42e−53
r = 0.62
IDO1
p = 5.82e−55
r = 0.62
CD73 (NT5E)
p = 1.44e−13
r = 0.32
ENTPD1 (CD39)
p = 4.56e−25
r = 0.44
Cytolytic Activity
log2(TPM + 1)

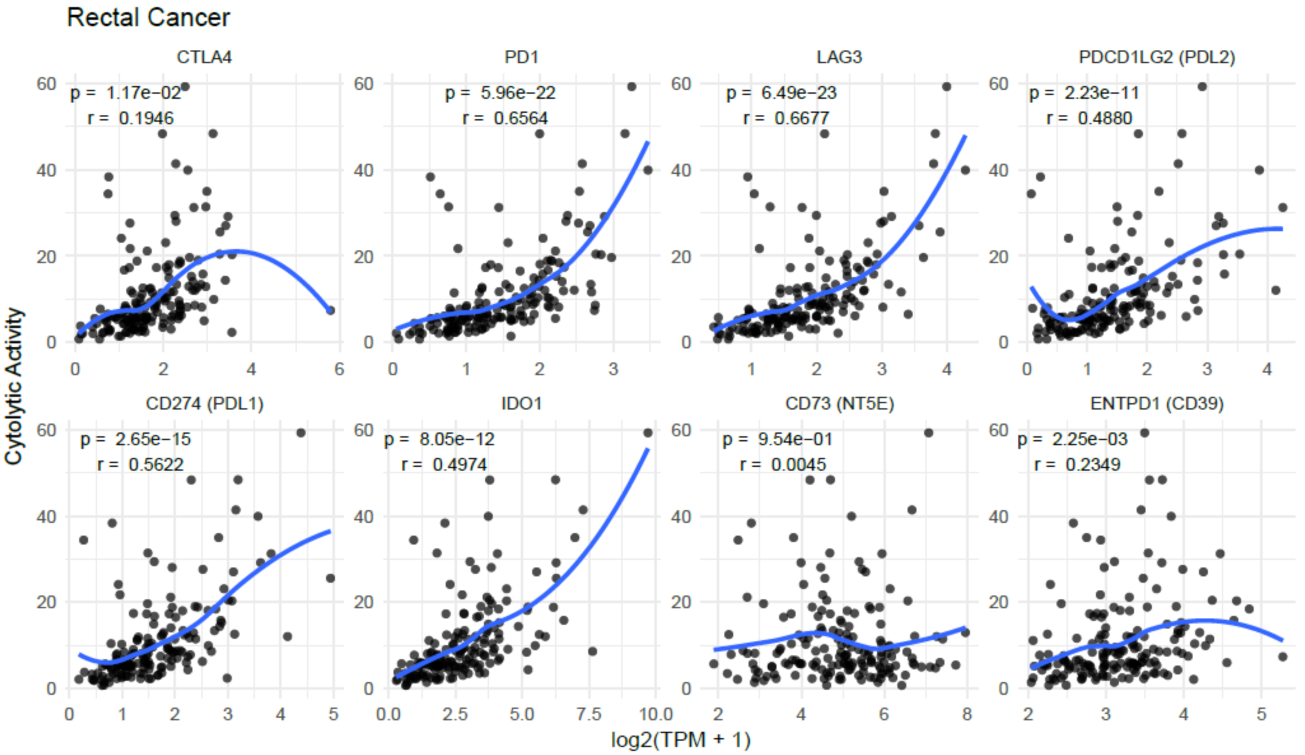
Rectal Cancer
Cytolytic Activity
log2(TPM + 1)
CTLA4
p = 1.17e−02
r = 0.1946
PD1
p = 5.96e−22
r = 0.6564
LAG3
p = 6.49e−23
r = 0.6677
PDCD1LG2 (PDL2)
p = 2.23e−11
r = 0.4880
CD274 (PDL1)
p = 2.65e−15
r = 0.5622
IDO1
p = 8.05e−12
r = 0.4974
CD73 (NT5E)
p = 9.54e−01
r = 0.0045
ENTPD1 (CD39)
p = 2.25e−03
r = 0.2349

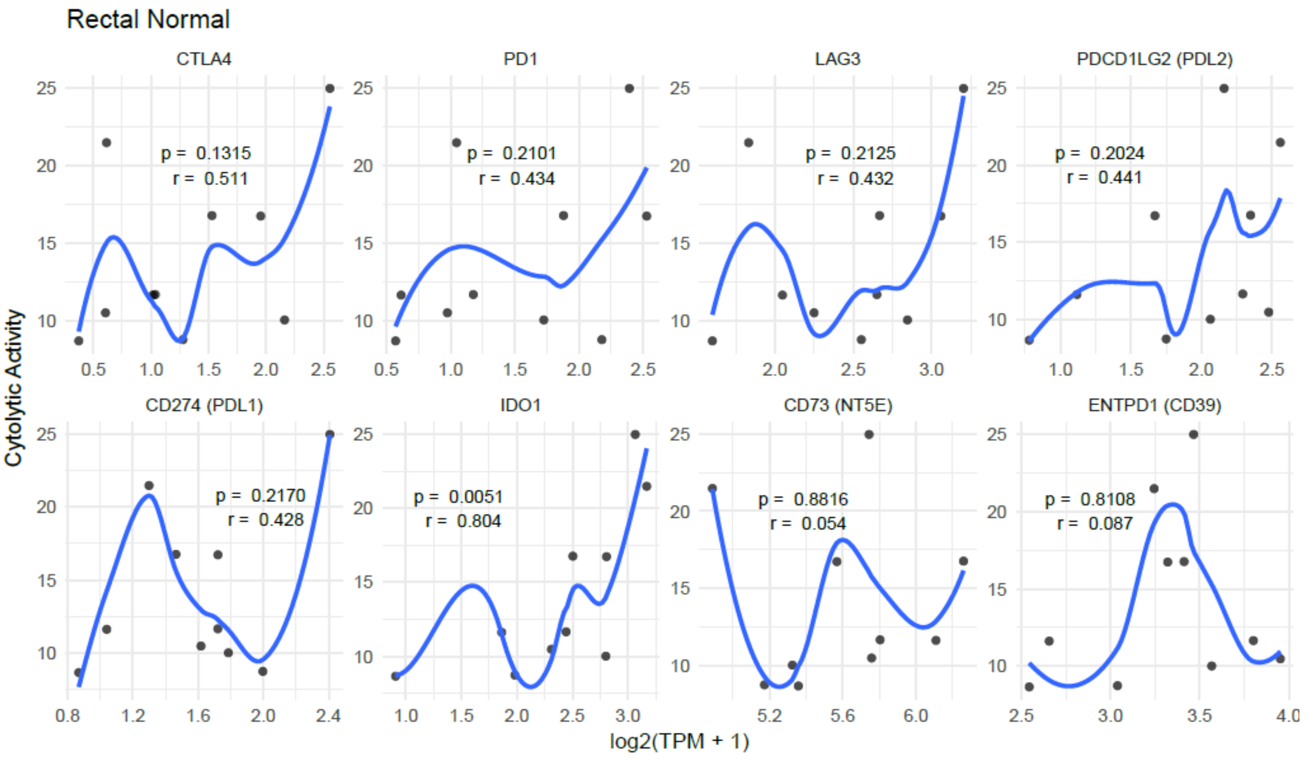
Rectal Normal
Cytolytic Activity
log2(TPM + 1)
CTLA4
p = 0.1315
r = 0.511
PD1
p = 0.2101
r = 0.434
LAG3
p = 0.2125
r = 0.432
PDCD1LG2 (PDL2)
p = 0.2024
r = 0.441
CD274 (PDL1)
p = 0.2170
r = 0.428
IDO1
p = 0.0051
r = 0.804
CD73 (NT5E)
p = 0.8816
r = 0.054
ENTPD1 (CD39)
p = 0.8108
r = 0.087

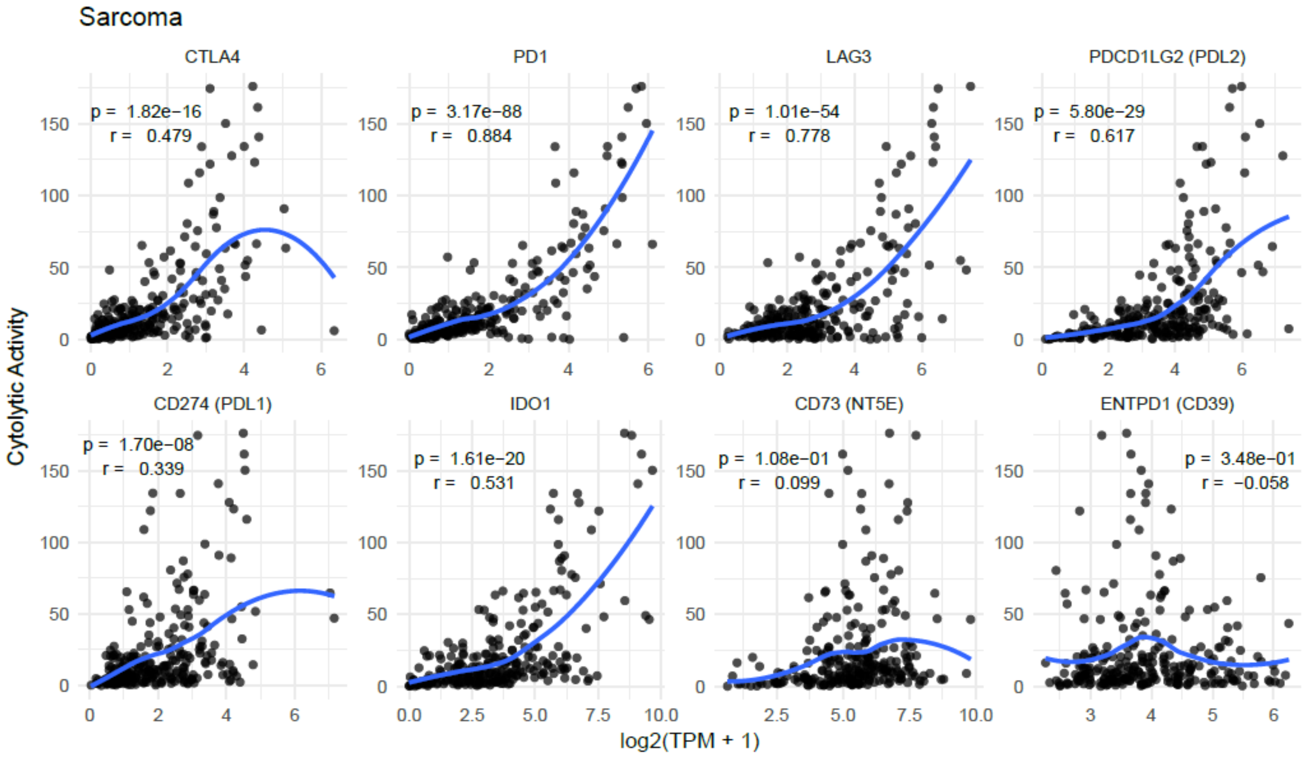
Sarcoma
CTLA4
p = 1.82e−16
r = 0.479
PD1
p = 3.17e−88
r = 0.884
LAG3
p = 1.01e−54
r = 0.778
PDCD1LG2 (PDL2)
p = 5.80e−29
r = 0.617
CD274 (PDL1)
p = 1.70e−08
r = 0.339
IDO1
p = 1.61e−20
r = 0.531
CD73 (NT5E)
p = 1.08e−01
r = 0.099
ENTPD1 (CD39)
p = 3.48e−01
r = −0.058
Cytolytic Activity
log2(TPM + 1)

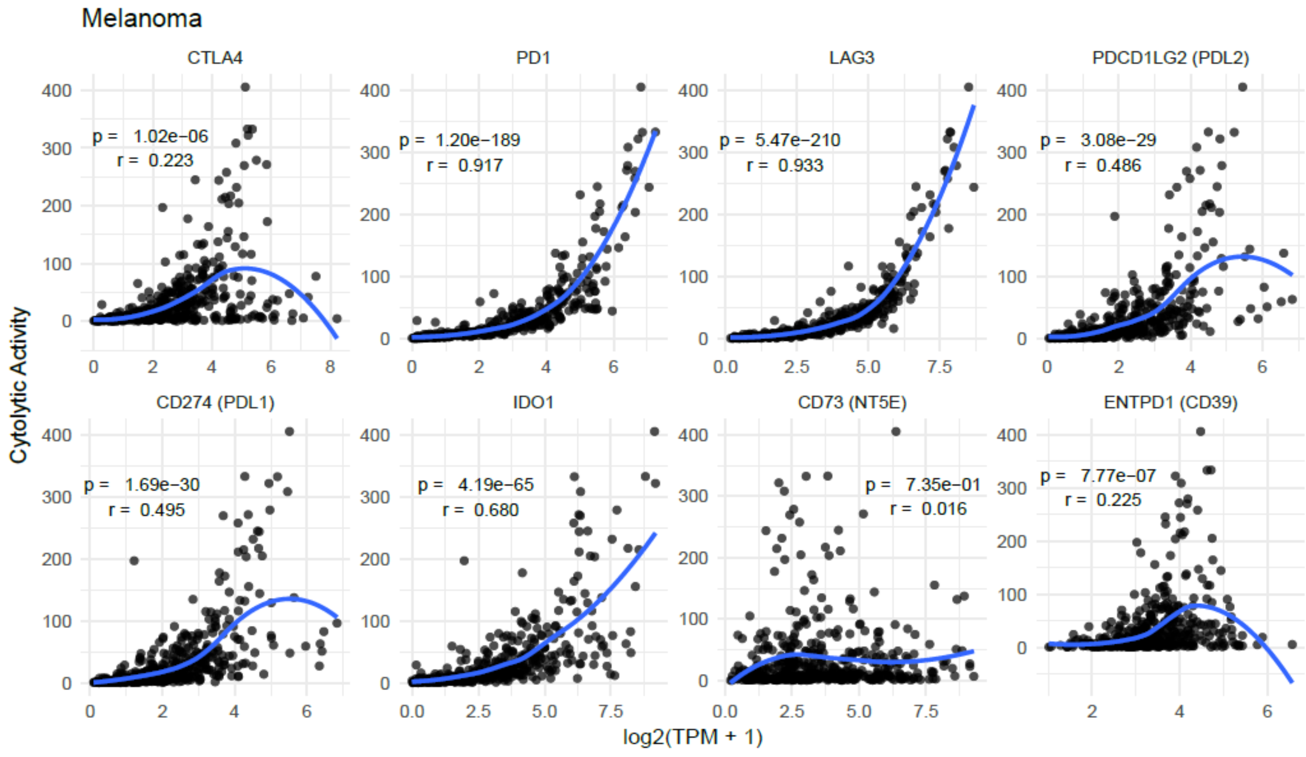
Melanoma
CTLA4
p = 1.02e−06
r = 0.223
PD1
p = 1.20e−189
r = 0.917
LAG3
p = 5.47e−210
r = 0.933
PDCD1LG2 (PDL2)
p = 3.08e−29
r = 0.486
CD274 (PDL1)
p = 1.69e−30
r = 0.495
IDO1
p = 4.19e−65
r = 0.680
CD73 (NT5E)
p = 7.35e−01
r = 0.016
ENTPD1 (CD39)
p = 7.77e−07
r = 0.225
Cytolytic Activity
log2(TPM + 1)

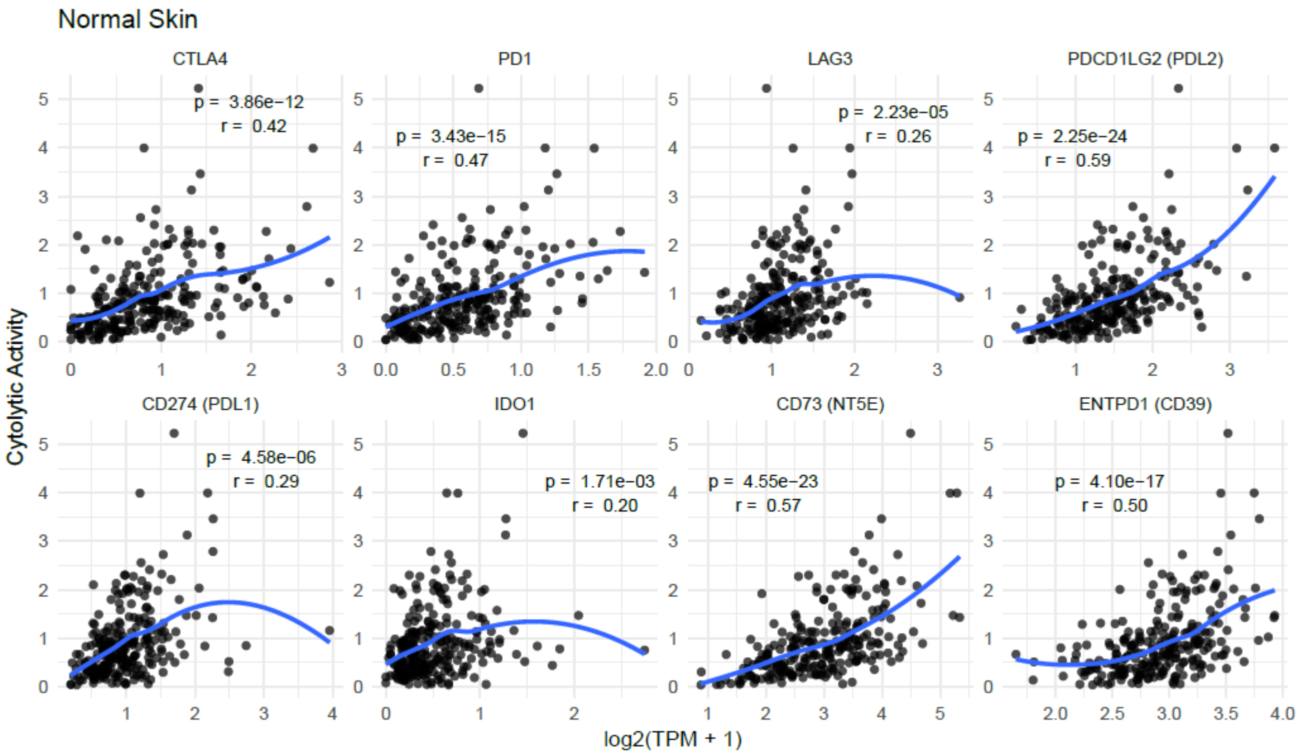
Normal Skin
CTLA4
p = 3.86e−12
r = 0.42
PD1
p = 3.43e−15
r = 0.47
LAG3
p = 2.23e−05
r = 0.26
PDCD1LG2 (PDL2)
p = 2.25e−24
r = 0.59
CD274 (PDL1)
p = 4.58e−06
r = 0.29
IDO1
p = 1.71e−03
r = 0.20
CD73 (NT5E)
p = 4.55e−23
r = 0.57
ENTPD1 (CD39)
p = 4.10e−17
r = 0.50
Cytolytic Activity
log2(TPM + 1)

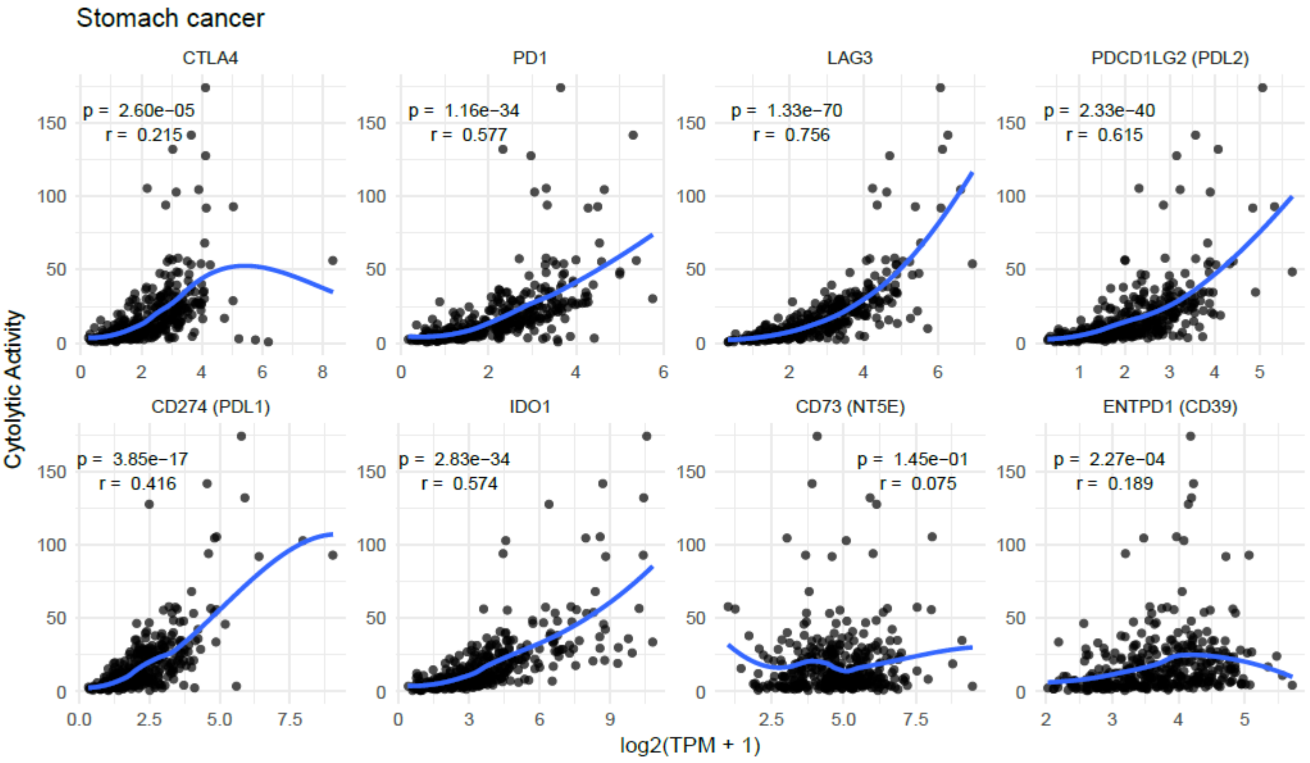
Stomach cancer
CTLA4
p = 2.60e−05
r = 0.215
PD1
p = 1.16e−34
r = 0.577
LAG3
p = 1.33e−70
r = 0.756
PDCD1LG2 (PDL2)
p = 2.33e−40
r = 0.615
CD274 (PDL1)
p = 3.85e−17
r = 0.416
IDO1
p = 2.83e−34
r = 0.574
CD73 (NT5E)
p = 1.45e−01
r = 0.075
ENTPD1 (CD39)
p = 2.27e−04
r = 0.189
Cytolytic Activity
log2(TPM + 1)

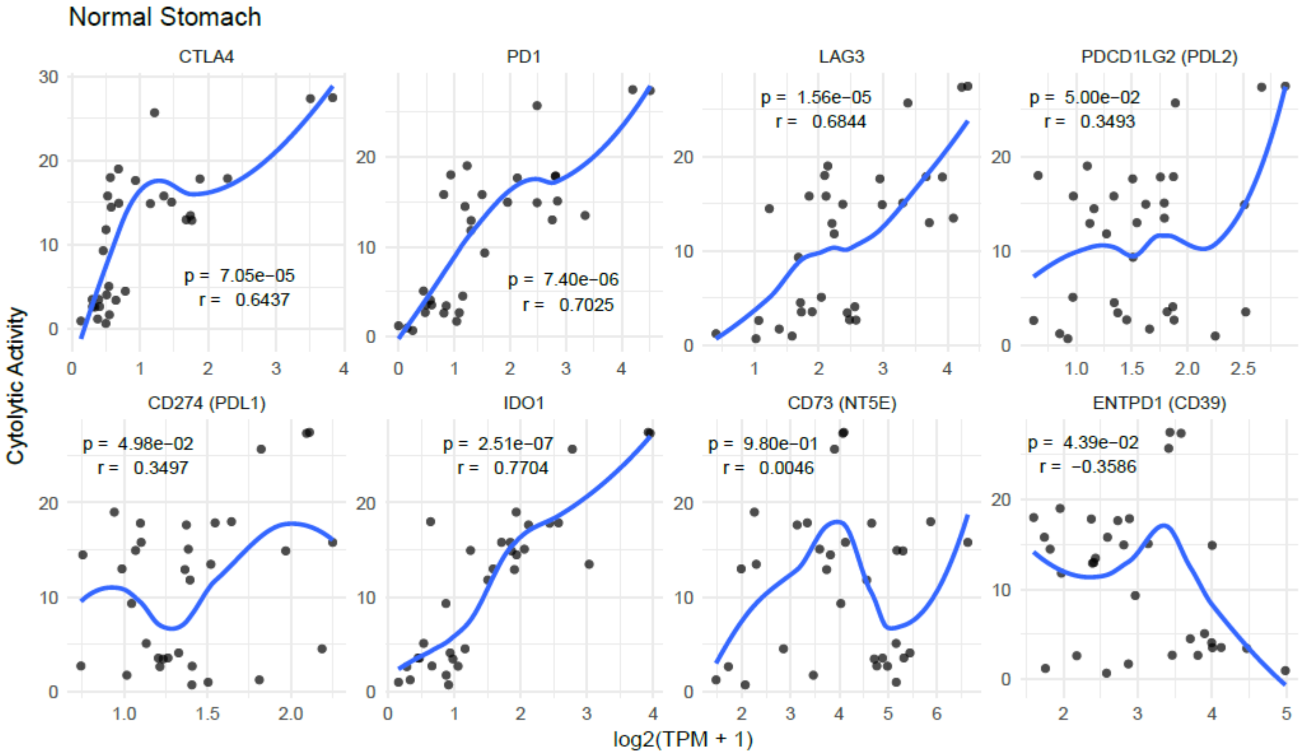
Normal Stomach
CTLA4
p = 7.05e−05
r = 0.6437
PD1
p = 7.40e−06
r = 0.7025
LAG3
p = 1.56e−05
r = 0.6844
PDCD1LG2 (PDL2)
p = 5.00e−02
r = 0.3493
CD274 (PDL1)
p = 4.98e−02
r = 0.3497
IDO1
p = 2.51e−07
r = 0.7704
CD73 (NT5E)
p = 9.80e−01
r = 0.0046
ENTPD1 (CD39)
p = 4.39e−02
r = −0.3586
Cytolytic Activity
log2(TPM + 1)

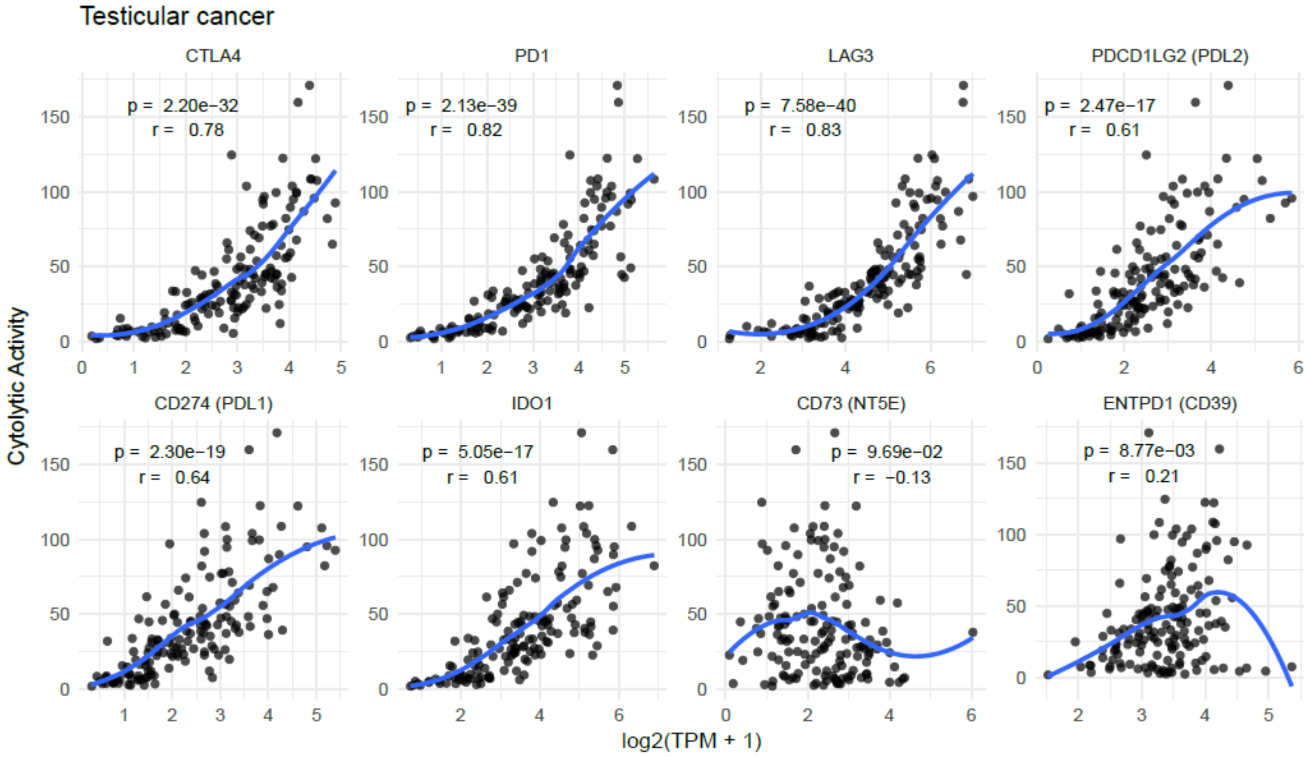
Testicular cancer
CTLA4
p = 2.20e−32
r = 0.78
PD1
p = 2.13e−39
r = 0.82
LAG3
p = 7.58e−40
r = 0.83
PDCD1LG2 (PDL2)
p = 2.47e−17
r = 0.61
CD274 (PDL1)
p = 2.30e−19
r = 0.64
IDO1
p = 5.05e−17
r = 0.61
CD73 (NT5E)
p = 9.69e−02
r = −0.13
ENTPD1 (CD39)
p = 8.77e−03
r = 0.21
Cytolytic Activity
log2(TPM + 1)

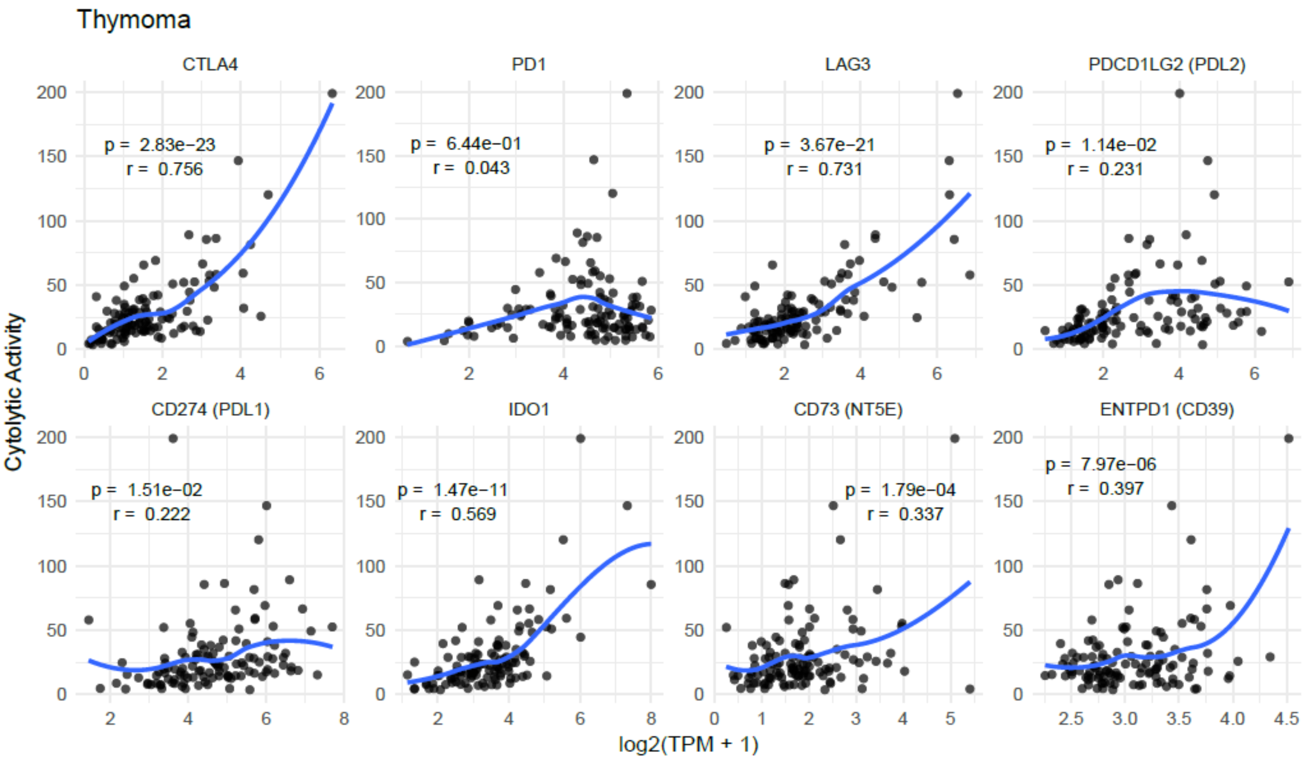
Thymoma
CTLA4
p = 2.83e−23
r = 0.756
PD1
p = 6.44e−01
r = 0.043
LAG3
p = 3.67e−21
r = 0.731
PDCD1LG2 (PDL2)
p = 1.14e−02
r = 0.231
CD274 (PDL1)
p = 1.51e−02
r = 0.222
IDO1
p = 1.47e−11
r = 0.569
CD73 (NT5E)
p = 1.79e−04
r = 0.337
ENTPD1 (CD39)
p = 7.97e−06
r = 0.397
Cytolytic Activity
log2(TPM + 1)

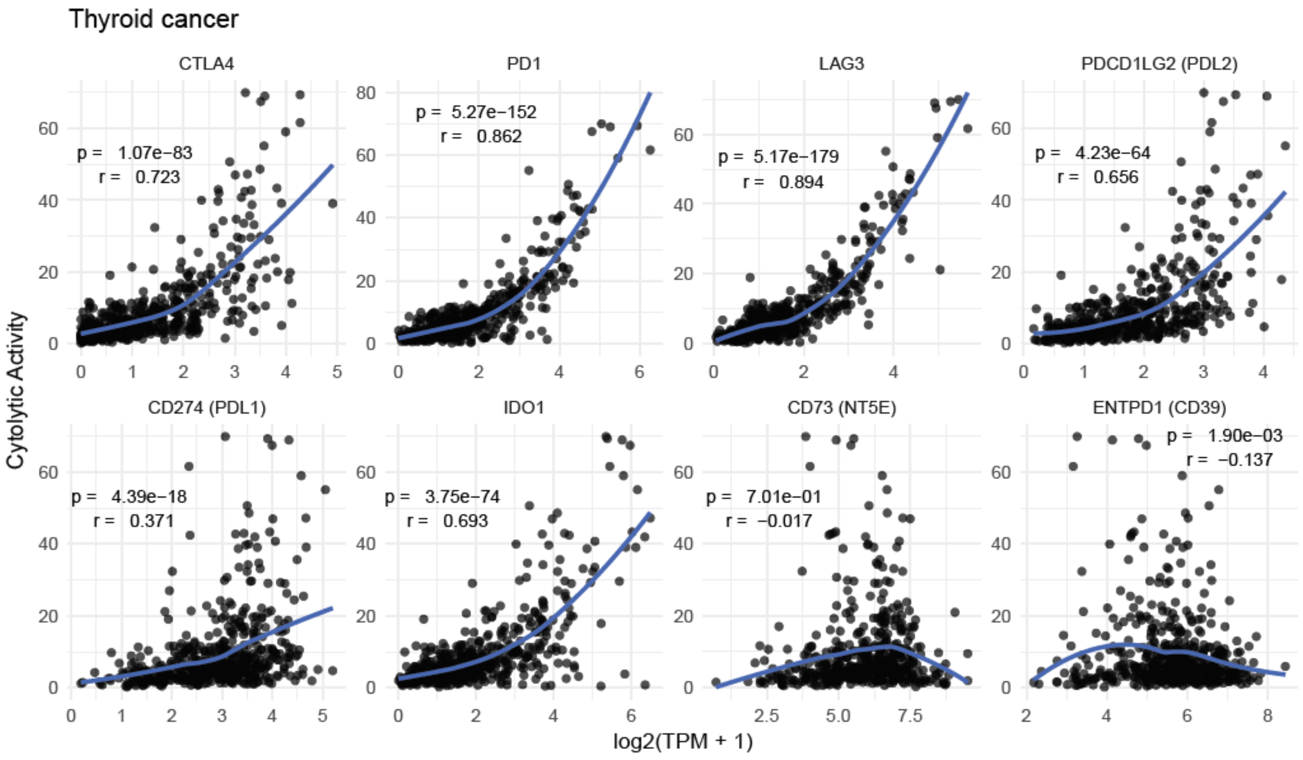
Thyroid cancer
CTLA4
p = 1.07e−83
r = 0.723
PD1
p = 5.27e−152
r = 0.862
LAG3
p = 5.17e−179
r = 0.894
PDCD1LG2 (PDL2)
p = 4.23e−64
r = 0.656
CD274 (PDL1)
p = 4.39e−18
r = 0.371
IDO1
p = 3.75e−74
r = 0.693
CD73 (NT5E)
p = 7.01e−01
r = −0.017
ENTPD1 (CD39)
p = 1.90e−03
r = −0.137
Cytolytic Activity
log2(TPM + 1)

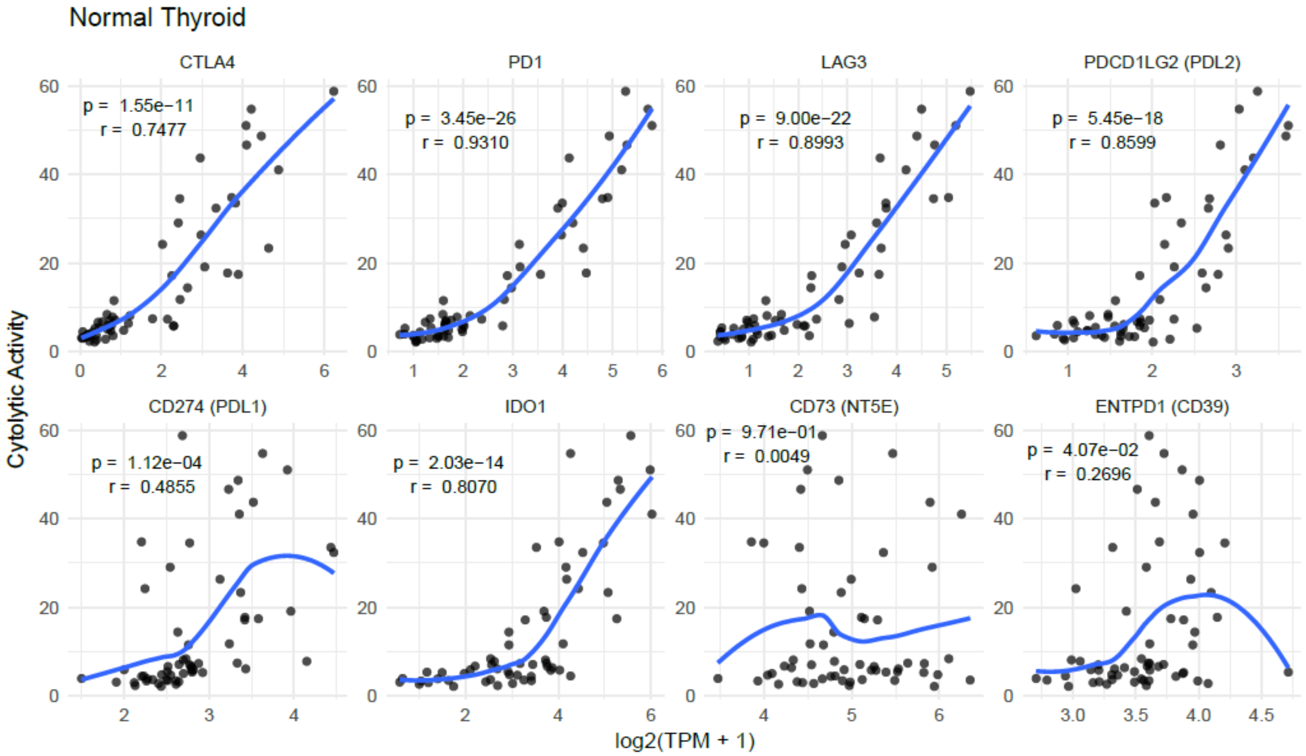
Normal Thyroid
CTLA4
p = 1.55e−11
r = 0.7477
PD1
p = 3.45e−26
r = 0.9310
LAG3
p = 9.00e−22
r = 0.8993
PDCD1LG2 (PDL2)
p = 5.45e−18
r = 0.8599
CD274 (PDL1)
p = 1.12e−04
r = 0.4855
IDO1
p = 2.03e−14
r = 0.8070
CD73 (NT5E)
p = 9.71e−01
r = 0.0049
ENTPD1 (CD39)
p = 4.07e−02
r = 0.2696
Cytolytic Activity
log2(TPM + 1)

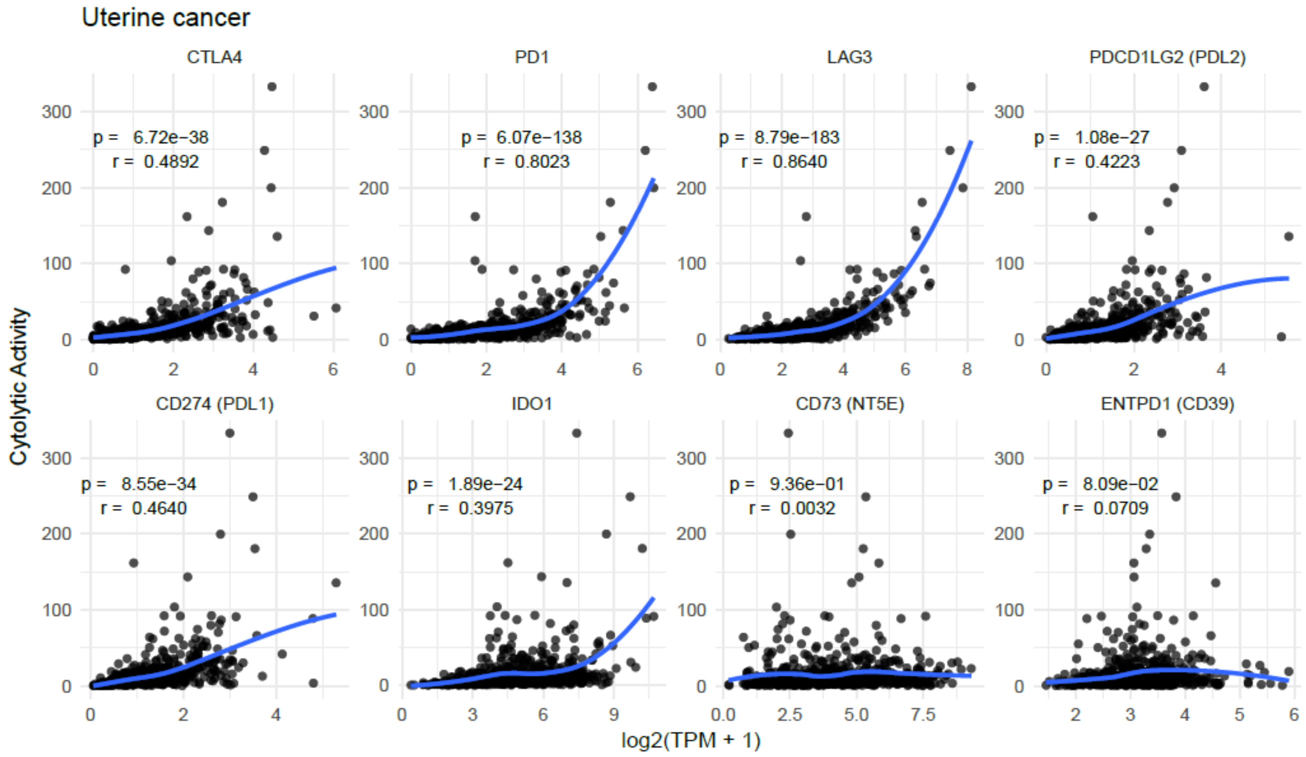
Uterine cancer
CTLA4
p = 6.72e−38
r = 0.4892
PD1
p = 6.07e−138
r = 0.8023
LAG3
p = 8.79e−183
r = 0.8640
PDCD1LG2 (PDL2)
p = 1.08e−27
r = 0.4223
CD274 (PDL1)
p = 8.55e−34
r = 0.4640
IDO1
p = 1.89e−24
r = 0.3975
CD73 (NT5E)
p = 9.36e−01
r = 0.0032
ENTPD1 (CD39)
p = 8.09e−02
r = 0.0709
Cytolytic Activity
log2(TPM + 1)

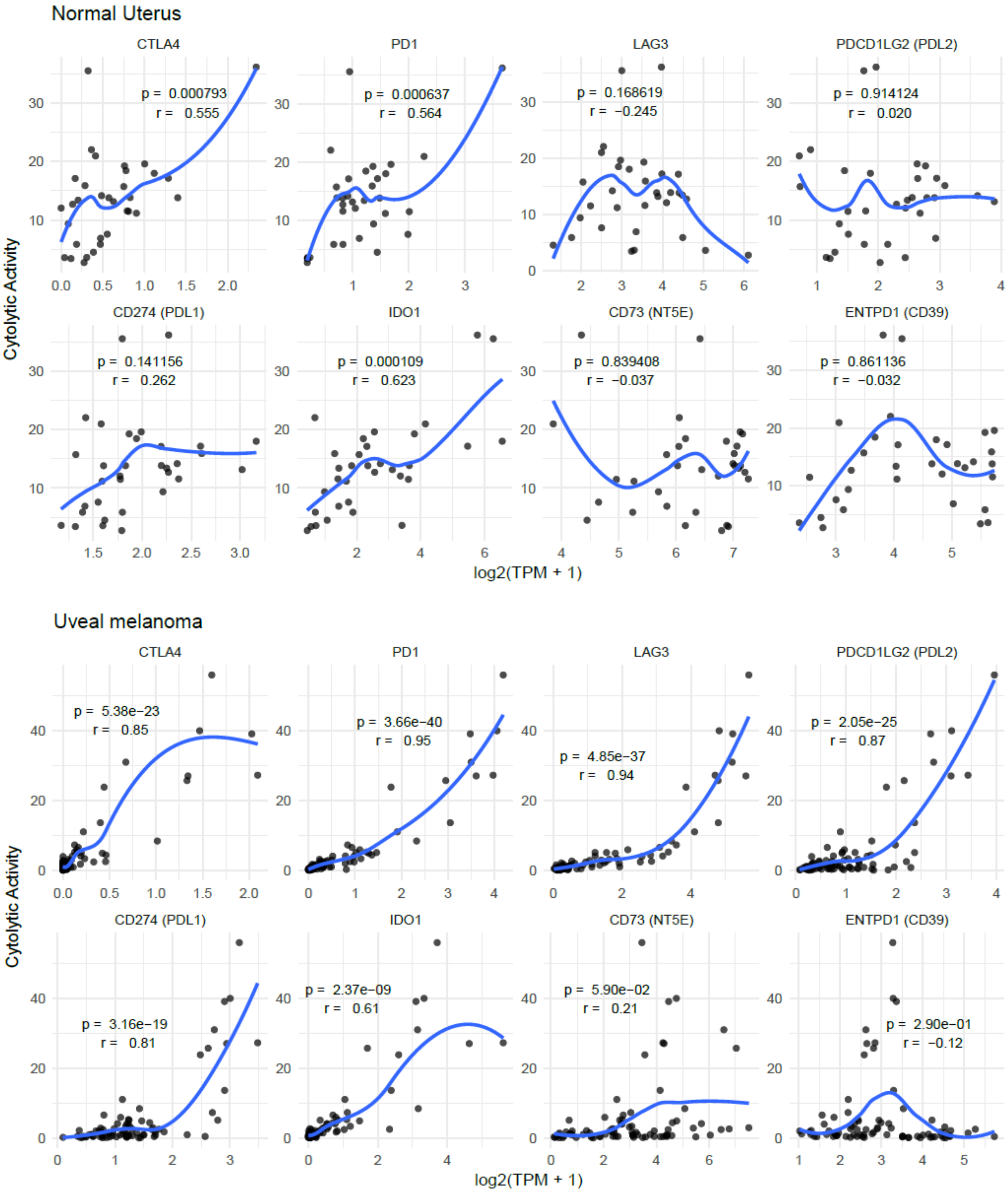


**Figure S7.** Local regression curves between cytolytic activity (CYT, measured as the geometric average of GZMA and PRF1) and the expression of the immune-checkpoint molecules CTLA-4, PD-1, PD-L1/2, LAG3, IDO1, CD73 and CD39 across all TCGA (cancers) and GTEx (normals) datasets. In the majority of the cancers, the correlation between CYT and the expression of the investigated immune-checkpoint molecules was more significant compared to the corresponding normal tissues.

The Pearson's rho and statistical significance (p-value) is indicated in each graph. Loess regression (blue line) was used to diminish the noise of the variables during correlation analysis.
