# Supplementary material for: The Expression and Prognostic Impact of Immune Cytolytic Activity-Related Markers in Human Malignancies: A Comprehensive Meta-analysis
Source: Front Oncol. 2018 Feb 21;8:27. doi: 10.3389/fonc.2018.00027 (PMC5826382; doi:10.3389/fonc.2018.00027)
Supplement: Supplementary file 8 [file image_8.PDF]

Supplementary Material

Title: The expression and prognostic impact of immune cytolytic activity-related markers in human malignancies: A comprehensive meta-analysis

Constantinos Roufas [1,2], Dimitrios Chasiotis [1], Anestis Makris [1], Christodoulos Efstathiades [2], Christos Dimopoulos [2], Apostolos Zaravinos [1,*]

[1] Department of Life Sciences, Biomedical Sciences Program, School of Sciences, European University Cyprus, Nicosia, Cyprus.

[2] The Center for Risk and Decision Sciences (CERIDES), Department of Computer Sciences, School of Sciences, European University Cyprus, Nicosia, Cyprus.

*** Correspondence: Apostolos Zaravinos, PhD. Biomedical Sciences Program, Department of Life Sciences, School of Sciences, European University Cyprus. 6, Diogenes Str. Engomi, P.O. Box 22006, 1516, Nicosia, Cyprus. Tel: +357-22559577. Email: a.zaravinos@euc.ac.cy**

Supplementary Figures

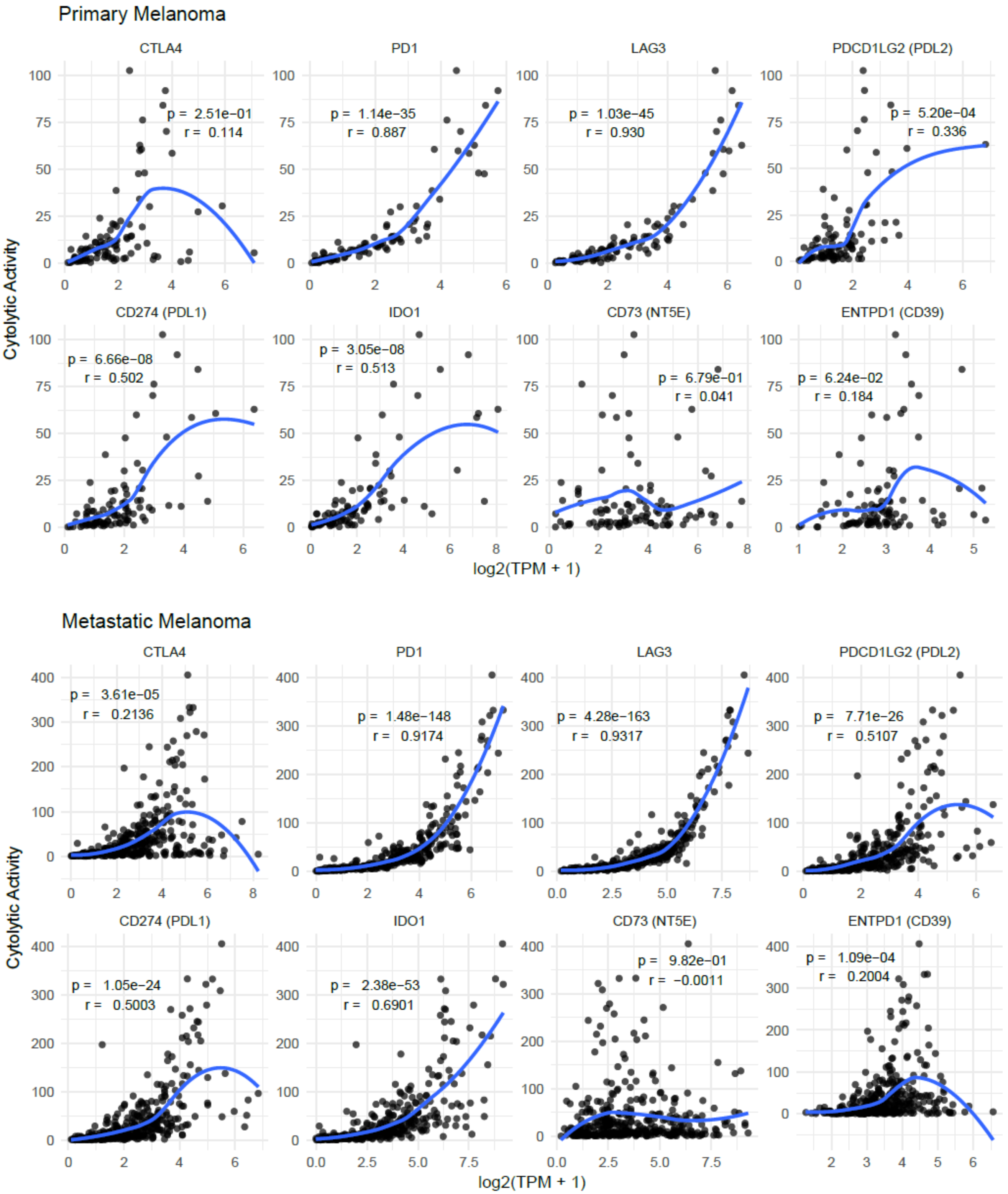

Figure S8. Local regression curves between cytolytic actity (CYT) and the expression levels of immune checkpoint molecules in primary and metastatic melanoma. In the TCGA-SKCM dataset, the correlation between CYT and the expression of various immune-checkpoint molecules was more significant in metastatic melanomas than in primary melanomas. The Pearson's rho and statistical significance (p-value) is indicated in each graph. Loess regression (blue line) was used to diminish the noise of the variables during correlation analysis.
